# Decreased miR-26a Expression Correlates with the Progression of Podocyte Injury in Autoimmune Glomerulonephritis

**DOI:** 10.1371/journal.pone.0110383

**Published:** 2014-10-17

**Authors:** Osamu Ichii, Saori Otsuka-Kanazawa, Taro Horino, Junpei Kimura, Teppei Nakamura, Manabu Matsumoto, Makoto Toi, Yasuhiro Kon

**Affiliations:** 1 Laboratory of Anatomy, Department of Biomedical Sciences, Graduate School of Veterinary Medicine, Hokkaido University, Sapporo, Hokkaido, Japan; 2 Department of Endocrinology, Metabolism and Nephrology, Kochi Medical School, Kochi University, Nankoku, Kochi, Japan; 3 Section of Biological Safety Research, Chitose Laboratory, Japan Food Research Laboratories, Chitose, Hokkaido, Japan; 4 Department of Diagnostic Pathology, Kochi Medical School, Kochi University, Nankoku, Kochi, Japan; Fondazione IRCCS Ospedale Maggiore Policlinico & Fondazione D’Amico per la Ricerca sulle Malattie Renali, Italy

## Abstract

MicroRNAs contribute to the pathogenesis of certain diseases and may serve as biomarkers. We analyzed glomerular microRNA expression in B6.MRLc1, which serve as a mouse model of autoimmune glomerulonephritis. We found that miR-26a was the most abundantly expressed microRNA in the glomerulus of normal C57BL/6 and that its glomerular expression in B6.MRLc1 was significantly lower than that in C57BL/6. In mouse kidneys, podocytes mainly expressed miR-26a, and glomerular miR-26a expression in B6.MRLc1 mice correlated negatively with the urinary albumin levels and podocyte-specific gene expression. Puromycin-induced injury of immortalized mouse podocytes decreased miR-26a expression, perturbed the actin cytoskeleton, and increased the release of exosomes containing miR-26a. Although miR-26a expression increased with differentiation of immortalized mouse podocytes, silencing miR-26a decreased the expression of genes associated with the podocyte differentiation and formation of the cytoskeleton. In particular, the levels of vimentin and actin significantly decreased. In patients with lupus nephritis and IgA nephropathy, glomerular miR-26a levels were significantly lower than those of healthy controls. In B6.MRLc1 and patients with lupus nephritis, miR-26a levels in urinary exosomes were significantly higher compared with those for the respective healthy control. These data indicate that miR-26a regulates podocyte differentiation and cytoskeletal integrity, and its altered levels in glomerulus and urine may serve as a marker of injured podocytes in autoimmune glomerulonephritis.

## Introduction

MicroRNAs (miRNAs) are small noncoding RNAs that act as transcriptional and posttranscriptional regulators and are specifically expressed by certain organs or cell types. For example, miR-192 and miR-194 are abundantly expressed in normal human kidneys, and their levels in rats are higher in the renal cortex than in the medulla [1], [2]. miRNAs are also present in urine [3]. Urinary miRNAs are stable because they are encapsulated in exosomes, which are vesicles secreted by cells of the nephron and may serve as biomarkers [4]. For example, elevated levels of urinary miR-146a and miR-155 are present in patients with systemic lupus erythematosus (SLE) [5].

Lupus nephritis is characterized by autoimmune glomerulonephritis (GN) and is one of the most common and severe complications of SLE with high mortality because of the risk of end-stage renal and cardiovascular diseases [6]. To study the pathophysiology of autoimmune GN, we developed a mouse model using the congenic B6.MRL-(*D1Mit202*–*D1Mit403*) (B6.MRLc1) strain carrying the telomeric region of chromosome 1 of SLE-prone MRL/MpJ mice on a C57BL/6 background [7]. In particular, evidence indicates that overexpression of Fc receptor, IgG, low affinity III (*Fcgr3*) and interferon-activated gene 202 (*Ifi202*) is associated with local inflammation of the glomerulus of B6.MRLc1 mice [8], [9]. Moreover, polymorphisms in the human homolog of *Fcgr3* and overexpression of *Ifi202* may predict the risk of developing SLE [10], [11].

In addition to local inflammatory processes, a recent study indicates that podocyte injury is a crucial event in the pathogenesis of autoimmune GN in lupus nephritis and IgA nephropathy [12], [13]. Podocytes are terminally differentiated glomerular epithelial cells, and their foot processes regulate the glomerular filtration barrier [14]. Podocyte injuries in SLE-prone mice are characterized by podocyte foot-process effacement, an elevated urinary albumin/creatinine ratio (uACR), and impaired localization and decreased expression of mRNAs encoding podocyte proteins [15]. Further, altered function of cytoskeletal molecules, particularly actin, crucially contributes to the progression of podocyte injury [16]. Moreover, transgenic expression of miR-193a in mice induces focal segmental glomerulosclerosis (FSGS) with downregulation of WT1, and miR-193a is overexpressed in the podocytes of patients with FSGS [17]. We previously showed that the expression level of miR-146a was significantly higher in the kidneys of B6.MRLc1 mice than in those of the C57BL/6 strain [18]. However, the increased level of miR-146a more closely correlates with the development of tubulointerstitial lesions, and the identities of miRNAs associated with glomerular damage of B6.MRLc1 mice are unknown.

In this study, we found that miR-26a expression significantly decreased in the glomerulus of B6.MRLc1 mice as well as in human patients with lupus nephritis and IgA nephropathy. Further, decreased miR-26a expression closely correlated with podocyte injuries characterized by decreased expression of molecules associated with podocyte differentiation and cytoskeletal structure. Further, miR-26a levels in urinary exosomes were significantly increased in B6.MRLc1 mice and in patients with lupus nephritis, indicating that altered miR-26a levels in glomerulus and urine may serve as a marker of injured podocytes in autoimmune GN.

## Methods

### Mouse Studies

Animal experimentation was approved by the Institutional Animal Care and Use Committee, which is convened at the Graduate School of Veterinary Medicine, Hokkaido University (approval No. 13-0032). The investigators adhered to the Guide for the Care and Use of Laboratory Animals of Hokkaido University, Graduate School of Veterinary Medicine (approved by the Association for the Assessment and Accreditation of Laboratory Animal Care International). Female B6.MRLc1 and C57BL/6 mice were maintained under specific pathogen-free conditions. C57BL/6 (9 months of age) served as healthy controls, and the B6.MRLc1 mice were divided into early and late stages (9 and 12–14 months of age, respectively) of GN [7]–[9], [18], [19]. After euthanasia by cutting the vena cava under deep anesthesia (Avertin; 2,2,2-tribromoethanol dissolved in 2-methyl-2-butanol, 1,000 mg/kg, administered intraperitoneally), the glomeruli were isolated using a magnetic bead perfusion method [20]. Part of each kidney was fixed in 4% paraformaldehyde (PFA) for histopathological analysis. Urine was centrifuged at 21,000×*g* for 10 min, and the supernatant was used for all analyses. The uACR was measured using Albuwell and The Creatinine Companion (Exocell; Philadelphia, PA, USA). For in situ hybridization or transmission electron microscopy (TEM), 4% PFA containing 2% sucrose or 2.5% glutaraldehyde was perfused, respectively.

### Human Studies

The Ethics Committee of Kochi University approved the study of human samples (approval No. ERB-100628). Written informed consent from the donor, the next of kin, or the person exercising parental authority for minors was obtained for the use of this sample in research. Human samples analyzed in this study are listed in Tables S1 and S2. Normal kidney tissues from autopsied humans without renal disease were obtained from KAC Inc. (Tokyo, Japan). These human patients died from causes other than kidney disease and showed no renal dysfunction in either blood biochemistry or urinalysis. Biopsies of kidney tissues containing normal glomeruli and those from patients with lupus nephritis were obtained from patients at Kochi University. The kidneys were fixed using 10% neutral buffered formalin or 4% PFA and were embedded in paraffin. Urine was collected from healthy human volunteers from Hokkaido University or patients with lupus nephritis at Kochi University. For LMD, deparaffinized sections were stained with toluidine blue. LMD was performed using a MicroBeam Rel.4.2 (Carl Zeiss; Oberkochen, Germany) on 50 glomeruli and tubulointerstitium sections with an area equal to that of the total area from which samples were collected.

### miRNA Expression Profiling of Isolated Glomeruli

For next-generation RNA sequencing, samples of total RNA isolated from the glomeruli from C57BL/6 mice (9 months of age, *n* = 4) were pooled. The concentration and purity of the RNA were determined using a Bioanalyzer (Agilent; Santa Clara, CA, USA). Libraries were prepared using a TruSeq Small RNA Library Preparation Kit (Illumina; San Diego, CA, USA) according to the manufacturer’s protocols and sequenced using 50-base reads acquired with a HiSeq 2000 platform.

For microarray analysis, total RNA extracted from the glomeruli of C57BL/6 (9 months of age, *n* = 3), B6.MRLc1 (9 months of age, *n* = 3), and B6.MRLc1 (14 months of age, *n* = 3) mice was labeled using a 3D-Gene miRNA labeling kit (Toray Industries; Kamakura, Japan). Labeled RNA was hybridized to 3D-Gene mouse miRNA Oligo chips (Toray Industries). After stringent washes, fluorescence intensities were determined using a 3D-Gene Scanner (Toray Industries) and analyzed using 3D-Gene Extraction software (Toray Industries). This Minimum Information About a Microarray Experiment-compliant dataset was deposited in the United States National Center for Biotechnology Information Gene Expression Omnibus and is accessible through GEO Series accession number GSE51209 (http://www.ncbi.nlm.nih.gov/geo/query/acc.cgi?token=avmbeykoffsdjkx&acc=GSE51209).

### In Situ Hybridization

We used a modified version of our published protocol to perform in situ hybridization analyses [18]. After perfusing kidney sections with 4% PFA for 2 h, the PFA was replaced with 0.01 M phosphate-buffered saline (PBS) containing 30% sucrose. The sections were incubated overnight and then embedded using optimal cutting temperature compound (Tissue-Tek O.C.T. Compound, Sakura Finetechnical Co. Ltd., Tokyo, Japan). Five-micrometer-thick cryosections were prepared and fixed with 4% PFA for 10 min. After washing with PBS, the sections were soaked in acetylation solution (1.3% triethanolamine, 0.175% concentrated HCl, 0.25% acetic anhydrate) for 10 min. After washing with PBS, the sections were digested with 10 µg/µl proteinase K for 45 min at room temperature. The sections were fixed with 4% PFA for 5 min, washed with water, and dried. Sections were incubated with prehybridization buffer containing 40% deionized formamide, 10 mM Tris-HCl (pH 7.4), 1× Denhardt’s solution (Sigma-Aldrich, St. Louis, MO, USA), 10% dextran sulfate, 600 mM NaCl, and 1 mM EDTA (pH 7.4) for 2 h at 50°C. Hybridization was performed overnight at 50°C in prehybridization buffer containing 3′-digoxigenin-labeled miRCURY LNA microRNA Detection Probes (100 nM; Exiqon; Vedbaek, Denmark). RNAs with scrambled sequences (100 nM, Exiqon) served as the negative control. Sections were washed with 5× standard-saline citrate (SSC) for 10 min at 50°C, 0.2× SSC for 1.5 h at 50°C, and in 100 mM Tris-HCl (pH 7.5) and 150 mM NaCl. The sections were incubated overnight with 0.2% polyclonal sheep anti-digoxigenin Fab fragments conjugated to alkaline phosphatase (1∶2000, Nucleic Acid Detection Kit; Roche Diagnostics, GmbH, Mannheim, Germany) at 4°C. After overnight incubation in the dark at room temperature, the signal was detected with nitroblue tetrazolium and 5-bromo-4-chloro-3-indolyl phosphate (Roche Diagnostics) in 100 mM Tris-HCl (pH 9.5), 100 mM NaCl, and 50 mM MgCl_2_.

### Histopathological Analysis

Paraffin-embedded kidney sections were stained with periodic acid Schiff (PAS) and periodic acid methenamine silver (PAM) reagents for histopathological analysis. Digital images of five glomeruli per kidney were prepared for histometric analysis. The glomerular area and the number of nuclei per glomerulus in PAS-stained sections were determined and analyzed using Photoshop CS4 (Adobe; Mountain View, CA, USA) as described [21]. The PAS-stained area per total area of glomerulus was measured as an index of glomerular sclerosis using ImageJ (National Institutes of Health (NIH); Bethesda, MD, USA). Sections analyzed using immunohistochemistry (Table S3) were evaluated for the number of WT1-positive nuclei per glomerular area using Photoshop CS4 (Adobe), and the signals detected in areas positive for podocin, NMIIA, synaptopodin, or vimentin were measured using ImageJ (NIH). The TEM specimens were postfixed with OsO_4_ and embedded in Quetol 812 (Nisshin EM; Tokyo, Japan), and ultrathin sections were stained with uranyl acetate and lead citrate.

### Cell Culture and Analysis of Cell Proliferation and Differentiation

The mouse M-1 cortical collecting-duct cell line (Dainippon Sumitomo Pharma, Osaka, Japan) and the MES13 mesangial cell line (American Type Culture Collection, Manassas, VA, USA) were maintained in DMEM/F12 medium containing 10% fetal bovine serum (FBS), 1× penicillin/streptomycin (PS; Life Technologies; Carlsbad, CA, USA), and 5 µM dexamethasone and in RPMI 1640 medium containing 10% FBS and PS, respectively. The immortalized mouse podocyte cell line (kindly provided from Dr. Jeffrey B Kopp, NIH, MA, USA) were maintained in RPMI 1640 medium containing 10% FBS and 1× PS (Life Technologies) (complete medium) at 33°C with recombinant mouse interferon-γ (10 U/ml; Cell Sciences, Canton, MA, USA) as described [22]. Differentiation was induced in the absence of interferon-γ by raising the temperature to 37°C.

LPS or PAN (Wako Pure Chemical; Osaka, Japan) was added to complete medium on day 12 after differentiation of mouse podocytes. Cell viability was measured using the CellTiter96 Non-Radioactive Cell Proliferation Assay (Promega; Fitchburg, WI, USA). To analyze cell morphology, LPS- or PAN-stimulated podocytes were fixed using 4% PFA and stained with fluorescein isothiocyanate-conjugated phalloidin (Life Technologies) and Hoechst 33342 (Dojindo; Kumamoto, Japan).

### Silencing of miR-26a Expression In Vitro

miR-26a expression was transiently suppressed using Anti-miR miRNA Inhibitors (Life Technologies). Immortalized mouse podocytes were cultured at 37°C without antibiotics 24 h before they were transfected. On day 3 or 11, the cells were trypsinized, and the medium was changed to a mixture of Lipofectamine 2000 and Opti-MEM (Life Technologies) containing the Anti-miR negative control or the miR-26a miRNA inhibitor (40 pmol). The cells were analyzed after culture for 24 h.

### Immunoblotting

Soluble proteins were extracted using RIPA lysis buffer (Santa Cruz Biotechnology; Dallas, TX, USA), and immunoblotting was performed using the NuPAGE electrophoresis system (Life Technologies) with the antibodies listed in Table S3. Immune complexes were detected using Typhoon Variable-Mode Imagers (GE Healthcare; Little Chalfont, UK). The intensity of each band was quantified using Image J (NIH).

### Exosome Isolation

Exosome fractions were isolated as described previously [23]. Urine samples or culture media were vigorously vortexed and then centrifuged at 21,000×*g* for 15 min to pellet cells, large membrane fragments, and other debris. The supernatant was then centrifuged at 200,000×*g* for 60 min to pellet exosomes.

### RNA Analysis

Total RNA from glomeruli, LMD samples, cultured cells, and exosome fractions was isolated using an miRNeasy Mini Kit (Qiagen; Venlo, Netherlands). Culture medium was collected and centrifuged at 21,000×*g* for 10 min, and total supernatant RNA was isolated using a 3-fold volume of Trizol LS (Life Technologies). To determine mRNA levels in glomeruli and cultured cells, total RNA was used as template to synthesize cDNA using reverse transcriptase (RT). Quantitative PCR analysis was performed using Brilliant II SYBR Master Mixes (Agilent) and specific primers (Table S4) with an MX3000P system (Agilent). The specificity of each PCR reaction was confirmed using melting curve analysis. The expression data were normalized to the expression levels of *Actb* (tissues) or *Gapdh* (cells). miRNA levels were determined using a TaqMan MicroRNA RT Kit (Applied Biosystems; Foster City, CA, USA). Quantitative PCR analysis was performed using each miRNA-specific TaqMan primer and TaqMan Universal PCR Master Mix (Applied Biosystems) with an MX3000P system (Agilent). We determined the levels of U6 snRNA for data normalization.

### Statistical Analyses

Results are expressed as the mean ± standard error (SE). The Student *t* test was used to compare two groups (*P*<0.05). To compare all samples vs. the control sample, significance was evaluated using Dunnett’s test (*P*<0.05). Spearman's correlation test (*P*<0.05) was used to analyze the correlation between two parameters.

## Results

### Pathological Features of the Glomeruli of GN Mice

**Figure 1:**
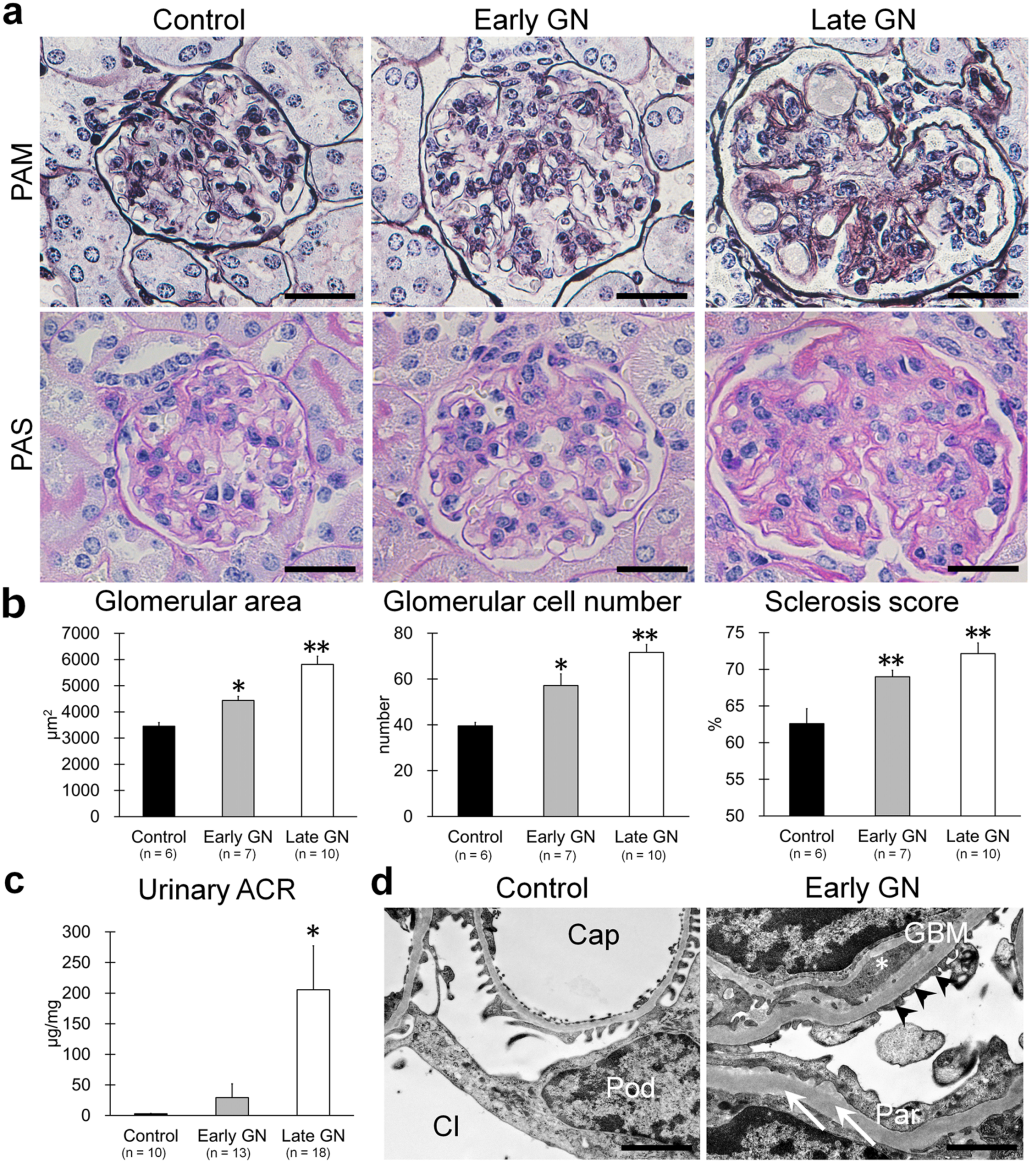
Glomerular pathology of GN-model mice. (**a**) Glomerular histology of C57BL/6 control mice and early- and late-stage B6.MRLc1 GN-model mice. PAM staining (upper panels), PAS staining (lower panels). Bars = 20 µm. GN mice developed membranous and proliferative lesions. (**b**) Histological scores of glomerular area, glomerular cell number, and sclerosis score in glomerulus. Values = mean ± SE. A significant difference from the control value is indicated by *(*P*<0.05) or **(*P*<0.01). (**c**) Level of uACR. Values = mean ± SE. A significant difference from the control value is indicated by *(*P*<0.05). (**d**) Early-stage glomerular ultrastructures of C57BL/6 control mice and B6.MRLc1 GN-model mice. Bars = 1 µm. Cap, capillary; Cl, capsular lumen; Pod, podocyte; GBM, glomerular basement membrane; Par, glomerular parietal epithelial cells. Arrowheads, podocyte foot process effacement. Asterisk, immune-complex deposition. Arrows, Bowman’s capsule.

We first analyzed glomerular pathology in B6.MRLc1 GN-model mice (GN mice) during early (9 months of age) and late (12–14 months of age) stages of disease (Figure 1). GN mice developed membranous and proliferative glomerular lesions during disease progression (Figure 1a). The indices for glomerular damage, such as the glomerular area, glomerular cell numbers, and sclerosis score were significantly higher in GN mice compared with C57BL/6 controls (Figure 1b). The uACR of GN mice increased early (2.9 µg/ml, control; 29.2 µg/ml, GN mice) and significantly during the late stage (Figure 1c). Early-stage GN mice exhibited increased double-contoured glomerular basement membranes and immune-complex deposition with podocyte foot-process effacement (Figure 1d).

### Identification of miRNAs Associated with GN Progression in Mice

We analyzed absolute miRNA expression levels in isolated glomeruli from healthy C57BL/6 mice by next-generation RNA sequencing (Table S5). The 10 miRNAs expressed at the highest levels were listed in table 1, and miR-26a was the most abundantly expressed miRNA in the glomerulus.

**Table 1:** miRNAs most abundantly expressed in the mouse glomerulus.

Rank	Name	miRNA ID	Read number	Normalized value
1	miR-26a-5p	MI0000573, MI0000706	470926	18.8
2	miR-126-5p	MI0000153	422785	18.6
3	miR-27b-3p	MI0000142	316117	18.2
4	miR-22-3p	MI0000570	284048	18.1
5	miR-10a-5p	MI0000685	165375	17.3
6	let-7f-5p	MI0000563	151579	17.2
7	miR-10b-5p	MI0000221	140437	17.1
8	miR-192-5p	MI0000551	106205	16.6
9	miR-191-5p	MI0000233	93518	16.5
10	let-7c-5p	MI0000560, MI0000559	91058	16.4

Next-generation RNA sequencing. Total RNA was collected from four mice and pooled as one sample. miRBase was used as the source of miRNA names and IDs. The 10 miRNAs expressed at the highest levels are listed.

**Figure 2:**
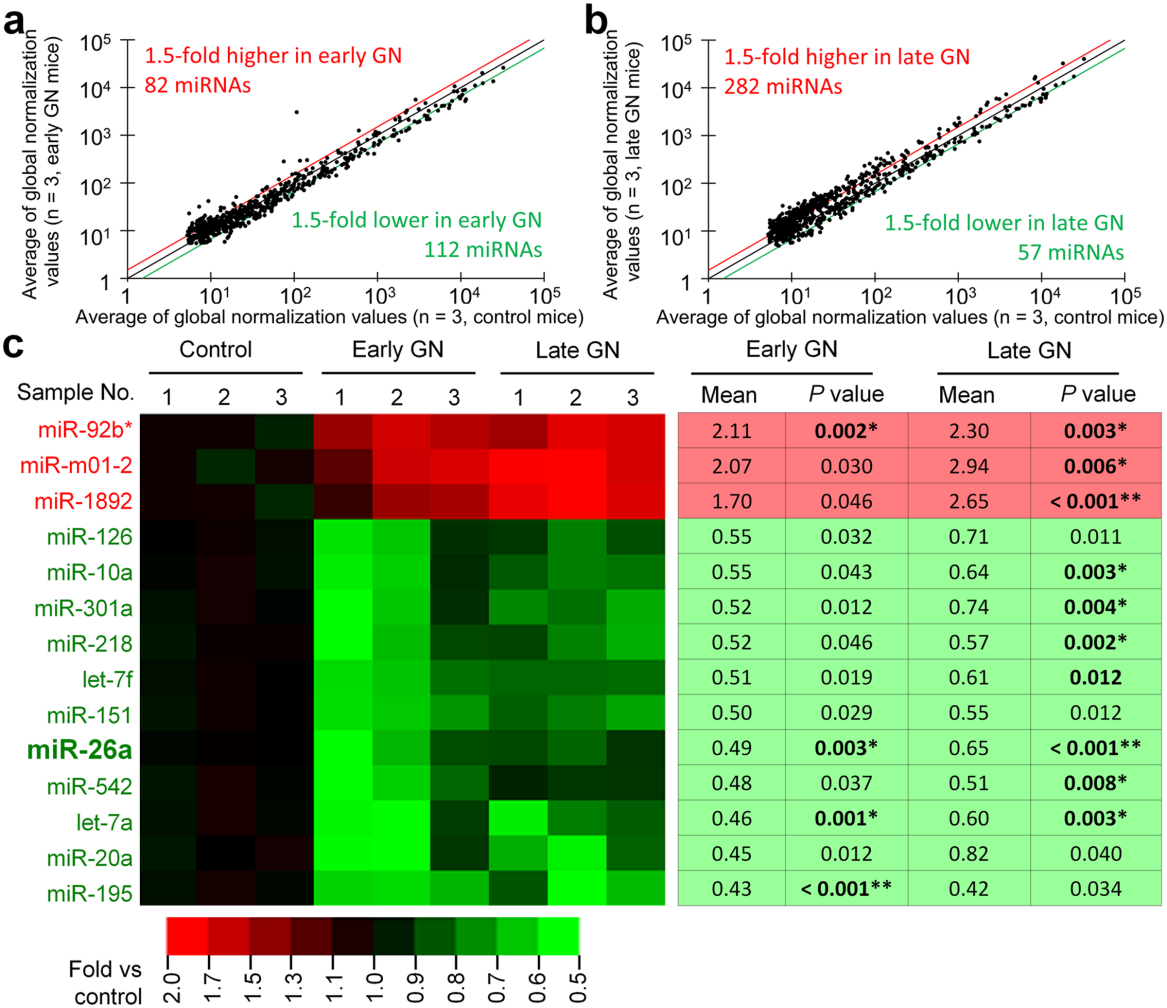
Altered miRNA expression in the glomerulus of GN-model mice. (**a**) Scatter plot of glomerular miRNA expression detected using microarray analysis to compare C57BL/6 control with early-stage B6.MRLc1 GN-model mice. (**b**) Scatter plot of glomerular miRNA microarray expression data to compare C57BL/6 control with late-stage B6.MRLc1 GN-model mice. Values = average of global normalized values. Red, black, and green lines indicate the boundaries of miRNAs expressed at 1.5-fold higher, equal, and 1.5-fold lower levels, respectively, in GN compared with control mice. (**c**) Heat map of glomerular miRNA expression determined using microarray analysis to compare C57BL/6 control mice and early- and late-stage B6.MRLc1 GN-model mice. Only those miRNAs with significant differential expression (*P*<0.05) in early and late GN compared with controls are listed. Red and green indicate increased and decreased expression, respectively. Heat-map scale bars indicate the relative changes. The relative expression and *P* values of each miRNA are shown in the table on the right. A significant difference from the control is indicated by *(*P*<0.005) or **(*P*<0.001).

Microarray analysis targeting 1,135 miRNAs showed that 82 and 112 miRNAs were expressed at 1.5-fold higher or lower levels, respectively, during the early stage compared with controls (Figure 2a); 282 and 57 were expressed at 1.5-fold higher or lower levels, respectively, during the late stage compared with controls (Figure 2b). We identified 3 and 11 miRNAs that were expressed at significantly (*P*<0.05) higher or lower levels, respectively, in the glomerulus of GN mice than in the controls during early and late stages (Figure 2c). Importantly, miR-26a was the most abundantly expressed miRNA in the glomerulus of controls (Table 1), and glomerular miR-26a expression was significantly lower in GN mice than in the controls during early and late stages (*P*<0.005). Further, it was reported that miR-26a is expressed by mouse and human podocyte cell lines [24]. Therefore, we focused on miR-26a in subsequent experiments.

### Glomerular miR-26a Expression in Mice and Humans

**Figure 3:**
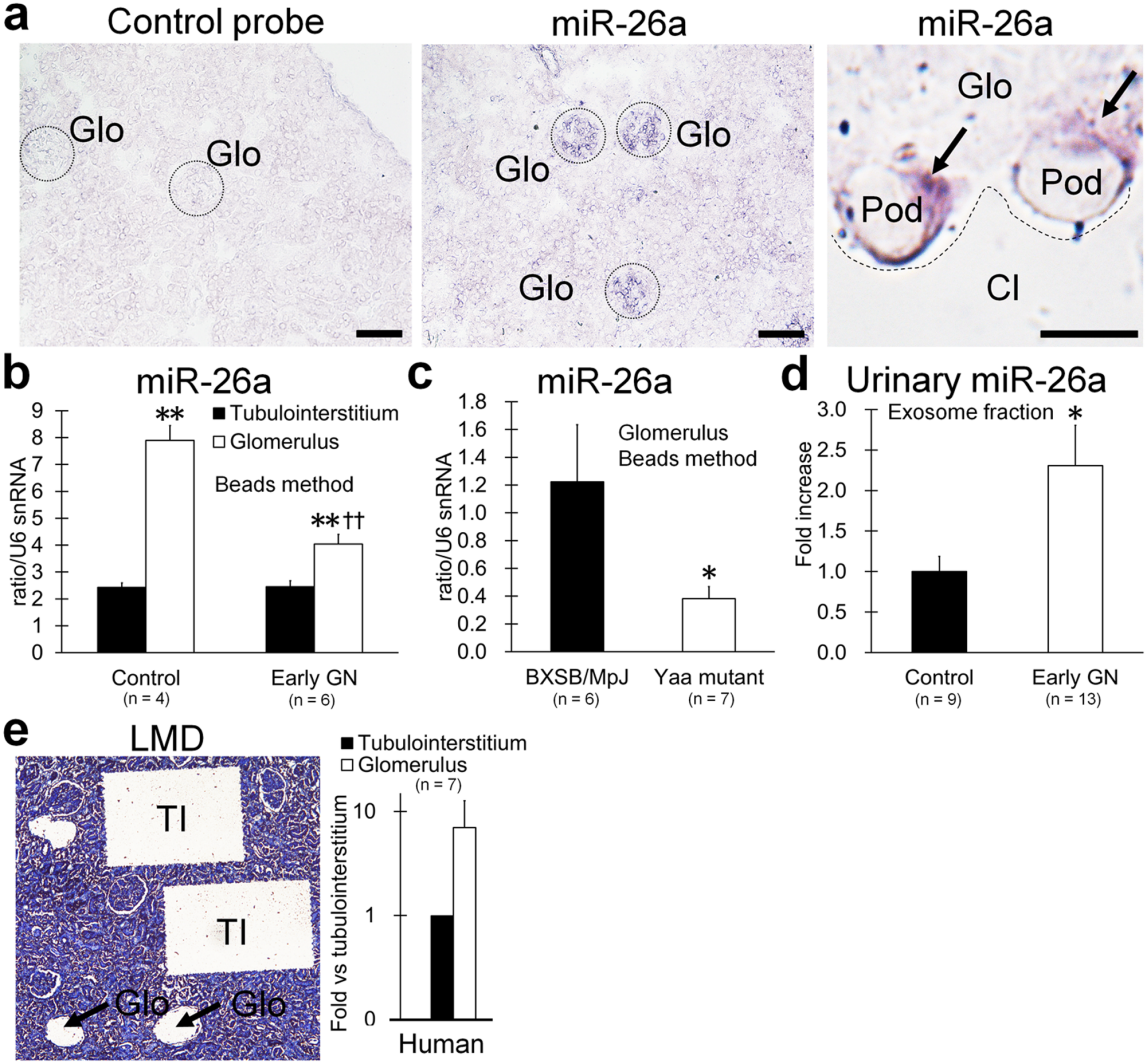
miR-26a expression in the glomerulus and tubulointerstitium of GN-model mice and healthy humans. (**a**) Localization of miR-26a expression using *in situ* hybridization with negative control and LNA probes for miR-26a. Female C57BL/6 mice at 9 months of age. Glo, glomerulus; Pod, podocyte; Cl, capsular lumen. Black arrows, miR-26a-positive signals in podocyte cytoplasm. Bars = 50 µm (low maginification) and 5 µm (high maginification). (**b**) miR-26a expression in isolated glomeruli and tubulointerstitium of C57BL/6 control mice and early-stage B6.MRLc1 GN-model mice. Bead perfusion method. PCR analysis. Values = mean ± SE. A significant difference from the tubulointerstitium is indicated by **(*P*<0.01). A significant difference from the control is indicated by ††(*P*<0.01). (**c**) miR-26a expression in isolated glomeruli of BXSB/MpJ control mice (males, 4 months of age) and BXSB/MpJ*^Yaa^* lupus nephritis-model mice (males, 4 months of age). Bead perfusion method. PCR analysis. Values = mean ± SE. A significant difference from control is indicated by *(*P*<0.05). (**d**) miR-26a levels in urinary exosomes from C57BL/6 control mice and early-stage B6.MRLc1 GN-model mice. Values represent the proportional increases relative to controls. Values = mean ± SE. A significant difference from the control is indicated by *(*P*<0.05). (**e**) miR-26a expression in isolated glomeruli and tubulointerstitium of a healthy human. LMD method. PCR analysis. Values are expressed as the proportional increase compared with the tubulointerstitium. Values = mean ± SE. Glo, glomerulus. TI, tubulointerstitium.

*In situ* hybridization analysis showed that miR-26a expression in control mouse kidney was focally localized in glomeruli, particularly to podocytes (Figure 3a). Further, we compared miR-26a levels for the isolated tubulointerstitium and glomeruli of control and early-stage GN mice by TaqMan PCR analysis (Figure 3b). miR-26a was predominantly expressed in glomeruli compared with the tubulointerstitium, and glomerular miR-26a expression was significantly lower in GN mice than in the controls. Similarly, glomerular miR-26a expression was significantly lower in BXSB/MpJ*^Yaa^* lupus nephritis-model mice than in the controls (Figure 3c). Interestingly, the miR-26a level in urinary exosomes was significantly higher in GN mice compared with controls (Figure 3d).

In healthy humans, laser microdissection (LMD) analysis also revealed that miR-26a expression in the glomerulus was higher than that in the tubulointerstitium (Figure 3e).

### Correlation between Podocyte Injuries and Glomerular miR-26a Levels in GN Mice

**Figure 4:**
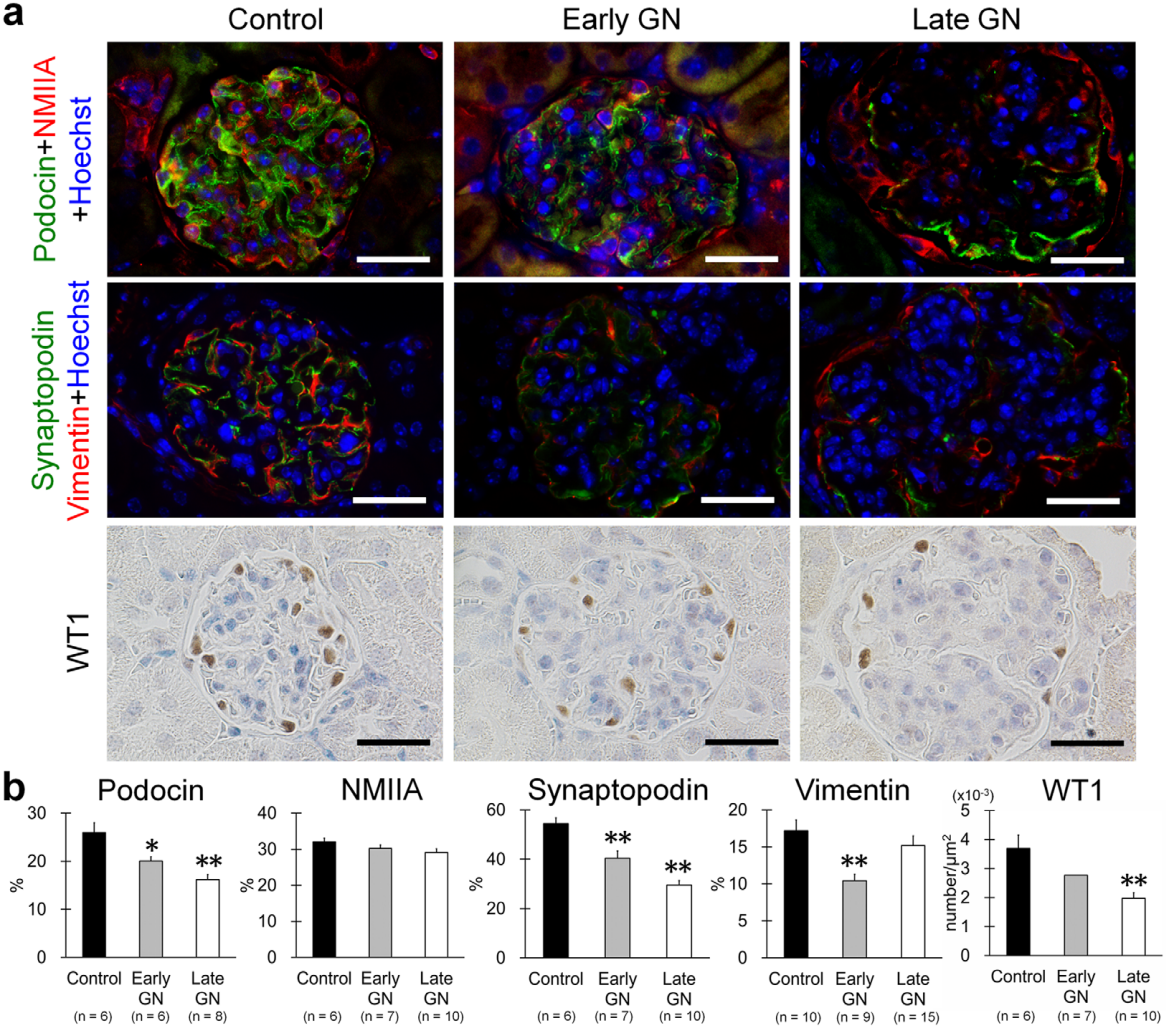
Podocyte injuries in GN-model mice. (**a**) Glomerular localization of podocin, NMIIA, synaptopodin, vimentin, and WT1 in C57BL/6 control mice and early- and late-stage B6.MRLc1 GN-model mice. (**b**) The glomerular area showing podocin, NMIIA, synaptopodin, vimentin, and WT1-positive cells. Values = mean ± SE. A significant difference from the control is indicated by *(*P*<0.05) or **(*P*<0.01).

Figure 4 shows the histology of podocyte injuries in GN mice. Podocyte proteins (podocin, non-muscle myosin IIA (NMIIA), synaptopodin, vimentin, and WT1) were expressed at lower levels in GN mice with progressive disease (Figure 4a). Significant differences during the early stage of GN were noted between the results of histometry analysis for control and GN mice in the podocin-, synaptopodin-, and vimentin-positive areas and in the number of cells showing late WT1 expression (Figure 4b).

**Figure 5:**
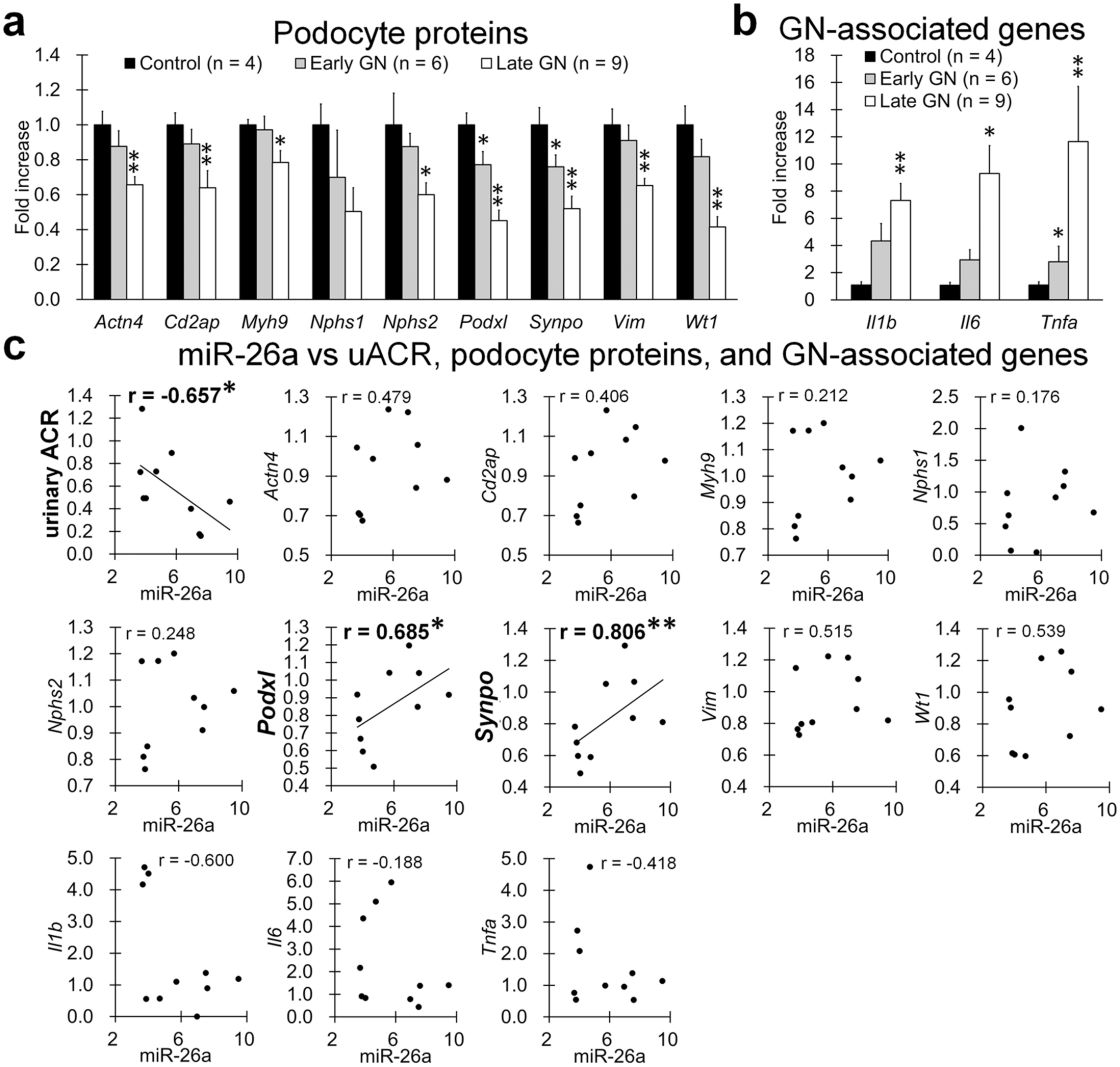
Correlation between glomerular miR-26a expression and indices for podocyte injuries in GN-model mice. (**a** and **b**) Expression of mRNAs encoding podocyte proteins and GN-associated genes in isolated glomeruli. Bead perfusion method. Values are expressed as the proportional increase relative to control glomeruli. Values = means ± SE. A significant difference from control is indicated by *(*P*<0.05) or **(*P*<0.01). (**c**) Correlation between glomerular miR-26a expression, uACR, and glomerular mRNA expression of podocyte proteins and GN-associated genes (*n* = 10, mice 9 months of age). Expression values are expressed as the fold-increase relative to those of the C57BL/6 control. Spearman's correlation test was used to analyze the correlation between two parameters, and a significant correlation is indicated by *(*P*<0.05) or **(*P*<0.01).

The levels of mRNAs encoding podocyte proteins in isolated glomeruli were significantly lower in GN mice than in controls (Figure 5a). There were significant early-stage differences in *Podxl* and *Synpo* levels and in late-stage expression of *Actn4*, *Cd2ap*, *Myh9*, *Npsh2*, *Vim*, and *Wt1*. Their decreased expression correlated significantly with glomerular dysfunction from early GN (Table S2). The levels of mRNAs encoding GN-associated proteins [25], [26] were higher in GN mice than in controls; there were significant early differences in *Tnfa* and in late expression of *Il1b* and *Il6* (Figure 5b). Figure 5c shows the correlation between early miR-26a expression and clinical parameters or glomerular mRNA expression. Glomerular miR-26a expression correlated negatively with uACR and positively with *Podxl* and *Synpo*.

### miR-26a Expression in Injured Mouse Podocytes

**Figure 6:**
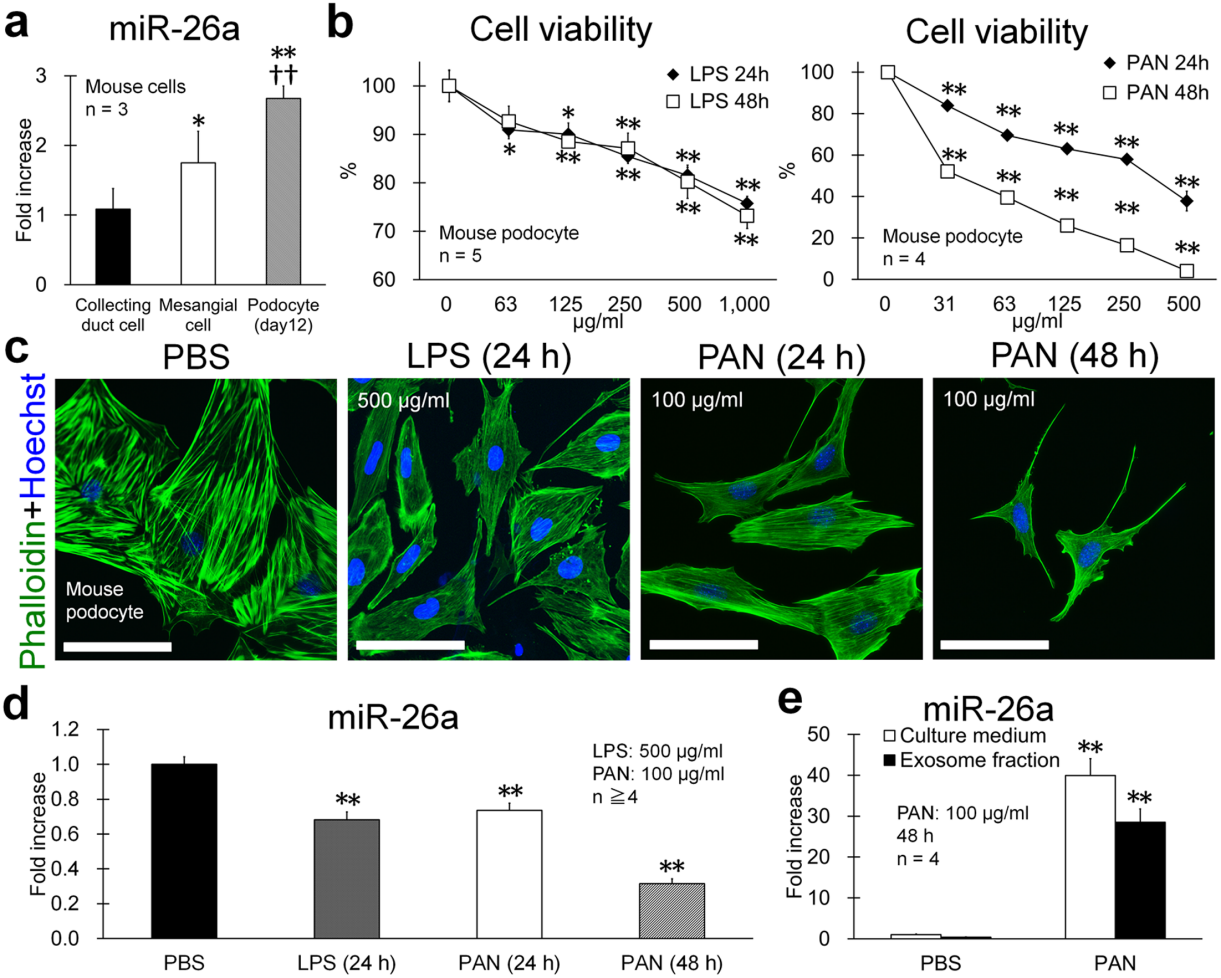
miR-26a expression in injured mouse podocytes. (**a**) miR-26a expression in mouse renal cells. Values are expressed as the proportional increase relative to that of mouse collecting-duct cells. Values = means ± SE. A significant difference from collecting-duct cells is indicated by *(*P*<0.05) or **(*P*<0.01). A significant difference from mesangial cells is indicated by ††(*P*<0.01). (**b**) Viability of LPS- or PAN-treated mouse podocytes. Values = mean ± SE. Significant difference from untreated cells is indicated by *(*P*<0.05) or **(*P*<0.01). (**c**) Mouse podocytes treated with PBS, LPS, or PAN on day 12 after differentiation. LPS- or PAN-treated podocytes show cytoskeletal changes characterized by decreased cell size, altered shape, and weak actin fibers. Detection of actin and nuclei using phalloidin and Hoechst 33342, respectively. Bars = 20 µm. (**d**) miR-26a expression in PBS-, LPS-, or PAN-treated mouse podocytes. Values are expressed as the proportional increase relative to that of the PBS control. Values = mean ± SE. A significant difference from the PBS control is indicated by **(*P*<0.01). (**e**) miR-26a levels in culture medium and exosomes collected from culture medium after PAN treatment for 48 h. Mouse podocytes. Values are expressed as the proportional increase relative to that of the PBS control. Values = mean ± SE. Significant difference from the PBS control is indicated by **(*P*<0.01).

Mouse podocytes conditionally immortalized by a temperature-sensitive SV40 large T-antigen exhibited undifferentiated and differentiated morphologies at 33°C and 37°C, respectively [22]. The expression of miR-26-a was significantly higher in differentiated mouse podocytes among cell lines derived from mouse kidneys on day 12 after culture at 37°C (Figure 6a). Lipopolysaccharide (LPS) and puromycin aminonucleoside (PAN) injure podocytes [27], and in the present study, reduced the viability of mouse podocytes (Figure 6b) and altered their morphology (more fusiform shape and weak actin fibers from 24 h) (Figure 6c). miR-26a expression started to decrease significantly 24 h after treatment with LPS or PAN (Figure 6d). The levels of miR-26a in culture medium and in culture-fluid exosomes increased significantly 48 h after adding PAN (Figure 6e).

### Effects of Silencing miRNA-26a Expression on the Phenotype of Mouse Podocytes

**Figure 7:**
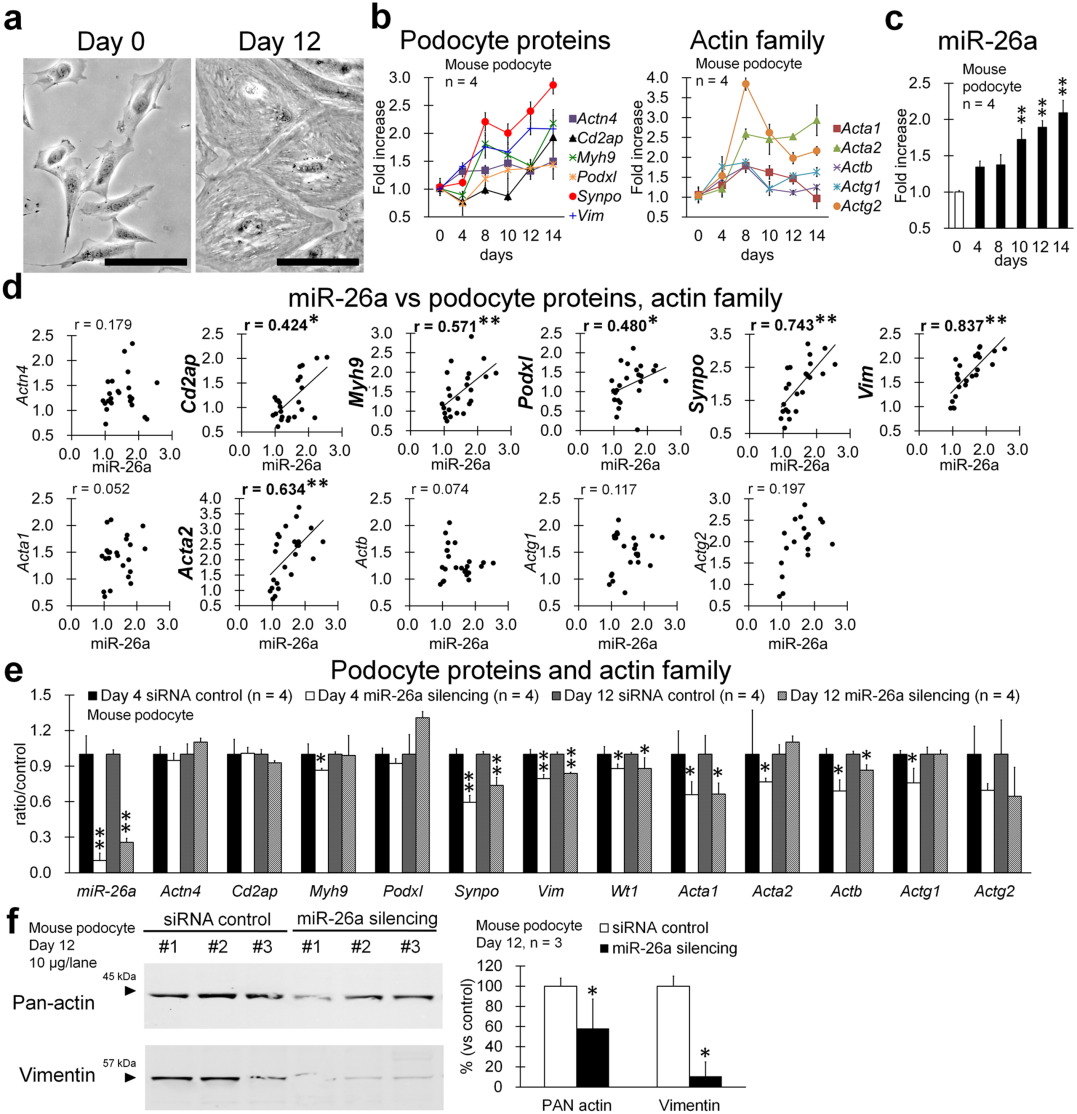
Correlation between miR-26a expression and podocyte differentiation. (**a**) Differentiation of immortalized mouse podocytes. Podocytes at days 0 and 12 after differentiation. Bars = 5 µm. (**b**) Time-course of mRNA expression of podocyte and actin family proteins in mouse podocytes. Values are expressed as the proportional increase relative to that of day 0. Values = mean ± SE. (**c**) miR-26a expression in mouse podocytes. Values are expressed as the proportional increase relative to that of day 0. Values = means ± SE. A significant difference from day 0 is indicated by **(*P*<0.01). (**d**) Correlation between expression of miR-26a and mRNAs encoding podocyte and actin family proteins. Samples were collected on days 0, 4, 8, 10, 12, and 14 (*n* = 3 for each time point). Values are expressed as the proportional increase relative to that of day 0. Spearman's correlation test was used to analyze the correlation between two parameters, and a significant correlation is indicated by *(*P*<0.05) or **(*P*<0.01). (**e**) Expression of miR-26a and mRNAs encoding podocyte and actin family member proteins in mouse podocytes after silencing miR-26a expression on days 4 and 12. Values are expressed as the proportional increase relative to the siRNA control. Values = mean ± SE. A significant difference from the siRNA control is indicated by *(*P*<0.05) or **(*P*<0.01). (**f**) Immunoblotting analysis of pan-actin and vimentin expression in mouse podocytes with transient silencing of miR-26a. Values = mean ± SE. A significant difference from the control is indicated by *(*P*<0.05).

The expression of mRNAs encoding podocyte proteins and miR-26a increased with podocyte differentiation (Figure 7a–c), and the expression of actin family members increased by day 4 after differentiation (Figure 7b). The level of miR-26a increased significantly after 10 days (Figure 7c). Further, miR-26a levels correlated significantly with those of *Cd2ap*, *Myh9*, *Podxl*, *Synpo*, *Vim*, and *Acta2* in mouse podocytes cultured for 0–14 days after differentiation (Figure 7d), and the level of miR-26a correlated most closely with that of *Vim*.

Based on these results, we next examined the effect of miR-26a silencing on the differentiation and cytoskeleton of mouse podocytes. Because expression of actin family genes increased by day 4 after differentiation (Figure 7b), we examined cells on days 4 (early differentiation) and 12 (fully differentiated). Silencing of miR-26a significantly decreased the expression of *Myh9*, *Synpo*, *Vim, Wt1*, *Acta1*, *Acta2*, *Actb*, and *Actg1* mRNAs by day 4 and of *Synpo*, *Vim*, *Wt1*, *Acta1*, and *Actb* mRNAs by day 12 (Figure 7e). Immunoblotting analysis demonstrated that pan-actin and vimentin levels were significantly reduced in mouse podocytes with transient knockdown of miR-26a on day 12 (Figure 7f).

### miR-26a Levels in the Glomerulus and Urine of Human Patients with Autoimmune GN

**Figure 8:**
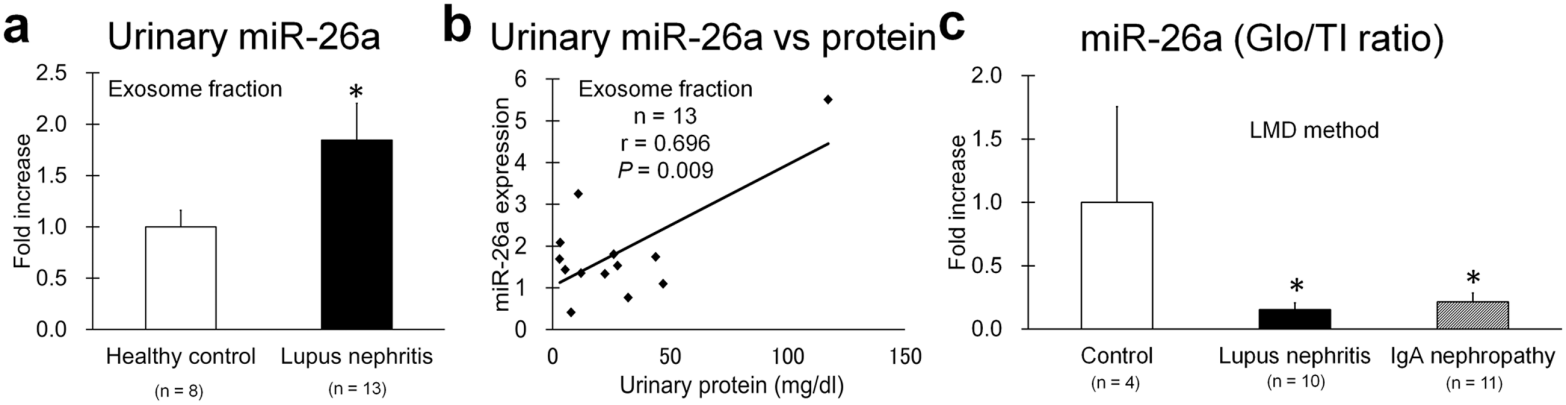
miR-26a levels in urine and glomerulus in patients with autoimmune GN. (**a**) miR-26a levels in urinary exosomes of from healthy controls and patients with lupus nephritis. Values represent the proportional increases relative to controls. Values = mean ± SE. A significant difference from the control is indicated by *(*P*<0.05). (**b**) Correlation between miR-26a levels in urinary exosomes and urinary protein levels in patients with lupus nephritis. Spearman's correlation test was used to analyze the correlation between two parameters. (**c**) The ratio of miR-26a expression in isolated glomeruli (Glo) to tubulointerstitium (TI) of healthy controls to that of patients with lupus nephritis or IgA nephropathy. LMD method. PCR analysis. Values are expressed as the proportional increase relative to the control. Values = mean ± SE. A significant difference from the control is indicated by *(*P*<0.05).

The miR-26a levels in urinary exosomes were increased in patients with lupus nephritis compared with healthy controls and correlated positively with urinary protein levels (Figure 8a and b). Further, the ratio of miR-26a expression in glomerulus vs. tubulointerstitium was significantly lower in patients with lupus nephritis as well as those with IgA nephropathy compared with healthy controls (Figure 8c, Table S4).

## Discussion

We show here that glomerular miR-26a expression decreased significantly in autoimmune GN mice and patients with lupus nephritis and IgA nephropathy and that miR-26a was most abundantly expressed in the normal mouse glomerulus and was expressed by podocytes. Moreover, miR-26a is specifically expressed at high levels in mouse glomeruli and in human and mouse podocyte cell lines [24], [28]. Therefore, we conclude that podocytes were the primary site of expression of miR-26a in mouse kidneys.

During early GN, miR-26a expression in the glomerulus correlated significantly with elevated uACR values and positively with decreased expression of *Podxl* and *Synpo*. Elevated uACR and decreased expression of podocyte proteins are useful indices for assessing podocyte injuries in GN models [15]. Further, glomerular inflammation is a fundamental process in GN progression [25], [26]. We show here that the glomerular expression of *Il6* and *Tnfa* was relatively high and correlated significantly with elevated uACR in GN mice (Table S6). However, there was no significant correlation between the levels of glomerular miR-26a expression and those of GN-associated inflammatory mediators such as *Il1b*, *Il6*, and *Tnfa*. Therefore, we conclude that altered miR-26a expression was more closely linked to the progression of podocyte injury than with glomerular inflammation in GN mice.

In immortalized mouse podocytes, treatment with LPS and PAN, which injure podocytes [27], decreased the expression of miR-26a by podocytes. Moreover, LPS- or PAN-treated podocytes showed cytoskeletal changes characterized by decreased cell size, altered shape, and weak actin fibers, indicating that decreased miR-26a expression in podocytes correlated closely with perturbed cytoskeletal structure. Further, miR-26a levels in mouse podocytes increased with differentiation characterized by increased cell size, presence of cytoskeletal fibers, and expression of genes encoding podocyte proteins and actin family members.

Our working hypothesis that miR-26a plays an important role in maintaining podocyte homeostasis was further supported by our findings that silencing miR-26a expression in immortalized mouse podocytes decreased the expression of genes encoding podocyte proteins. In particular, mRNA and protein levels of vimentin and actin family members significantly decreased when miR-26a expression was inhibited in mouse podocytes. The intermediate filament vimentin is a component of the podocyte cytoskeleton, a marker of podocyte maturation, and its expression is decreased significantly in injured podocytes associated with diabetic nephropathy [29].

Altered actin expression and function are critical for the progression of podocyte injury, and the significantly decreased expression of the mRNA encoding synaptopodin in mouse podocytes when miR-26a expression is silenced indicates disruption of the formation and dynamic reorganization of the actin cytoskeleton [30]. The mechanism that regulates the expression of podocyte proteins and actin family members by miR-26a is unknown; however, some miRNAs directly alter the transcriptional activities of target genes [31], [32]. For example, miR-26a mediates the differentiation of smooth and skeletal muscles with highly organized actin cytoskeletons [33], [34]. Interestingly, miR-26a expression is up-regulated in smooth muscle cells in desmin-null mice, indicating the transcriptional interactions between miR-26a and a gene encoding an intermediate filament [33]. Therefore, we propose that miR-26a regulates the differentiation and maintenance of the cytoskeleton of podocytes by regulating the balance of expression of podocyte proteins, actin family members, and intermediate filaments, and that decreased miR-26a expression correlates closely with podocyte injuries due to imbalanced expression of the genes encoding these proteins. On the other hand, the silencing of functional and cytoskeletal genes in podocytes is somewhat weakened by miR-26a silencing. These data might also indicate that there are other target genes of miR-26a in addition to the genes elucidated in this study.

miRNAs are released into the urine [3], [4], [23], [35], and several urinary miRNAs may serve as biomarkers for renal fibrosis, lupus nephritis, and diabetic nephropathy [5], [36], [37]. However, to link miRNA to pathogenesis, it is important to determine their expression in different species. For example, increased renal expression of miR-146a and the presence of miR146a in urine are associated with tubulointerstitial inflammation in SLE-prone mice [18], and miR-146a expression correlates with glomerular lesions in patients with lupus [38]. In this study, the relative level of miR-26a in the glomerulus of patients with lupus nephritis or IgA nephropathy was similar to that of GN mice. Further, in patients with lupus nephritis, miR-26a levels in exosomes were significantly higher compared with controls, similar to findings for GN mice. Moreover, our analysis using PAN-treated mouse podocytes demonstrates that increased miR-26a levels in exosomes correlated with those released by cells into the culture medium. Taken together, these data indicate that determining miR-26a levels in urinary exosomes may predict glomerular disease; this may be especially possible by using miR-26a expression as a direct biomarker indicating injury to a podocyte. To use miR-26a as a biomarker, a detailed experiment focusing on miR-26a expression in a human clinical subject would be required, with an evaluation of its level in urinary exosomes. Such an experiment could determine the effect of therapy by comparing the glomerular miR-26a expression in patients having baseline and repeated biopsies.

In conclusion, we show here that miR-26a was predominantly expressed in the glomeruli of mice as well as those of other species, including humans. Our data indicate that decreased expression of miR-26a closely correlates with the progression of podocyte injury.

## Supporting Information

Table S1 **Human samples for urinary miR-26a expression analysis.**
(DOCX)

Table S2 **Human samples for glomerular miR-26a expression analysis.**
(DOCX)

Table S3 **Antibodies used in this study.**
(DOCX)

Table S4 **Primers and probes used in this study.**
(DOCX)

Table S5 **Results of next-generation RNA sequensing.**
(DOCX)

Table S6 **Correlation between indices for podocyte injuries and urinary albumin/creatinine ratio in GN-model mice.**
(DOCX)
