# Supplementary material for: Decreased miR-26a Expression Correlates with the Progression of Podocyte Injury in Autoimmune Glomerulonephritis
Source: PLoS One. 2014 Oct 17;9(10):e110383. doi: 10.1371/journal.pone.0110383 (PMC4201534; doi:10.1371/journal.pone.0110383)
Supplement: Table S1 — Human samples for urinary miR-26a expression analysis. (DOCX) [file pone.0110383.s001.docx]

**Table S1. Human samples for urinary miR-26a expression analysis.**

| **No** | **Age** | **Sex** | **Group** | **Renal pathology** | **Application** |
| --- | --- | --- | --- | --- | --- |
| 1 | 28 | Male | Control | No pathological change | Urinary exosome |
| 2 | 24 | Male | Control | No pathological change | Urinary exosome |
| 3 | 34 | Male | Control | No pathological change | Urinary exosome |
| 4 | 26 | Male | Control | No pathological change | Urinary exosome |
| 5 | 22 | Male | Control | No pathological change | Urinary exosome |
| 6 | 31 | Male | Control | No pathological change | Urinary exosome |
| 7 | 31 | Female | Control | No pathological change | Urinary exosome |
| 8 | 24 | Female | Control | No pathological change | Urinary exosome |
| 9 | 70 | Female | Patient | Lupus nephritis | Urinary exosome |
| 10 | 36 | Male | Patient | Lupus nephritis | Urinary exosome |
| 11 | 52 | Female | Patient | Lupus nephritis | Urinary exosome |
| 12 | 64 | Female | Patient | Lupus nephritis | Urinary exosome |
| 13 | 31 | Female | Patient | Lupus nephritis | Urinary exosome |
| 14 | 24 | Female | Patient | Lupus nephritis | Urinary exosome |
| 15 | 71 | Female | Patient | Lupus nephritis | Urinary exosome |
| 16 | 49 | Female | Patient | Lupus nephritis | Urinary exosome |
| 17 | 66 | Female | Patient | Lupus nephritis | Urinary exosome |
| 18 | 70 | Female | Patient | Lupus nephritis | Urinary exosome |
| 19 | 16 | Female | Patient | Lupus nephritis | Urinary exosome |
| 20 | 32 | Female | Patient | Lupus nephritis | Urinary exosome |
| 21 | 41 | Female | Patient | Lupus nephritis | Urinary exosome |
