# Supplementary material for: Decreased miR-26a Expression Correlates with the Progression of Podocyte Injury in Autoimmune Glomerulonephritis
Source: PLoS One. 2014 Oct 17;9(10):e110383. doi: 10.1371/journal.pone.0110383 (PMC4201534; doi:10.1371/journal.pone.0110383)
Supplement: Table S2 — Human samples for glomerular miR-26a expression analysis. (DOCX) [file pone.0110383.s002.docx]

**Table S2. Human samples for glomerular miR-26a expression analysis.**

| **No** | **Age** | **Sex** | **Group** | **Renal pathology** | **Application** |
| --- | --- | --- | --- | --- | --- |
| 1 | 69 | Male | Control | No pathological change | Autopsy-LMD |
| 2 | 70 | Female | Control | No pathological change | Autopsy-LMD |
| 3 | 30 | Male | Control | Minor glomerular abnormalities | Biopsy-LMD |
| 4 | 52 | Female | Control | Minor glomerular abnormalities | Biopsy-LMD |
| 5 | 61 | Female | Patient | Lupus nephritis IV-S (A/C) | Biopsy-LMD |
| 6 | 58 | Female | Patient | Lupus nephritis III (A/C) | Biopsy-LMD |
| 7 | 25 | Female | Patient | Lupus nephritis IV-S (A/C) | Biopsy-LMD |
| 8 | 16 | Female | Patient | Lupus nephritis III (C) | Biopsy-LMD |
| 9 | 59 | Male | Patient | Lupus nephritis III (C) | Biopsy-LMD |
| 10 | 45 | Female | Patient | Lupus nephritis IV-G (A/C) | Biopsy-LMD |
| 11 | 22 | Female | Patient | Lupus nephritis IV-G (A/C) | Biopsy-LMD |
| 12 | 55 | Female | Patient | Lupus nephritis IV-G (A/C) | Biopsy-LMD |
| 13 | 20 | Female | Patient | Lupus nephritis IV-G (A/C) | Biopsy-LMD |
| 14 | 24 | Female | Patient | Lupus nephritis IV-G (A/C) | Biopsy-LMD |
| 15 | 35 | Male | Patient | IgA nephropathy I (A/C) | Biopsy-LMD |
| 16 | 52 | Female | Patient | IgA nephropathy I (A/C) | Biopsy-LMD |
| 17 | 17 | Male | Patient | IgA nephropathy I(A/C) | Biopsy-LMD |
| 18 | 32 | Female | Patient | IgA nephropathy I (A/C) | Biopsy-LMD |
| 19 | 32 | Female | Patient | IgA nephropathy I(A/C) | Biopsy-LMD |
| 20 | 52 | Male | Patient | IgA nephropathy I (A/C) | Biopsy-LMD |
| 21 | 21 | Female | Patient | IgA nephropathy I(C) | Biopsy-LMD |
| 22 | 55 | Male | Patient | IgA nephropathy II (A/C) | Biopsy-LMD |
| 23 | 60 | Male | Patient | IgA nephropathy II(A/C) | Biopsy-LMD |
| 24 | 66 | Male | Patient | IgA nephropathy III (C) | Biopsy-LMD |
| 25 | 61 | Female | Patient | IgA nephropathy I (C) | Biopsy-LMD |
| Histological grade. A: active lesions. C: chronic inactive lesions with scars. G: diffuse global proliferative lupus nephritis. The classification of glomerulonephritis (Am Soc Nephrol 15: 241–250, 2004, Am J Kidney Dis 29: 829-842, 1997). LMD: laser microdissection. | | | | | |
