# Supplementary material for: Decreased miR-26a Expression Correlates with the Progression of Podocyte Injury in Autoimmune Glomerulonephritis
Source: PLoS One. 2014 Oct 17;9(10):e110383. doi: 10.1371/journal.pone.0110383 (PMC4201534; doi:10.1371/journal.pone.0110383)
Supplement: Table S3 — Antibodies used in this study. (DOCX) [file pone.0110383.s003.docx]

**Table S3. Antibodies used in this study.**

| **Antigen** |  | **Primary antibody** |  | **Secondary antibody for IHC** |  | **Secondary antibody for IF** |  | **Secondary antibody for WB** |  | **Antigen retrieval for sections** |
| --- | --- | --- | --- | --- | --- | --- | --- | --- | --- | --- |
| **NMIIA** |  | Mouse monoclonal antibodies (No. ab55456, 1:200 (IF, IHC), 1:1000 (WB); Abcam, Cambridge, UK) |  | Simple Stain Mouse MAX-PO(M) (mouse stain kit, Nichirei) |  | Alexa Fluor labeled donkey anti-mouse IgG antibodies (1:500, Life Technologies) |  | **-** |  | Citrate buffer (pH 6.0) |
| **Pan-actin** |  | Rabbit polyclonal antibodies (No. 4968, 1:1000; Cell Signaling, MA, USA) |  | **-** |  | **-** |  | Alexa Fluor labeled donkey anti-rabbit IgG antibodies (1:5000, Life Technologies) |  | **-** |
| **Podocin** |  | Rabbit polyclonal antibodies (No. 29070, 1:800; IBL, Gunma, Japan) |  | Biotinylated goat anti-rabbit IgG antibodies (SABPO kit, Nichirei, Tokyo, Japan) |  | Alexa Fluor labeled donkey anti-rabbit IgG antibodies (1:500, Life Technologies, Carlsbad, CA, USA) |  | **-** |  | Citrate buffer (pH 6.0) |
| **Synaptopodin** |  | Mouse monoclonal antibodies (No. 10R-S125a, 1:50; Fitzgerald, MA, USA) |  | Simple Stain Mouse MAX-PO(M) (mouse stain kit, Nichirei) |  | Alexa Fluor labeled donkey anti-mouse IgG antibodies (1:500, Life Technologies) |  | **-** |  | Dako Target Retrieval Solution at pH 9 (DAKO, Glostrup, Denmark) |
| **Vimentin** |  | Rabbit monoclonal antibodies (No. 5741, 1:1000; Cell Signaling, MA, USA) |  | Biotinylated goat anti-rabbit IgG antibodies (SABPO kit, Nichirei) |  | Alexa Fluor labeled donkey anti-rabbit IgG antibodies (1:500, Life Technologies) |  | Alexa Fluor labeled donkey anti-rabbit IgG antibodies (1:5000, Life Technologies) |  | Citrate buffer (pH 6.0) |
| **WT1** |  | Rabbit polyclonal antibodies (No. sc-192, 1:400; Santa Cruz Biotechnology, Inc., Santa Cruz, CA, USA) |  | Biotinylated goat anti-rabbit IgG antibodies (SABPO kit, Nichirei) |  | Alexa Fluor labeled donkey anti-rabbit IgG antibodies (1:500, Life Technologies) |  | **-** |  | Citrate buffer (pH 6.0) |
| **Incubation** |  | 4°C, overnight |  | Room temperature, 30 min |  | Room temperature, 30 min |  | Room temperature, 60 min |  | Heating, 105°C, 15 min |
| IHC: immunohistochemistry. IF: immunofluorescence. WB: western blotting. WT1: Wilms’ tumor 1. NMIIA: myosin, heavy polypeptide 9, non-muscle. IBL: Immuno-Biological Laboratories | | | | | | | | | | |
