# Supplementary material for: Decreased miR-26a Expression Correlates with the Progression of Podocyte Injury in Autoimmune Glomerulonephritis
Source: PLoS One. 2014 Oct 17;9(10):e110383. doi: 10.1371/journal.pone.0110383 (PMC4201534; doi:10.1371/journal.pone.0110383)
Supplement: Table S4 — Primers and probes used in this study. (DOCX) [file pone.0110383.s004.docx]

**Table S4. Primers and probes used in this study.**

| **Symbol** | **Species** | **Entrez ID** | **Forward (5'-3')** | **Reverse (5'-3')** | **Product size (bp)** | **Application** |
| --- | --- | --- | --- | --- | --- | --- |
| *Acta1* | Mouse | 11459 | CGTGAAGCCTCACTTCCTAC | ACGTAGGAGTCCTTCTGACC | 232 | real-time PCR |
| *Acta2* | Mouse | 11475 | ATAACCCTTCAGCGTTCAGCC | CCAACCATTACTCCCTGATGTCTG | 239 | real-time PCR |
| *Actb* | Mouse | 11461 | ACTGCTCTGGCTCCTAGCAC | CAGCTCAGTAACAGTCCGCC | 196 | real-time PCR (normalization) |
| *Actb* | Mouse | 11461 | TCCTTCGTTGCCGGTCCACA | TGGGCCTCGTCACCCACATA | 213 | real-time PCR |
| *Actg1* | Mouse | 11465 | CGGCTTACACTGCGCTTCTT | GTGCGGCGATTTCTTCTTCC | 81 | real-time PCR |
| *Actg2* | Mouse | 11468 | GTATTTCTGCCAAAGACACCACG | ACATAGCTGTCTTTCTGGCCC | 261 | real-time PCR |
| *Actn4* | Mouse | 60595 | TCCAGGACATCTCTGTGGAAG | CATTGTTTAGGTTGGTGACTGG | 216 | real-time PCR |
| *Cd2ap* | Mouse | 12488 | CAAGATGCCTGGAAGACGA | GCACTTGAAGGTGTTGAAAGAG | 177 | real-time PCR |
| *Gapdh* | Mouse | 14433 | CCGCATCTTCTTGTGCAG | TGCCGTGAGTGGAGTCATAC | 199 | real-time PCR (normalization) |
| *Il1b* | Mouse | 16176 | AAGGAGAACCAAGCAACGAC | AACTCTGCAGACTCAAACTCCAC | 208 | real-time PCR |
| *Il6* | Mouse | 6193 | TGTATGAACAACGATGATGCAC | TGGTACTCCAGAAGACCAGAGG | 137 | real-time PCR |
| *Myh9* | Mouse | 17886 | AAGGACCAGGCTGACAAGG | GTCACGACAAATGGCAGGTC | 209 | real-time PCR |
| *Nphs1* | Mouse | 54631 | ACCTGTATGACGAGGTGGAGAG | TCGTGAAGAGTCTCACACCAG | 218 | real-time PCR |
| *Nphs2* | Mouse | 170484 | AAGGTTGATCTCCGTCTCCAG | TTCCATGCGGTAGTAGCAGAC | 105 | real-time PCR |
| *Podxl* | Mouse | 27205 | TCCTAAGGCCGTGTATGAGC | GATGCCATGCAGACGATG | 153 | real-time PCR |
| *Synpo* | Mouse | 104027 | CATCGGACCTTCTTCCTGTG | TCGGAGTCTGTGGGTGAG | 90 | real-time PCR |
| *Tnf* | Mouse | 21926 | CGAGTGACAAGCCTGTAGCC | GAGAACCTGGGAGTAGACAAGG | 167 | real-time PCR |
| *Vim* | Mouse | 22352 | CAGGATTTCTCTGCCTCTGC | TCAAGGTCATCGTGATGCTG | 171 | real-time PCR |
| *Wt1* | Mouse | 22431 | GGTATGAGAGTGAGAACCACACG | AGATGCTGACCGGACAAGAG | 137 | real-time PCR |
| **Symbol** | **Species** | **ID** | **Target sequence** | | **Application** | |
| miR-26a | Mouse, Human | MIMAT0000533 | UUCAAGUAAUCCAGGAUAGGCU | | TaqMan real-time PCR, *in situ* hybridization, miR-26a silencing | |
| U6 snRNA | Mouse, Human | NR_004394 | GCTGTACTGACTTGATGAAAGTACTTTTGAACCCTTTTCCATCTGATG | | TaqMan real-time PCR (normalization) | |
