# Supplementary material for: Decreased miR-26a Expression Correlates with the Progression of Podocyte Injury in Autoimmune Glomerulonephritis
Source: PLoS One. 2014 Oct 17;9(10):e110383. doi: 10.1371/journal.pone.0110383 (PMC4201534; doi:10.1371/journal.pone.0110383)
Supplement: Table S5 — Results of next-generation RNA sequensing. (DOCX) [file pone.0110383.s005.docx]

**Table S5. Results of next-generation RNA sequensing.**

| **Rank** | **Name** | **miRNA ID** | **Read number** | **Normalized value** | **Chromosome** | **Start** | **End** | **Strand** |
| --- | --- | --- | --- | --- | --- | --- | --- | --- |
| **1** | **miR-26a-5p** | **MI0000573_1** | **470926** | **18.8** | **chr9** | **118940929** | **118940950** | **+** |
| **2** | **miR-26a-5p** | **MI0000706_1** | **470926** | **18.8** | **chr10** | **126432599** | **126432620** | **+** |
| 3 | miR-126-5p | MI0000153_1 | 422785 | 18.6 | chr2 | 26446885 | 26446905 | + |
| 4 | miR-27b-3p | MI0000142_2 | 316117 | 18.2 | chr13 | 63402068 | 63402088 | + |
| 5 | miR-22-3p | MI0000570_2 | 284048 | 18.1 | chr11 | 75277274 | 75277295 | + |
| 6 | miR-10a-5p | MI0000685_1 | 165375 | 17.3 | chr11 | 96178500 | 96178522 | + |
| 7 | let-7f-5p | MI0000563_1 | 151579 | 17.2 | chrX | 148346896 | 148346917 | + |
| 8 | miR-10b-5p | MI0000221_1 | 140437 | 17.1 | chr2 | 74564131 | 74564153 | + |
| 9 | miR-192-5p | MI0000551_1 | 106205 | 16.6 | chr19 | 6264857 | 6264877 | + |
| 10 | miR-191-5p | MI0000233_1 | 93518 | 16.5 | chr9 | 108470656 | 108470678 | + |
| 11 | let-7c-5p | MI0000559_1 | 91058 | 16.4 | chr16 | 77599917 | 77599938 | + |
| 12 | let-7c-5p | MI0000560_1 | 91058 | 16.4 | chr15 | 85537046 | 85537067 | + |
| 13 | miR-30a-5p | MI0000144_1 | 80942 | 16.3 | chr1 | 23279113 | 23279134 | + |
| 14 | miR-16-5p | MI0000566_1 | 66403 | 16.0 | chr3 | 68813840 | 68813861 | + |
| 15 | let-7a-5p | MI0000557_1 | 61346 | 15.9 | chr9 | 41344815 | 41344836 | + |
| 16 | miR-181a-5p | MI0000223_1 | 55190 | 15.7 | chr2 | 38708261 | 38708283 | + |
| 17 | miR-181a-5p | MI0000697_1 | 55190 | 15.7 | chr1 | 139863045 | 139863067 | + |
| 18 | miR-126-3p | MI0000153_2 | 49798 | 15.6 | chr2 | 26446922 | 26446943 | + |
| 19 | let-7g-5p | MI0000137_1 | 39522 | 15.2 | chr9 | 106081177 | 106081198 | + |
| 20 | miR-30c-5p | MI0000548_1 | 26943 | 14.7 | chr1 | 23298553 | 23298575 | + |
| 21 | miR-99b-5p | MI0000147_1 | 22781 | 14.4 | chr17 | 17967158 | 17967179 | + |
| 22 | miR-23b-3p | MI0000141_2 | 21562 | 14.3 | chr13 | 63401837 | 63401857 | + |
| 23 | let-7b-5p | MI0000558_1 | 18145 | 14.1 | chr15 | 85537755 | 85537776 | + |
| 24 | miR-204-5p | MI0000247_1 | 16817 | 14.0 | chr19 | 22825100 | 22825121 | + |
| 25 | miR-26b-5p | MI0000575_1 | 16045 | 13.9 | chr1 | 74440898 | 74440918 | + |
| 26 | miR-103-3p | MI0000588_2 | 15544 | 13.9 | chr2 | 131113839 | 131113861 | + |
| 27 | miR-103-3p | MI0000587_2 | 15544 | 13.9 | chr11 | 35595949 | 35595971 | + |
| 28 | miR-23a-3p | MI0000571_2 | 14993 | 13.8 | chr8 | 86732462 | 86732482 | + |
| 29 | miR-30a-3p | MI0000144_2 | 14108 | 13.7 | chr1 | 23279154 | 23279175 | + |
| 30 | miR-127-3p | MI0000154_2 | 11760 | 13.5 | chr12 | 110831098 | 110831119 | + |
| 31 | miR-125a-5p | MI0000151_1 | 10069 | 13.2 | chr17 | 17967781 | 17967804 | + |
| 32 | miR-27a-3p | MI0000578_2 | 9921 | 13.2 | chr8 | 86732626 | 86732646 | + |
| 33 | let-7e-5p | MI0000561_1 | 7063 | 12.7 | chr17 | 17967330 | 17967351 | + |
| 34 | miR-195-5p | MI0000237_1 | 6099 | 12.5 | chr11 | 70048564 | 70048584 | + |
| 35 | miR-24-3p | MI0000231_2 | 5776 | 12.4 | chr13 | 63402559 | 63402580 | + |
| 36 | miR-24-3p | MI0000572_2 | 5776 | 12.4 | chr8 | 86732774 | 86732795 | + |
| 37 | miR-199b-3p | MI0000714_2 | 5749 | 12.4 | chr2 | 32174044 | 32174065 | + |
| 38 | miR-199a-3p | MI0000713_2 | 5749 | 12.4 | chr1 | 164148014 | 164148035 | + |
| 39 | miR-301a-3p | MI0000401_2 | 5285 | 12.3 | chr11 | 86926556 | 86926578 | + |
| 40 | miR-186-5p | MI0000228_1 | 5222 | 12.3 | chr3 | 157207249 | 157207270 | + |
| 41 | miR-486-5p | MI0003493_1 | 4610 | 12.1 | chr8 | 24253059 | 24253080 | + |
| 42 | miR-29c-3p | MI0000577_2 | 4059 | 11.9 | chr1 | 196863794 | 196863815 | + |
| 43 | miR-196a-5p | MI0000553_1 | 3901 | 11.9 | chr15 | 102803796 | 102803817 | + |
| 44 | miR-196a-5p | MI0000552_1 | 3901 | 11.9 | chr11 | 96126501 | 96126522 | + |
| 45 | miR-98-5p | MI0000586_1 | 3807 | 11.8 | chrX | 148347772 | 148347793 | + |
| 46 | miR-125b-5p | MI0000152_1 | 2911 | 11.5 | chr16 | 77646524 | 77646545 | + |
| 47 | miR-125b-5p | MI0000725_1 | 2911 | 11.5 | chr9 | 41390023 | 41390044 | + |
| 48 | miR-24-2-5p | MI0000572_1 | 2794 | 11.4 | chr8 | 86732738 | 86732759 | + |
| 49 | miR-142-5p | MI0000167_1 | 2748 | 11.4 | chr11 | 87570369 | 87570389 | + |
| 50 | miR-411-5p | MI0001163_1 | 2656 | 11.3 | chr12 | 110948400 | 110948420 | + |
| 51 | miR-194-5p | MI0000236_1 | 2419 | 11.2 | chr1 | 187137204 | 187137225 | + |
| 52 | miR-194-5p | MI0000733_1 | 2419 | 11.2 | chr19 | 6264658 | 6264679 | + |
| 53 | miR-29b-3p | MI0000712_2 | 2393 | 11.2 | chr1 | 196863285 | 196863307 | + |
| 54 | miR-340-5p | MI0000623_1 | 2341 | 11.1 | chr11 | 49883222 | 49883243 | + |
| 55 | miR-30c-2-3p | MI0000548_2 | 2235 | 11.1 | chr1 | 23298593 | 23298614 | + |
| 56 | miR-99a-5p | MI0000146_1 | 1971 | 10.9 | chr16 | 77599185 | 77599206 | + |
| 57 | miR-181b-5p | MI0000823_1 | 1605 | 10.6 | chr2 | 38709365 | 38709387 | + |
| 58 | miR-181b-5p | MI0000723_1 | 1605 | 10.6 | chr1 | 139863227 | 139863249 | + |
| 59 | miR-335-3p | MI0000817_2 | 1529 | 10.5 | chr6 | 30691354 | 30691375 | + |
| 60 | miR-199a-5p | MI0000713_1 | 1521 | 10.5 | chr1 | 164147975 | 164147997 | + |
| 61 | miR-497-5p | MI0004636_1 | 1416 | 10.4 | chr11 | 70048232 | 70048253 | + |
| 62 | let-7c-2-3p | MI0000560_2 | 1387 | 10.4 | chr15 | 85537094 | 85537115 | + |
| 63 | let-7b-3p | MI0000558_2 | 1307 | 10.3 | chr15 | 85537809 | 85537830 | + |
| 64 | miR-148b-3p | MI0000617_2 | 1269 | 10.3 | chr15 | 103115616 | 103115637 | + |
| 65 | miR-574-3p | MI0005518_2 | 1094 | 10.0 | chr5 | 65361603 | 65361624 | + |
| 66 | miR-484 | MI0003491_1 | 1047 | 10.0 | chr16 | 14159723 | 14159744 | + |
| 67 | miR-19b-3p | MI0000718_2 | 1022 | 9.9 | chr14 | 115443580 | 115443602 | + |
| 68 | miR-434-3p | MI0001526_2 | 1008 | 9.9 | chr12 | 110832775 | 110832796 | + |
| 69 | miR-152-3p | MI0000174_2 | 953 | 9.8 | chr11 | 96711753 | 96711773 | + |
| 70 | miR-320-3p | MI0000704_2 | 937 | 9.8 | chr14 | 70843364 | 70843385 | + |
| 71 | miR-193-3p | MI0000235_2 | 885 | 9.7 | chr11 | 79525511 | 79525532 | + |
| 72 | miR-335-5p | MI0000817_1 | 878 | 9.7 | chr6 | 30691314 | 30691336 | + |
| 73 | miR-100-5p | MI0000692_1 | 833 | 9.7 | chr9 | 41339520 | 41339541 | + |
| 74 | miR-20a-5p | MI0000568_1 | 822 | 9.6 | chr14 | 115443405 | 115443427 | + |
| 75 | miR-872-5p | MI0005549_1 | 774 | 9.5 | chr4 | 94331858 | 94331878 | + |
| 76 | miR-451 | MI0001730_1 | 761 | 9.5 | chr11 | 77886688 | 77886709 | + |
| 77 | miR-17-5p | MI0000687_1 | 757 | 9.5 | chr14 | 115442906 | 115442928 | + |
| 78 | miR-872-3p | MI0005549_2 | 744 | 9.5 | chr4 | 94331897 | 94331918 | + |
| 79 | miR-150-5p | MI0000172_1 | 728 | 9.5 | chr7 | 52377132 | 52377153 | + |
| 80 | miR-132-3p | MI0000158_2 | 682 | 9.4 | chr11 | 74987225 | 74987246 | + |
| 81 | miR-15b-5p | MI0000140_1 | 674 | 9.3 | chr3 | 68813697 | 68813718 | + |
| 82 | miR-140-5p | MI0000165_1 | 653 | 9.3 | chr8 | 110075149 | 110075170 | + |
| 83 | miR-365-3p | MI0000768_2 | 653 | 9.3 | chr16 | 13453988 | 13454009 | + |
| 84 | miR-365-3p | MI0001645_2 | 653 | 9.3 | chr11 | 79539969 | 79539990 | + |
| 85 | miR-326-3p | MI0000598_2 | 651 | 9.3 | chr7 | 106700838 | 106700858 | + |
| 86 | miR-92a-3p | MI0000719_2 | 610 | 9.2 | chr14 | 115443698 | 115443718 | + |
| 87 | miR-28-3p | MI0000690_2 | 530 | 9.0 | chr16 | 24827994 | 24828015 | + |
| 88 | miR-146b-5p | MI0004665_1 | 519 | 9.0 | chr19 | 46417280 | 46417301 | + |
| 89 | miR-203-3p | MI0000246_2 | 460 | 8.8 | chr12 | 113369138 | 113369159 | + |
| 90 | miR-214-3p | MI0000698_2 | 450 | 8.8 | chr1 | 164153569 | 164153590 | + |
| 91 | miR-34a-5p | MI0000584_1 | 445 | 8.7 | chr4 | 149442582 | 149442603 | + |
| 92 | miR-101b-3p | MI0000649_2 | 379 | 8.5 | chr19 | 29209829 | 29209849 | + |
| 93 | miR-342-3p | MI0000627_2 | 374 | 8.5 | chr12 | 109896890 | 109896912 | + |
| 94 | miR-381-3p | MI0000798_2 | 374 | 8.5 | chr12 | 110965080 | 110965101 | + |
| 95 | miR-139-5p | MI0000693_1 | 372 | 8.5 | chr7 | 108623896 | 108623917 | + |
| 96 | miR-27b-5p | MI0000142_1 | 370 | 8.5 | chr13 | 63402026 | 63402047 | + |
| 97 | miR-28-5p | MI0000690_1 | 368 | 8.5 | chr16 | 24827954 | 24827975 | + |
| 98 | miR-152-5p | MI0000174_1 | 365 | 8.5 | chr11 | 96711714 | 96711737 | + |
| 99 | miR-1839-5p | MI0009991_1 | 355 | 8.4 | chr7 | 88674805 | 88674826 | + |
| 100 | miR-410-3p | MI0001161_2 | 344 | 8.4 | chr12 | 110981974 | 110981994 | + |
| 101 | miR-218-5p | MI0000700_1 | 330 | 8.3 | chr5 | 48615205 | 48615225 | + |
| 102 | miR-218-5p | MI0000701_1 | 330 | 8.3 | chr11 | 35430342 | 35430362 | + |
| 103 | miR-181a-1-3p | MI0000697_2 | 316 | 8.3 | chr1 | 139863085 | 139863106 | + |
| 104 | miR-615-3p | MI0005004_2 | 264 | 8.0 | chr15 | 102845400 | 102845421 | + |
| 105 | miR-149-5p | MI0000171_1 | 227 | 7.8 | chr1 | 94746958 | 94746980 | + |
| 106 | miR-425-5p | MI0001447_1 | 212 | 7.7 | chr9 | 108471120 | 108471142 | + |
| 107 | miR-455-5p | MI0004679_1 | 201 | 7.6 | chr4 | 62917901 | 62917922 | + |
| 108 | miR-22-5p | MI0000570_1 | 189 | 7.5 | chr11 | 75277236 | 75277257 | + |
| 109 | miR-9-5p | MI0000720_1 | 185 | 7.5 | chr3 | 88019535 | 88019557 | + |
| 110 | miR-9-5p | MI0000157_1 | 185 | 7.5 | chr13 | 83878426 | 83878448 | + |
| 111 | miR-9-5p | MI0000721_1 | 185 | 7.5 | chr7 | 86650165 | 86650187 | + |
| 112 | let-7e-3p | MI0000561_2 | 181 | 7.5 | chr17 | 17967375 | 17967396 | + |
| 113 | miR-652-3p | MI0004965_2 | 167 | 7.3 | chrX | 139173603 | 139173623 | + |
| 114 | miR-324-5p | MI0000595_1 | 162 | 7.3 | chr11 | 69825562 | 69825584 | + |
| 115 | miR-1198-5p | MI0006306_1 | 148 | 7.2 | chrX | 7384268 | 7384289 | + |
| 116 | miR-24-1-5p | MI0000231_1 | 147 | 7.2 | chr13 | 63402521 | 63402543 | + |
| 117 | miR-223-3p | MI0000703_2 | 147 | 7.2 | chrX | 93438223 | 93438244 | + |
| 118 | miR-676-3p | MI0005003_2 | 141 | 7.1 | chrX | 97576490 | 97576510 | + |
| 119 | miR-142-3p | MI0000167_2 | 125 | 6.9 | chr11 | 87570405 | 87570427 | + |
| 120 | miR-19a-3p | MI0000688_2 | 118 | 6.8 | chr14 | 115443270 | 115443292 | + |
| 121 | miR-300-3p | MI0000400_2 | 117 | 6.8 | chr12 | 110962573 | 110962594 | + |
| 122 | miR-1843b-5p | MI0016971_1 | 117 | 6.8 | chr1 | 161270493 | 161270513 | + |
| 123 | miR-128-3p | MI0000155_2 | 111 | 6.7 | chr1 | 130098981 | 130099001 | + |
| 124 | miR-195-3p | MI0000237_2 | 110 | 6.7 | chr11 | 70048602 | 70048623 | + |
| 125 | miR-10a-3p | MI0000685_2 | 105 | 6.7 | chr11 | 96178541 | 96178562 | + |
| 126 | miR-144-5p | MI0000168_1 | 104 | 6.7 | chr11 | 77886512 | 77886534 | + |
| 127 | miR-214-5p | MI0000698_1 | 101 | 6.6 | chr1 | 164153528 | 164153549 | + |
| 128 | miR-193b-3p | MI0005484_2 | 97 | 6.6 | chr16 | 13449663 | 13449684 | + |
| 129 | miR-671-3p | MI0004133_2 | 96 | 6.5 | chr5 | 24097989 | 24098009 | + |
| 130 | miR-26b-3p | MI0000575_2 | 93 | 6.5 | chr1 | 74440934 | 74440955 | + |
| 131 | miR-144-3p | MI0000168_2 | 87 | 6.4 | chr11 | 77886549 | 77886568 | + |
| 132 | miR-379-5p | MI0000796_1 | 85 | 6.4 | chr12 | 110947275 | 110947295 | + |
| 133 | miR-27a-5p | MI0000578_1 | 84 | 6.3 | chr8 | 86732584 | 86732605 | + |
| 134 | miR-133a-3p | MI0000820_2 | 80 | 6.3 | chr2 | 180133142 | 180133163 | + |
| 135 | miR-125b-2-3p | MI0000152_2 | 76 | 6.2 | chr16 | 77646563 | 77646584 | + |
| 136 | miR-26a-2-3p | MI0000706_2 | 70 | 6.1 | chr10 | 126432637 | 126432658 | + |
| 137 | miR-140-3p | MI0000165_2 | 69 | 6.1 | chr8 | 110075188 | 110075208 | + |
| 138 | miR-129-5p | MI0000222_1 | 68 | 6.0 | chr6 | 28972624 | 28972644 | + |
| 139 | miR-409-3p | MI0001160_2 | 65 | 6.0 | chr12 | 110981414 | 110981435 | + |
| 140 | miR-155-5p | MI0000177_1 | 64 | 6.0 | chr16 | 84714388 | 84714410 | + |
| 141 | miR-511-3p | MI0005554_2 | 63 | 5.9 | chr2 | 14182678 | 14182699 | + |
| 142 | miR-488-3p | MI0004633_2 | 62 | 5.9 | chr1 | 160435820 | 160435840 | + |
| 143 | miR-98-3p | MI0000586_2 | 60 | 5.9 | chrX | 148347830 | 148347851 | + |
| 144 | miR-148b-5p | MI0000617_1 | 60 | 5.9 | chr15 | 103115579 | 103115602 | + |
| 145 | miR-129-1-3p | MI0000222_2 | 58 | 5.8 | chr6 | 28972667 | 28972688 | + |
| 146 | miR-212-5p | MI0000696_1 | 58 | 5.8 | chr11 | 74986905 | 74986927 | + |
| 147 | miR-33-5p | MI0000707_1 | 55 | 5.7 | chr15 | 82028557 | 82028577 | + |
| 148 | miR-99b-3p | MI0000147_2 | 55 | 5.7 | chr17 | 17967196 | 17967217 | + |
| 149 | miR-1a-3p | MI0000139_2 | 54 | 5.7 | chr2 | 180123801 | 180123822 | + |
| 150 | miR-134-5p | MI0000160_1 | 50 | 5.6 | chr12 | 110972355 | 110972376 | + |
| 151 | miR-29c-5p | MI0000577_1 | 49 | 5.6 | chr1 | 196863756 | 196863777 | + |
| 152 | miR-125a-3p | MI0000151_2 | 48 | 5.5 | chr17 | 17967819 | 17967840 | + |
| 153 | miR-330-5p | MI0000607_1 | 46 | 5.5 | chr7 | 19766835 | 19766856 | + |
| 154 | miR-196a-2-3p | MI0000553_2 | 45 | 5.4 | chr15 | 102803832 | 102803853 | + |
| 155 | miR-153-3p | MI0000175_2 | 45 | 5.4 | chr12 | 118489333 | 118489354 | + |
| 156 | miR-434-5p | MI0001526_1 | 44 | 5.4 | chr12 | 110832738 | 110832759 | + |
| 157 | miR-376c-3p | MI0003533_2 | 39 | 5.2 | chr12 | 110960980 | 110961000 | + |
| 158 | miR-92a-1-5p | MI0000719_1 | 38 | 5.2 | chr14 | 115443659 | 115443681 | + |
| 159 | miR-674-3p | MI0004611_2 | 37 | 5.2 | chr2 | 117010922 | 117010943 | + |
| 160 | miR-664-3p | MI0012531_2 | 37 | 5.2 | chr1 | 187066891 | 187066912 | + |
| 161 | miR-154-5p | MI0000176_1 | 36 | 5.1 | chr12 | 110976648 | 110976669 | + |
| 162 | miR-708-5p | MI0004692_1 | 35 | 5.1 | chr7 | 103397960 | 103397982 | + |
| 163 | miR-199b-5p | MI0000714_1 | 35 | 5.1 | chr2 | 32174005 | 32174027 | + |
| 164 | miR-136-5p | MI0000162_1 | 34 | 5.0 | chr12 | 110833541 | 110833562 | + |
| 165 | miR-212-3p | MI0000696_2 | 34 | 5.0 | chr11 | 74986945 | 74986966 | + |
| 166 | miR-449a-5p | MI0001649_1 | 32 | 5.0 | chr13 | 113827757 | 113827778 | + |
| 167 | miR-674-5p | MI0004611_1 | 32 | 5.0 | chr2 | 117010887 | 117010908 | + |
| 168 | miR-340-3p | MI0000623_2 | 30 | 4.9 | chr11 | 49883264 | 49883285 | + |
| 169 | miR-1839-3p | MI0009991_2 | 29 | 4.8 | chr7 | 88674844 | 88674866 | + |
| 170 | miR-18a-5p | MI0000567_1 | 29 | 4.8 | chr14 | 115443089 | 115443111 | + |
| 171 | miR-676-5p | MI0005003_1 | 29 | 4.8 | chrX | 97576453 | 97576474 | + |
| 172 | miR-455-3p | MI0004679_2 | 28 | 4.8 | chr4 | 62917938 | 62917958 | + |
| 173 | miR-1964-3p | MI0009961_2 | 26 | 4.7 | chr7 | 30558363 | 30558384 | + |
| 174 | miR-138-5p | MI0000722_1 | 25 | 4.6 | chr9 | 122592016 | 122592038 | + |
| 175 | miR-138-5p | MI0000164_1 | 25 | 4.6 | chr8 | 96848212 | 96848234 | + |
| 176 | miR-382-5p | MI0000799_1 | 24 | 4.5 | chr12 | 110971991 | 110972012 | + |
| 177 | miR-369-3p | MI0003535_2 | 24 | 4.5 | chr12 | 110981676 | 110981696 | + |
| 178 | miR-26a-1-3p | MI0000573_2 | 22 | 4.4 | chr9 | 118940968 | 118940989 | + |
| 179 | miR-33-3p | MI0000707_2 | 22 | 4.4 | chr15 | 82028597 | 82028618 | + |
| 180 | miR-433-3p | MI0001525_2 | 21 | 4.3 | chr12 | 110829991 | 110830012 | + |
| 181 | miR-598-3p | MI0005556_2 | 21 | 4.3 | chr14 | 64346073 | 64346094 | + |
| 182 | miR-17-3p | MI0000687_2 | 21 | 4.3 | chr14 | 115442943 | 115442964 | + |
| 183 | miR-708-3p | MI0004692_2 | 19 | 4.2 | chr7 | 103398006 | 103398027 | + |
| 184 | miR-582-3p | MI0006127_2 | 18 | 4.1 | chr13 | 110114984 | 110115004 | + |
| 185 | miR-345-5p | MI0000632_1 | 18 | 4.1 | chr12 | 110075199 | 110075220 | + |
| 186 | miR-802-5p | MI0004249_1 | 17 | 4.0 | chr16 | 93369982 | 93370003 | + |
| 187 | miR-127-5p | MI0000154_1 | 16 | 4.0 | chr12 | 110831064 | 110831085 | + |
| 188 | miR-341-3p | MI0000625_2 | 16 | 4.0 | chr12 | 110849766 | 110849786 | + |
| 189 | miR-667-3p | MI0004196_2 | 16 | 4.0 | chr12 | 110958271 | 110958293 | + |
| 190 | miR-376b-3p | MI0001162_2 | 16 | 4.0 | chr12 | 110961718 | 110961738 | + |
| 191 | miR-330-3p | MI0000607_2 | 16 | 4.0 | chr7 | 19766874 | 19766896 | + |
| 192 | let-7f-2-3p | MI0000563_2 | 15 | 3.9 | chrX | 148346946 | 148346966 | + |
| 193 | miR-15b-3p | MI0000140_2 | 15 | 3.9 | chr3 | 68813735 | 68813756 | + |
| 194 | miR-299-3p | MI0000399_2 | 15 | 3.9 | chr12 | 110948886 | 110948907 | + |
| 195 | miR-425-3p | MI0001447_2 | 14 | 3.8 | chr9 | 108471161 | 108471181 | + |
| 196 | miR-379-3p | MI0000796_2 | 14 | 3.8 | chr12 | 110947312 | 110947333 | + |
| 197 | miR-329-3p | MI0000605_2 | 13 | 3.7 | chr12 | 110951751 | 110951772 | + |
| 198 | miR-125b-1-3p | MI0000725_2 | 12 | 3.5 | chr9 | 41390063 | 41390084 | + |
| 199 | miR-16-2-3p | MI0000566_2 | 12 | 3.5 | chr3 | 68813882 | 68813903 | + |
| 200 | miR-615-5p | MI0005004_1 | 12 | 3.5 | chr15 | 102845357 | 102845378 | + |
| 201 | miR-802-3p | MI0004249_2 | 12 | 3.5 | chr16 | 93370020 | 93370041 | + |
| 202 | miR-673-5p | MI0004601_1 | 12 | 3.5 | chr12 | 110810214 | 110810235 | + |
| 203 | miR-3105-3p | MI0014102_2 | 12 | 3.5 | chr7 | 151195209 | 151195230 | + |
| 204 | miR-409-5p | MI0001160_1 | 11 | 3.4 | chr12 | 110981382 | 110981404 | + |
| 205 | miR-669c-5p | MI0004673_1 | 11 | 3.4 | chr2 | 10430946 | 10430967 | + |
| 206 | miR-215-5p | MI0000974_1 | 11 | 3.4 | chr1 | 187137489 | 187137509 | + |
| 207 | miR-192-3p | MI0000551_2 | 10 | 3.3 | chr19 | 6264900 | 6264921 | + |
| 208 | miR-495-3p | MI0004639_2 | 10 | 3.3 | chr12 | 110957005 | 110957026 | + |
| 209 | miR-671-5p | MI0004133_1 | 10 | 3.3 | chr5 | 24097950 | 24097972 | + |
| 210 | miR-487b-3p | MI0003534_2 | 10 | 3.3 | chr12 | 110965592 | 110965613 | + |
| 211 | miR-411-3p | MI0001163_2 | 10 | 3.3 | chr12 | 110948435 | 110948456 | + |
| 212 | miR-193-5p | MI0000235_1 | 10 | 3.3 | chr11 | 79525477 | 79525498 | + |
| 213 | miR-299-5p | MI0000399_1 | 8 | 3.0 | chr12 | 110948854 | 110948875 | + |
| 214 | miR-204-3p | MI0000247_2 | 7 | 2.8 | chr19 | 22825139 | 22825159 | + |
| 215 | miR-186-3p | MI0000228_2 | 7 | 2.8 | chr3 | 157207288 | 157207309 | + |
| 216 | miR-365-2-5p | MI0001645_1 | 7 | 2.8 | chr11 | 79539930 | 79539952 | + |
| 217 | miR-136-3p | MI0000162_2 | 7 | 2.8 | chr12 | 110833576 | 110833597 | + |
| 218 | miR-99a-3p | MI0000146_2 | 7 | 2.8 | chr16 | 77599222 | 77599242 | + |
| 219 | miR-376b-5p | MI0001162_1 | 7 | 2.8 | chr12 | 110961681 | 110961702 | + |
| 220 | miR-3061-3p | MI0014023_2 | 7 | 2.8 | chr11 | 51940306 | 51940327 | + |
| 221 | let-7c-1-3p | MI0000559_2 | 6 | 2.5 | chr16 | 77599962 | 77599983 | + |
| 222 | miR-431-5p | MI0001524_1 | 6 | 2.5 | chr12 | 110828669 | 110828689 | + |
| 223 | miR-377-3p | MI0000794_2 | 6 | 2.5 | chr12 | 110978763 | 110978784 | + |
| 224 | miR-342-5p | MI0000627_1 | 6 | 2.5 | chr12 | 109896848 | 109896869 | + |
| 225 | miR-329-5p | MI0000605_1 | 6 | 2.5 | chr12 | 110951713 | 110951734 | + |
| 226 | miR-194-2-3p | MI0000733_2 | 5 | 2.3 | chr19 | 6264694 | 6264715 | + |
| 227 | miR-218-1-3p | MI0000700_2 | 5 | 2.3 | chr5 | 48615244 | 48615265 | + |
| 228 | miR-10b-3p | MI0000221_2 | 5 | 2.3 | chr2 | 74564171 | 74564192 | + |
| 229 | miR-345-3p | MI0000632_2 | 5 | 2.3 | chr12 | 110075237 | 110075258 | + |
| 230 | miR-139-3p | MI0000693_2 | 5 | 2.3 | chr7 | 108623932 | 108623953 | + |
| 231 | miR-582-5p | MI0006127_1 | 5 | 2.3 | chr13 | 110114947 | 110114968 | + |
| 232 | miR-301a-5p | MI0000401_1 | 5 | 2.3 | chr11 | 86926519 | 86926540 | + |
| 233 | miR-543-3p | MI0003519_2 | 5 | 2.3 | chr12 | 110955514 | 110955535 | + |
| 234 | miR-1947-5p | MI0009937_1 | 5 | 2.3 | chr16 | 33105461 | 33105482 | + |
| 235 | miR-337-3p | MI0000615_2 | 5 | 2.3 | chr12 | 110824059 | 110824079 | + |
| 236 | miR-668-3p | MI0004134_2 | 5 | 2.3 | chr12 | 110972984 | 110973007 | + |
| 237 | miR-3060-3p | MI0014022_2 | 5 | 2.3 | chr11 | 4039416 | 4039437 | + |
| 238 | miR-132-5p | MI0000158_1 | 5 | 2.3 | chr11 | 74987188 | 74987209 | + |
| 239 | miR-211-5p | MI0000708_1 | 4 | 2.0 | chr7 | 71350717 | 71350738 | + |
| 240 | miR-128-1-5p | MI0000155_1 | 4 | 2.0 | chr1 | 130098946 | 130098966 | + |
| 241 | miR-18a-3p | MI0000567_2 | 4 | 2.0 | chr14 | 115443130 | 115443151 | + |
| 242 | miR-485-5p | MI0003492_1 | 4 | 2.0 | chr12 | 110973120 | 110973141 | + |
| 243 | miR-146b-3p | MI0004665_2 | 4 | 2.0 | chr19 | 46417318 | 46417339 | + |
| 244 | miR-3109-3p | MI0014106_2 | 4 | 2.0 | chr9 | 69304804 | 69304824 | + |
| 245 | miR-191-3p | MI0000233_2 | 4 | 2.0 | chr9 | 108470698 | 108470719 | + |
| 246 | miR-652-5p | MI0004965_1 | 4 | 2.0 | chrX | 139173563 | 139173586 | + |
| 247 | miR-879-5p | MI0005472_1 | 4 | 2.0 | chr5 | 9375714 | 9375734 | + |
| 248 | miR-701-5p | MI0004685_1 | 4 | 2.0 | chr5 | 111433189 | 111433209 | + |
| 249 | miR-190b-5p | MI0005478_1 | 3 | 1.5 | chr3 | 89873952 | 89873972 | + |
| 250 | miR-7a-5p | MI0000729_1 | 3 | 1.5 | chr7 | 86033181 | 86033203 | + |
| 251 | miR-669d-5p | MI0006281_1 | 3 | 1.5 | chr2 | 10390011 | 10390032 | + |
| 252 | miR-669d-5p | MI0014051_1 | 3 | 1.5 | chr2 | 10393285 | 10393306 | + |
| 253 | miR-3089-3p | MI0014082_2 | 3 | 1.5 | chr2 | 30576779 | 30576801 | + |
| 254 | miR-1954 | MI0009949_1 | 3 | 1.5 | chr2 | 32507862 | 32507882 | + |
| 255 | miR-664-5p | MI0012531_1 | 3 | 1.5 | chr1 | 187066854 | 187066874 | + |
| 256 | miR-1948-3p | MI0009939_2 | 3 | 1.5 | chr18 | 12873371 | 12873392 | + |
| 257 | miR-223-5p | MI0000703_1 | 3 | 1.5 | chrX | 93438181 | 93438203 | + |
| 258 | miR-673-3p | MI0004601_2 | 3 | 1.5 | chr12 | 110810254 | 110810276 | + |
| 259 | miR-1193-3p | MI0006298_2 | 3 | 1.5 | chr12 | 110953960 | 110953980 | + |
| 260 | miR-203-5p | MI0000246_1 | 3 | 1.5 | chr12 | 113369100 | 113369121 | + |
| 261 | miR-196a-1-3p | MI0000552_2 | 3 | 1.5 | chr11 | 96126539 | 96126560 | + |
| 262 | miR-677-5p | MI0004634_1 | 3 | 1.5 | chr10 | 127522347 | 127522368 | + |
| 263 | miR-147-3p | MI0005482_2 | 2 | 1.0 | chr2 | 122466586 | 122466607 | + |
| 264 | let-7g-3p | MI0000137_2 | 2 | 1.0 | chr9 | 106081233 | 106081254 | + |
| 265 | miR-19b-1-5p | MI0000718_1 | 2 | 1.0 | chr14 | 115443542 | 115443564 | + |
| 266 | miR-324-3p | MI0000595_2 | 2 | 1.0 | chr11 | 69825597 | 69825616 | + |
| 267 | miR-133b-3p | MI0000821_2 | 2 | 1.0 | chr1 | 20672915 | 20672936 | + |
| 268 | miR-486-3p | MI0003493_2 | 2 | 1.0 | chr8 | 24253101 | 24253121 | + |
| 269 | miR-9-3p | MI0000720_2 | 2 | 1.0 | chr3 | 88019574 | 88019595 | + |
| 270 | miR-9-3p | MI0000157_2 | 2 | 1.0 | chr13 | 83878463 | 83878484 | + |
| 271 | miR-9-3p | MI0000721_2 | 2 | 1.0 | chr7 | 86650204 | 86650225 | + |
| 272 | miR-467c-5p | MI0005512_1 | 2 | 1.0 | chr2 | 10395568 | 10395589 | + |
| 273 | miR-467d-5p | MI0005513_1 | 2 | 1.0 | chr2 | 10429267 | 10429288 | + |
| 274 | miR-1955-3p | MI0009950_2 | 2 | 1.0 | chr2 | 92032192 | 92032212 | + |
| 275 | miR-3090-3p | MI0014083_2 | 2 | 1.0 | chr2 | 133390496 | 133390517 | + |
| 276 | miR-346-5p | MI0000634_1 | 2 | 1.0 | chr14 | 35707810 | 35707832 | + |
| 277 | miR-1191 | MI0006296_1 | 2 | 1.0 | chr7 | 27990634 | 27990654 | + |
| 278 | miR-3057-5p | MI0014020_1 | 2 | 1.0 | chr10 | 80734355 | 80734378 | + |
| 279 | miR-3098-5p | MI0014091_1 | 2 | 1.0 | chr6 | 125145825 | 125145846 | + |
| 280 | miR-490-3p | MI0005002_2 | 1 | 0.0 | chr6 | 36371800 | 36371821 | + |
| 281 | miR-193b-5p | MI0005484_1 | 1 | 0.0 | chr16 | 13449626 | 13449647 | + |
| 282 | miR-551b-3p | MI0004131_2 | 1 | 0.0 | chr3 | 29315801 | 29315821 | + |
| 283 | miR-103-2-5p | MI0000588_1 | 1 | 0.0 | chr2 | 131113802 | 131113824 | + |
| 284 | miR-181b-1-3p | MI0000723_2 | 1 | 0.0 | chr1 | 139863267 | 139863285 | + |
| 285 | miR-135a-5p | MI0000161_1 | 1 | 0.0 | chr9 | 106056471 | 106056493 | + |
| 286 | miR-23b-5p | MI0000141_1 | 1 | 0.0 | chr13 | 63401800 | 63401820 | + |
| 287 | miR-431-3p | MI0001524_2 | 1 | 0.0 | chr12 | 110828712 | 110828733 | + |
| 288 | miR-377-5p | MI0000794_1 | 1 | 0.0 | chr12 | 110978725 | 110978746 | + |
| 289 | miR-216b-5p | MI0004126_1 | 1 | 0.0 | chr11 | 28646204 | 28646225 | + |
| 290 | miR-133a-5p | MI0000820_1 | 1 | 0.0 | chr2 | 180133106 | 180133126 | + |
| 291 | miR-216a-5p | MI0000699_1 | 1 | 0.0 | chr11 | 28657018 | 28657039 | + |
| 292 | miR-135b-5p | MI0000646_1 | 1 | 0.0 | chr1 | 134094680 | 134094702 | + |
| 293 | miR-1197-3p | MI0006305_2 | 1 | 0.0 | chr12 | 110950606 | 110950626 | + |
| 294 | miR-376a-3p | MI0000793_2 | 1 | 0.0 | chr12 | 110962034 | 110962054 | + |
| 295 | miR-494-3p | MI0003532_2 | 1 | 0.0 | chr12 | 110953577 | 110953598 | + |
| 296 | miR-1298-5p | MI0004300_1 | 1 | 0.0 | chrX | 143499461 | 143499482 | + |
| 297 | miR-369-5p | MI0003535_1 | 1 | 0.0 | chr12 | 110981641 | 110981662 | + |
| 298 | miR-496-3p | MI0004589_2 | 1 | 0.0 | chr12 | 110977375 | 110977396 | + |
| 299 | miR-34a-3p | MI0000584_2 | 1 | 0.0 | chr4 | 149442626 | 149442646 | + |
| 300 | miR-5100 | MI0018008_1 | 1 | 0.0 | chr11 | 60542200 | 60542220 | + |
| 301 | miR-669f-5p | MI0006287_1 | 1 | 0.0 | chr2 | 10388875 | 10388898 | + |
| 302 | miR-669b-5p | MI0004666_1 | 1 | 0.0 | chr2 | 10389441 | 10389462 | + |
| 303 | miR-669l-5p | MI0009942_1 | 1 | 0.0 | chr2 | 10390616 | 10390637 | + |
| 304 | miR-467e-5p | MI0006128_1 | 1 | 0.0 | chr2 | 10427358 | 10427379 | + |
| 305 | miR-467e-3p | MI0006128_2 | 1 | 0.0 | chr2 | 10427395 | 10427416 | + |
| 306 | miR-297b-5p | MI0004674_1 | 1 | 0.0 | chr2 | 10433313 | 10433334 | + |
| 307 | miR-466d-3p | MI0005546_2 | 1 | 0.0 | chr2 | 10433644 | 10433664 | + |
| 308 | miR-5117-5p | MI0018026_1 | 1 | 0.0 | chr1 | 162967491 | 162967513 | + |
| 309 | miR-5117-3p | MI0018026_2 | 1 | 0.0 | chr1 | 162967547 | 162967565 | + |
| 310 | miR-215-3p | MI0000974_2 | 1 | 0.0 | chr1 | 187137526 | 187137546 | + |
| 311 | miR-1941-3p | MI0009930_2 | 1 | 0.0 | chr15 | 101199832 | 101199852 | + |
| 312 | miR-3075-5p | MI0014038_1 | 1 | 0.0 | chr14 | 26353937 | 26353957 | + |
| 313 | miR-3076-3p | MI0014039_2 | 1 | 0.0 | chr14 | 31385371 | 31385393 | + |
| 314 | miR-20a-3p | MI0000568_2 | 1 | 0.0 | chr14 | 115443441 | 115443462 | + |
| 315 | miR-1948-5p | MI0009939_1 | 1 | 0.0 | chr18 | 12873334 | 12873354 | + |
| 316 | miR-100-3p | MI0000692_2 | 1 | 0.0 | chr9 | 41339554 | 41339575 | + |
| 317 | miR-1198-3p | MI0006306_2 | 1 | 0.0 | chrX | 7384306 | 7384327 | + |
| 318 | miR-3110-5p | MI0014107_1 | 1 | 0.0 | chrX | 35563630 | 35563650 | + |
| 319 | miR-1960 | MI0009957_1 | 1 | 0.0 | chr5 | 30497291 | 30497312 | + |
| 320 | miR-701-3p | MI0004685_2 | 1 | 0.0 | chr5 | 111433227 | 111433246 | + |
| 321 | miR-3066-5p | MI0014028_1 | 1 | 0.0 | chr12 | 17362210 | 17362231 | + |
| 322 | miR-3066-3p | MI0014028_2 | 1 | 0.0 | chr12 | 17362247 | 17362268 | + |
| 323 | miR-540-5p | MI0003518_1 | 1 | 0.0 | chr12 | 110824295 | 110824317 | + |
| 324 | miR-666-5p | MI0004553_1 | 1 | 0.0 | chr12 | 110955313 | 110955334 | + |
| 325 | miR-134-3p | MI0000160_2 | 1 | 0.0 | chr12 | 110972394 | 110972413 | + |
| 326 | miR-154-3p | MI0000176_2 | 1 | 0.0 | chr12 | 110976684 | 110976705 | + |
| 327 | miR-3061-5p | MI0014023_1 | 1 | 0.0 | chr11 | 51940261 | 51940282 | + |
| 328 | miR-1934-5p | MI0009923_1 | 1 | 0.0 | chr11 | 69476557 | 69476579 | + |
| 329 | miR-291a-5p | MI0000389_1 | 1 | 0.0 | chr7 | 3218933 | 3218954 | + |
| 330 | miR-295-3p | MI0000393_2 | 1 | 0.0 | chr7 | 3220816 | 3220838 | + |
| 331 | miR-1964-5p | MI0009961_1 | 1 | 0.0 | chr7 | 30558324 | 30558345 | + |
| 332 | miR-150-3p | MI0000172_2 | 1 | 0.0 | chr7 | 52377168 | 52377189 | + |
| 333 | miR-1982.2-3p | MI0009993_3 | 1 | 0.0 | chr10 | 80291595 | 80291615 | + |
| 334 | miR-5615-5p | MI0019182_1 | 1 | 0.0 | chr10 | 80567361 | 80567382 | + |
| 335 | miR-378b | MI0016973_1 | 0 | 0.0 | chr11 | 88166341 | 88166360 | + |
| 336 | miR-491-5p | MI0004680_1 | 0 | 0.0 | chr4 | 87767961 | 87767982 | + |
| 337 | miR-499-5p | MI0004676_1 | 0 | 0.0 | chr2 | 155448629 | 155448649 | + |
| 338 | miR-490-5p | MI0005002_1 | 0 | 0.0 | chr6 | 36371763 | 36371782 | + |
| 339 | miR-365-1-5p | MI0000768_1 | 0 | 0.0 | chr16 | 13453948 | 13453970 | + |
| 340 | miR-3065-5p | MI0014027_1 | 0 | 0.0 | chr11 | 119876094 | 119876115 | + |
| 341 | miR-370-3p | MI0001165_2 | 0 | 0.0 | chr12 | 110856515 | 110856536 | + |
| 342 | miR-3065-3p | MI0014027_2 | 0 | 0.0 | chr11 | 119876133 | 119876155 | + |
| 343 | miR-323-3p | MI0000592_2 | 0 | 0.0 | chr12 | 110950768 | 110950788 | + |
| 344 | miR-3059-5p | MI0004412_1 | 0 | 0.0 | chr10 | 101235338 | 101235359 | + |
| 345 | miR-23a-5p | MI0000571_1 | 0 | 0.0 | chr8 | 86732426 | 86732447 | + |
| 346 | miR-181b-2-3p | MI0000823_2 | 0 | 0.0 | chr2 | 38709403 | 38709424 | + |
| 347 | miR-499-3p | MI0004676_2 | 0 | 0.0 | chr2 | 155448665 | 155448687 | + |
| 348 | miR-124-3p | MI0000150_2 | 0 | 0.0 | chr2 | 180628788 | 180628807 | + |
| 349 | miR-124-3p | MI0000717_2 | 0 | 0.0 | chr3 | 17695723 | 17695742 | + |
| 350 | miR-124-3p | MI0000716_2 | 0 | 0.0 | chr14 | 65209546 | 65209565 | + |
| 351 | miR-218-2-3p | MI0000701_2 | 0 | 0.0 | chr11 | 35430384 | 35430405 | + |
| 352 | miR-497-3p | MI0004636_2 | 0 | 0.0 | chr11 | 70048272 | 70048292 | + |
| 353 | miR-381-5p | MI0000798_1 | 0 | 0.0 | chr12 | 110965039 | 110965061 | + |
| 354 | miR-380-5p | MI0000797_1 | 0 | 0.0 | chr12 | 110950016 | 110950037 | + |
| 355 | miR-122-5p | MI0000256_1 | 0 | 0.0 | chr18 | 65408520 | 65408541 | + |
| 356 | miR-539-5p | MI0003520_1 | 0 | 0.0 | chr12 | 110966346 | 110966367 | + |
| 357 | miR-491-3p | MI0004680_2 | 0 | 0.0 | chr4 | 87767995 | 87768016 | + |
| 358 | miR-103-1-5p | MI0000587_1 | 0 | 0.0 | chr11 | 35595912 | 35595934 | + |
| 359 | miR-135b-3p | MI0000646_2 | 0 | 0.0 | chr1 | 134094719 | 134094740 | + |
| 360 | miR-101b-5p | MI0000649_1 | 0 | 0.0 | chr19 | 29209792 | 29209814 | + |
| 361 | miR-138-2-3p | MI0000164_2 | 0 | 0.0 | chr8 | 96848259 | 96848279 | + |
| 362 | miR-374c-5p | MI0014108_1 | 0 | 0.0 | chrX | 100768425 | 100768444 | + |
| 363 | miR-323-5p | MI0000592_1 | 0 | 0.0 | chr12 | 110950733 | 110950754 | + |
| 364 | miR-485-3p | MI0003492_2 | 0 | 0.0 | chr12 | 110973156 | 110973177 | + |
| 365 | miR-466m-5p | MI0014050_1 | 0 | 0.0 | chr2 | 10388304 | 10388326 | + |
| 366 | miR-466m-3p | MI0014050_2 | 0 | 0.0 | chr2 | 10388340 | 10388361 | + |
| 367 | miR-466f-5p | MI0005507_1 | 0 | 0.0 | chr2 | 10388581 | 10388602 | + |
| 368 | miR-466f-3p | MI0005507_2 | 0 | 0.0 | chr2 | 10388625 | 10388645 | + |
| 369 | miR-669f-3p | MI0006287_2 | 0 | 0.0 | chr2 | 10388912 | 10388934 | + |
| 370 | miR-669e-5p | MI0006300_1 | 0 | 0.0 | chr2 | 10389164 | 10389185 | + |
| 371 | miR-669e-3p | MI0006300_2 | 0 | 0.0 | chr2 | 10389201 | 10389221 | + |
| 372 | miR-669b-3p | MI0004666_2 | 0 | 0.0 | chr2 | 10389478 | 10389500 | + |
| 373 | miR-669d-3p | MI0006281_2 | 0 | 0.0 | chr2 | 10390050 | 10390070 | + |
| 374 | miR-466f-5p | MI0005508_1 | 0 | 0.0 | chr2 | 10390312 | 10390333 | + |
| 375 | miR-466f-3p | MI0005508_2 | 0 | 0.0 | chr2 | 10390356 | 10390376 | + |
| 376 | miR-669l-3p | MI0009942_2 | 0 | 0.0 | chr2 | 10390654 | 10390675 | + |
| 377 | miR-669d-2-3p | MI0014051_2 | 0 | 0.0 | chr2 | 10393323 | 10393344 | + |
| 378 | miR-466f-5p | MI0005509_1 | 0 | 0.0 | chr2 | 10393590 | 10393611 | + |
| 379 | miR-466f-3p | MI0005509_2 | 0 | 0.0 | chr2 | 10393634 | 10393654 | + |
| 380 | miR-297a-5p | MI0000397_1 | 0 | 0.0 | chr2 | 10393891 | 10393912 | + |
| 381 | miR-466o-5p | MI0014052_1 | 0 | 0.0 | chr2 | 10394180 | 10394201 | + |
| 382 | miR-466o-3p | MI0014052_2 | 0 | 0.0 | chr2 | 10394216 | 10394237 | + |
| 383 | miR-467c-3p | MI0005512_2 | 0 | 0.0 | chr2 | 10395604 | 10395625 | + |
| 384 | miR-466b-5p | MI0005502_1 | 0 | 0.0 | chr2 | 10395855 | 10395876 | + |
| 385 | miR-466b-3p | MI0005502_2 | 0 | 0.0 | chr2 | 10395896 | 10395917 | + |
| 386 | miR-669a-5p | MI0004668_1 | 0 | 0.0 | chr2 | 10396087 | 10396110 | + |
| 387 | miR-669a-3-3p | MI0004668_2 | 0 | 0.0 | chr2 | 10396123 | 10396145 | + |
| 388 | miR-669k-5p | MI0006279_1 | 0 | 0.0 | chr2 | 10396945 | 10396969 | + |
| 389 | miR-669k-3p | MI0006279_2 | 0 | 0.0 | chr2 | 10396991 | 10397011 | + |
| 390 | miR-467a-5p | MI0002402_1 | 0 | 0.0 | chr2 | 10397982 | 10398003 | + |
| 391 | miR-467a-3p | MI0002402_2 | 0 | 0.0 | chr2 | 10398017 | 10398038 | + |
| 392 | miR-466b-5p | MI0014112_1 | 0 | 0.0 | chr2 | 10398267 | 10398288 | + |
| 393 | miR-466b-3p | MI0014112_2 | 0 | 0.0 | chr2 | 10398308 | 10398329 | + |
| 394 | miR-669a-5p | MI0004523_1 | 0 | 0.0 | chr2 | 10398499 | 10398522 | + |
| 395 | miR-669a-3p | MI0004523_2 | 0 | 0.0 | chr2 | 10398535 | 10398557 | + |
| 396 | miR-669g | MI0006280_1 | 0 | 0.0 | chr2 | 10398797 | 10398819 | + |
| 397 | miR-669j | MI0006286_1 | 0 | 0.0 | chr2 | 10399616 | 10399637 | + |
| 398 | miR-467a-5p | MI0014053_1 | 0 | 0.0 | chr2 | 10400439 | 10400460 | + |
| 399 | miR-467a-3p | MI0014053_2 | 0 | 0.0 | chr2 | 10400474 | 10400495 | + |
| 400 | miR-466e-5p | MI0005506_1 | 0 | 0.0 | chr2 | 10400726 | 10400747 | + |
| 401 | miR-466e-3p | MI0005506_2 | 0 | 0.0 | chr2 | 10400765 | 10400787 | + |
| 402 | miR-669a-5p | MI0014054_1 | 0 | 0.0 | chr2 | 10400957 | 10400980 | + |
| 403 | miR-669a-3p | MI0014054_2 | 0 | 0.0 | chr2 | 10400993 | 10401015 | + |
| 404 | miR-467b-5p | MI0004671_1 | 0 | 0.0 | chr2 | 10402883 | 10402903 | + |
| 405 | miR-467b-3p | MI0004671_2 | 0 | 0.0 | chr2 | 10402920 | 10402941 | + |
| 406 | miR-466c-5p | MI0005505_1 | 0 | 0.0 | chr2 | 10403171 | 10403194 | + |
| 407 | miR-466c-3p | MI0005505_2 | 0 | 0.0 | chr2 | 10403212 | 10403233 | + |
| 408 | miR-669a-5p | MI0014055_1 | 0 | 0.0 | chr2 | 10403403 | 10403426 | + |
| 409 | miR-669a-3p | MI0014055_2 | 0 | 0.0 | chr2 | 10403439 | 10403461 | + |
| 410 | miR-467a-5p | MI0014056_1 | 0 | 0.0 | chr2 | 10405319 | 10405340 | + |
| 411 | miR-467a-3p | MI0014056_2 | 0 | 0.0 | chr2 | 10405354 | 10405375 | + |
| 412 | miR-466c-5p | MI0014057_1 | 0 | 0.0 | chr2 | 10405606 | 10405629 | + |
| 413 | miR-466c-3p | MI0014057_2 | 0 | 0.0 | chr2 | 10405647 | 10405668 | + |
| 414 | miR-669a-5p | MI0014058_1 | 0 | 0.0 | chr2 | 10405838 | 10405861 | + |
| 415 | miR-669a-3p | MI0014058_2 | 0 | 0.0 | chr2 | 10405874 | 10405896 | + |
| 416 | miR-467a-5p | MI0014059_1 | 0 | 0.0 | chr2 | 10407776 | 10407797 | + |
| 417 | miR-467a-3p | MI0014059_2 | 0 | 0.0 | chr2 | 10407811 | 10407832 | + |
| 418 | miR-466b-5p | MI0014060_1 | 0 | 0.0 | chr2 | 10408063 | 10408084 | + |
| 419 | miR-466b-3p | MI0014060_2 | 0 | 0.0 | chr2 | 10408104 | 10408125 | + |
| 420 | miR-669a-5p | MI0014061_1 | 0 | 0.0 | chr2 | 10408295 | 10408318 | + |
| 421 | miR-669a-3p | MI0014061_2 | 0 | 0.0 | chr2 | 10408331 | 10408353 | + |
| 422 | miR-467a-5p | MI0014062_1 | 0 | 0.0 | chr2 | 10410240 | 10410261 | + |
| 423 | miR-467a-3p | MI0014062_2 | 0 | 0.0 | chr2 | 10410275 | 10410296 | + |
| 424 | miR-466b-5p | MI0014063_1 | 0 | 0.0 | chr2 | 10410525 | 10410546 | + |
| 425 | miR-466b-3p | MI0014063_2 | 0 | 0.0 | chr2 | 10410566 | 10410587 | + |
| 426 | miR-669p-5p | MI0014064_1 | 0 | 0.0 | chr2 | 10410757 | 10410780 | + |
| 427 | miR-669p-3p | MI0014064_2 | 0 | 0.0 | chr2 | 10410794 | 10410817 | + |
| 428 | miR-467a-5p | MI0014065_1 | 0 | 0.0 | chr2 | 10412689 | 10412710 | + |
| 429 | miR-467a-3p | MI0014065_2 | 0 | 0.0 | chr2 | 10412724 | 10412745 | + |
| 430 | miR-669a-5p | MI0014066_1 | 0 | 0.0 | chr2 | 10413208 | 10413231 | + |
| 431 | miR-669a-3p | MI0014066_2 | 0 | 0.0 | chr2 | 10413244 | 10413266 | + |
| 432 | miR-467a-5p | MI0014069_1 | 0 | 0.0 | chr2 | 10415151 | 10415172 | + |
| 433 | miR-467a-3p | MI0014069_2 | 0 | 0.0 | chr2 | 10415186 | 10415207 | + |
| 434 | miR-466b-5p | MI0014067_1 | 0 | 0.0 | chr2 | 10415438 | 10415459 | + |
| 435 | miR-466b-3p | MI0014067_2 | 0 | 0.0 | chr2 | 10415479 | 10415500 | + |
| 436 | miR-669a-5p | MI0014068_1 | 0 | 0.0 | chr2 | 10415670 | 10415693 | + |
| 437 | miR-669a-3p | MI0014068_2 | 0 | 0.0 | chr2 | 10415706 | 10415728 | + |
| 438 | miR-467a-5p | MI0014072_1 | 0 | 0.0 | chr2 | 10417621 | 10417642 | + |
| 439 | miR-467a-3p | MI0014072_2 | 0 | 0.0 | chr2 | 10417656 | 10417677 | + |
| 440 | miR-466b-5p | MI0014070_1 | 0 | 0.0 | chr2 | 10417906 | 10417927 | + |
| 441 | miR-466b-3p | MI0014070_2 | 0 | 0.0 | chr2 | 10417947 | 10417968 | + |
| 442 | miR-669p-5p | MI0014071_1 | 0 | 0.0 | chr2 | 10418138 | 10418161 | + |
| 443 | miR-669p-3p | MI0014071_2 | 0 | 0.0 | chr2 | 10418175 | 10418198 | + |
| 444 | miR-467a-5p | MI0014074_1 | 0 | 0.0 | chr2 | 10420034 | 10420055 | + |
| 445 | miR-467a-3p | MI0014074_2 | 0 | 0.0 | chr2 | 10420069 | 10420090 | + |
| 446 | miR-466b-5p | MI0005503_1 | 0 | 0.0 | chr2 | 10420321 | 10420342 | + |
| 447 | miR-466b-3p | MI0005503_2 | 0 | 0.0 | chr2 | 10420362 | 10420383 | + |
| 448 | miR-669a-5p | MI0014073_1 | 0 | 0.0 | chr2 | 10420553 | 10420576 | + |
| 449 | miR-669a-3p | MI0014073_2 | 0 | 0.0 | chr2 | 10420589 | 10420611 | + |
| 450 | miR-669a-5p | MI0014075_1 | 0 | 0.0 | chr2 | 10422981 | 10423004 | + |
| 451 | miR-669a-3p | MI0014075_2 | 0 | 0.0 | chr2 | 10423017 | 10423039 | + |
| 452 | miR-467a-5p | MI0014076_1 | 0 | 0.0 | chr2 | 10424914 | 10424935 | + |
| 453 | miR-467a-3p | MI0014076_2 | 0 | 0.0 | chr2 | 10424949 | 10424970 | + |
| 454 | miR-466b-5p | MI0005504_1 | 0 | 0.0 | chr2 | 10425201 | 10425222 | + |
| 455 | miR-466b-3p | MI0005504_2 | 0 | 0.0 | chr2 | 10425242 | 10425263 | + |
| 456 | miR-669a-5p | MI0014077_1 | 0 | 0.0 | chr2 | 10425433 | 10425456 | + |
| 457 | miR-669a-3p | MI0014077_2 | 0 | 0.0 | chr2 | 10425469 | 10425491 | + |
| 458 | miR-466p-5p | MI0014078_1 | 0 | 0.0 | chr2 | 10427647 | 10427667 | + |
| 459 | miR-466p-3p | MI0014078_2 | 0 | 0.0 | chr2 | 10427686 | 10427707 | + |
| 460 | miR-467d-3p | MI0005513_2 | 0 | 0.0 | chr2 | 10429303 | 10429324 | + |
| 461 | miR-466a-5p | MI0002401_1 | 0 | 0.0 | chr2 | 10429555 | 10429576 | + |
| 462 | miR-466a-3p | MI0002401_2 | 0 | 0.0 | chr2 | 10429594 | 10429616 | + |
| 463 | miR-297c-5p | MI0005492_1 | 0 | 0.0 | chr2 | 10430653 | 10430674 | + |
| 464 | miR-297c-3p | MI0005492_2 | 0 | 0.0 | chr2 | 10430692 | 10430713 | + |
| 465 | miR-669c-3p | MI0004673_2 | 0 | 0.0 | chr2 | 10430986 | 10431007 | + |
| 466 | miR-669a-5p | MI0004667_1 | 0 | 0.0 | chr2 | 10431812 | 10431835 | + |
| 467 | miR-669a-3p | MI0004667_2 | 0 | 0.0 | chr2 | 10431848 | 10431870 | + |
| 468 | miR-297b-3p | MI0004674_2 | 0 | 0.0 | chr2 | 10433350 | 10433371 | + |
| 469 | miR-466d-5p | MI0005546_1 | 0 | 0.0 | chr2 | 10433604 | 10433625 | + |
| 470 | miR-669m-5p | MI0009943_1 | 0 | 0.0 | chr2 | 10434441 | 10434463 | + |
| 471 | miR-669m-3p | MI0009943_2 | 0 | 0.0 | chr2 | 10434474 | 10434495 | + |
| 472 | miR-669m-5p | MI0009944_1 | 0 | 0.0 | chr2 | 10435084 | 10435106 | + |
| 473 | miR-669m-3p | MI0009944_2 | 0 | 0.0 | chr2 | 10435117 | 10435138 | + |
| 474 | miR-466n-5p | MI0014079_1 | 0 | 0.0 | chr2 | 10435386 | 10435407 | + |
| 475 | miR-466n-3p | MI0014079_2 | 0 | 0.0 | chr2 | 10435425 | 10435446 | + |
| 476 | miR-669o-5p | MI0009945_1 | 0 | 0.0 | chr2 | 10435945 | 10435967 | + |
| 477 | miR-669o-3p | MI0009945_2 | 0 | 0.0 | chr2 | 10435982 | 10436004 | + |
| 478 | miR-466g | MI0005510_1 | 0 | 0.0 | chr2 | 10436269 | 10436289 | + |
| 479 | miR-466h-5p | MI0005511_1 | 0 | 0.0 | chr2 | 10436528 | 10436549 | + |
| 480 | miR-466h-3p | MI0005511_2 | 0 | 0.0 | chr2 | 10436567 | 10436585 | + |
| 481 | miR-297a-5p | MI0005488_1 | 0 | 0.0 | chr2 | 10437457 | 10437478 | + |
| 482 | miR-297a-3p | MI0005488_2 | 0 | 0.0 | chr2 | 10437496 | 10437517 | + |
| 483 | miR-466l-5p | MI0006278_1 | 0 | 0.0 | chr2 | 10437744 | 10437765 | + |
| 484 | miR-466l-3p | MI0006278_2 | 0 | 0.0 | chr2 | 10437783 | 10437804 | + |
| 485 | miR-297a-5p | MI0005489_1 | 0 | 0.0 | chr2 | 10438704 | 10438725 | + |
| 486 | miR-297a-3p | MI0005489_2 | 0 | 0.0 | chr2 | 10438743 | 10438764 | + |
| 487 | miR-669i | MI0006288_1 | 0 | 0.0 | chr2 | 10439298 | 10439318 | + |
| 488 | miR-669h-5p | MI0006289_1 | 0 | 0.0 | chr2 | 10439801 | 10439824 | + |
| 489 | miR-669h-3p | MI0006289_2 | 0 | 0.0 | chr2 | 10439846 | 10439867 | + |
| 490 | miR-511-5p | MI0005554_1 | 0 | 0.0 | chr2 | 14182640 | 14182660 | + |
| 491 | miR-3967 | MI0016976_1 | 0 | 0.0 | chr2 | 22509841 | 22509860 | + |
| 492 | miR-3087-5p | MI0014080_1 | 0 | 0.0 | chr2 | 25298300 | 25298320 | + |
| 493 | miR-3087-3p | MI0014080_2 | 0 | 0.0 | chr2 | 25298333 | 25298353 | + |
| 494 | miR-3088-5p | MI0014081_1 | 0 | 0.0 | chr2 | 28588806 | 28588827 | + |
| 495 | miR-3088-3p | MI0014081_2 | 0 | 0.0 | chr2 | 28588841 | 28588863 | + |
| 496 | miR-219-2-3p | MI0000741_1 | 0 | 0.0 | chr2 | 29701165 | 29701186 | - |
| 497 | miR-219-5p | MI0000741_2 | 0 | 0.0 | chr2 | 29701209 | 29701229 | - |
| 498 | miR-3089-5p | MI0014082_1 | 0 | 0.0 | chr2 | 30576742 | 30576763 | + |
| 499 | miR-2861 | MI0013007_1 | 0 | 0.0 | chr2 | 32568339 | 32568357 | - |
| 500 | miR-3960 | MI0016963_1 | 0 | 0.0 | chr2 | 32568427 | 32568446 | - |
| 501 | miR-5128 | MI0018040_1 | 0 | 0.0 | chr2 | 37543732 | 37543754 | - |
| 502 | miR-181a-2-3p | MI0000223_2 | 0 | 0.0 | chr2 | 38708308 | 38708329 | + |
| 503 | miR-5129 | MI0018041_1 | 0 | 0.0 | chr2 | 44878666 | 44878687 | - |
| 504 | miR-5115 | MI0018024_1 | 0 | 0.0 | chr2 | 72850950 | 72850968 | - |
| 505 | miR-684 | MI0004647_1 | 0 | 0.0 | chr2 | 80468871 | 80468889 | - |
| 506 | miR-130a-3p | MI0000156_1 | 0 | 0.0 | chr2 | 84581273 | 84581294 | - |
| 507 | miR-130a-5p | MI0000156_2 | 0 | 0.0 | chr2 | 84581312 | 84581333 | - |
| 508 | miR-1955-5p | MI0009950_1 | 0 | 0.0 | chr2 | 92032151 | 92032175 | + |
| 509 | miR-129-2-3p | MI0000585_1 | 0 | 0.0 | chr2 | 94081533 | 94081554 | - |
| 510 | miR-129-5p | MI0000585_2 | 0 | 0.0 | chr2 | 94081576 | 94081596 | - |
| 511 | miR-670-3p | MI0004295_1 | 0 | 0.0 | chr2 | 94101473 | 94101497 | - |
| 512 | miR-670-5p | MI0004295_2 | 0 | 0.0 | chr2 | 94101515 | 94101536 | - |
| 513 | miR-1902 | MI0008313_1 | 0 | 0.0 | chr2 | 104269028 | 104269049 | - |
| 514 | miR-1951 | MI0009946_1 | 0 | 0.0 | chr2 | 115464513 | 115464534 | + |
| 515 | miR-147-5p | MI0005482_1 | 0 | 0.0 | chr2 | 122466550 | 122466573 | + |
| 516 | miR-3090-5p | MI0014083_1 | 0 | 0.0 | chr2 | 133390458 | 133390478 | + |
| 517 | miR-1952 | MI0009947_1 | 0 | 0.0 | chr2 | 138645679 | 138645695 | + |
| 518 | miR-1953 | MI0009948_1 | 0 | 0.0 | chr2 | 151793317 | 151793338 | - |
| 519 | miR-5622-5p | MI0019190_1 | 0 | 0.0 | chr2 | 152690803 | 152690824 | + |
| 520 | miR-5622-3p | MI0019190_2 | 0 | 0.0 | chr2 | 152690841 | 152690862 | + |
| 521 | miR-695 | MI0004675_1 | 0 | 0.0 | chr2 | 155182567 | 155182588 | + |
| 522 | miR-5111-3p | MI0018020_1 | 0 | 0.0 | chr2 | 156959654 | 156959672 | - |
| 523 | miR-5111-5p | MI0018020_2 | 0 | 0.0 | chr2 | 156959681 | 156959696 | - |
| 524 | miR-3474 | MI0014707_1 | 0 | 0.0 | chr2 | 158464319 | 158464340 | + |
| 525 | miR-296-3p | MI0000394_1 | 0 | 0.0 | chr2 | 174092559 | 174092580 | - |
| 526 | miR-296-5p | MI0000394_2 | 0 | 0.0 | chr2 | 174092594 | 174092614 | - |
| 527 | miR-298-3p | MI0000398_1 | 0 | 0.0 | chr2 | 174093015 | 174093036 | - |
| 528 | miR-298-5p | MI0000398_2 | 0 | 0.0 | chr2 | 174093054 | 174093076 | - |
| 529 | miR-3091-5p | MI0014084_1 | 0 | 0.0 | chr2 | 179992252 | 179992272 | + |
| 530 | miR-3091-3p | MI0014084_2 | 0 | 0.0 | chr2 | 179992285 | 179992306 | + |
| 531 | miR-1a-1-5p | MI0000139_1 | 0 | 0.0 | chr2 | 180123762 | 180123784 | + |
| 532 | miR-124-5p | MI0000150_1 | 0 | 0.0 | chr2 | 180628750 | 180628771 | + |
| 533 | miR-124-5p | MI0000717_1 | 0 | 0.0 | chr3 | 17695686 | 17695707 | + |
| 534 | miR-3092-3p | MI0014085_1 | 0 | 0.0 | chr3 | 27483837 | 27483857 | - |
| 535 | miR-3092-5p | MI0014085_2 | 0 | 0.0 | chr3 | 27483880 | 27483901 | - |
| 536 | miR-466q | MI0018032_1 | 0 | 0.0 | chr3 | 28318910 | 28318929 | + |
| 537 | miR-551b-5p | MI0004131_1 | 0 | 0.0 | chr3 | 29315762 | 29315784 | + |
| 538 | miR-1897-5p | MI0008314_1 | 0 | 0.0 | chr3 | 34537396 | 34537417 | + |
| 539 | miR-1897-3p | MI0008314_2 | 0 | 0.0 | chr3 | 34537433 | 34537454 | + |
| 540 | miR-466k | MI0006292_1 | 0 | 0.0 | chr3 | 85271318 | 85271340 | + |
| 541 | miR-3093-5p | MI0014086_1 | 0 | 0.0 | chr3 | 88019108 | 88019129 | + |
| 542 | miR-3093-3p | MI0014086_2 | 0 | 0.0 | chr3 | 88019148 | 88019169 | + |
| 543 | miR-1905 | MI0008315_1 | 0 | 0.0 | chr3 | 88340233 | 88340254 | - |
| 544 | miR-720 | MI0004678_1 | 0 | 0.0 | chr3 | 88920532 | 88920549 | - |
| 545 | miR-92b-3p | MI0005521_1 | 0 | 0.0 | chr3 | 89031048 | 89031069 | - |
| 546 | miR-92b-5p | MI0005521_2 | 0 | 0.0 | chr3 | 89031087 | 89031110 | - |
| 547 | miR-190b-3p | MI0005478_2 | 0 | 0.0 | chr3 | 89873989 | 89874011 | + |
| 548 | miR-669n | MI0009951_1 | 0 | 0.0 | chr3 | 115682823 | 115682842 | + |
| 549 | miR-137-5p | MI0000163_1 | 0 | 0.0 | chr3 | 118136783 | 118136805 | + |
| 550 | miR-137-3p | MI0000163_2 | 0 | 0.0 | chr3 | 118136819 | 118136841 | + |
| 551 | miR-760-3p | MI0004605_1 | 0 | 0.0 | chr3 | 121996533 | 121996552 | - |
| 552 | miR-760-5p | MI0004605_2 | 0 | 0.0 | chr3 | 121996567 | 121996589 | - |
| 553 | miR-302b-5p | MI0003716_1 | 0 | 0.0 | chr3 | 127248156 | 127248178 | + |
| 554 | miR-302b-3p | MI0003716_2 | 0 | 0.0 | chr3 | 127248193 | 127248215 | + |
| 555 | miR-302c-5p | MI0003717_1 | 0 | 0.0 | chr3 | 127248286 | 127248307 | + |
| 556 | miR-302c-3p | MI0003717_2 | 0 | 0.0 | chr3 | 127248324 | 127248345 | + |
| 557 | miR-302a-5p | MI0000402_1 | 0 | 0.0 | chr3 | 127248419 | 127248440 | + |
| 558 | miR-302a-3p | MI0000402_2 | 0 | 0.0 | chr3 | 127248457 | 127248479 | + |
| 559 | miR-302d-5p | MI0003718_1 | 0 | 0.0 | chr3 | 127248547 | 127248569 | + |
| 560 | miR-302d-3p | MI0003718_2 | 0 | 0.0 | chr3 | 127248583 | 127248605 | + |
| 561 | miR-367-5p | MI0003531_1 | 0 | 0.0 | chr3 | 127248659 | 127248679 | + |
| 562 | miR-367-3p | MI0003531_2 | 0 | 0.0 | chr3 | 127248697 | 127248718 | + |
| 563 | miR-1895 | MI0008316_1 | 0 | 0.0 | chr3 | 133903479 | 133903500 | - |
| 564 | miR-1956 | MI0009952_1 | 0 | 0.0 | chr3 | 138189389 | 138189410 | + |
| 565 | miR-3963 | MI0016968_1 | 0 | 0.0 | chr3 | 150686803 | 150686821 | - |
| 566 | miR-206-5p | MI0000249_1 | 0 | 0.0 | chr1 | 20669098 | 20669120 | + |
| 567 | miR-206-3p | MI0000249_2 | 0 | 0.0 | chr1 | 20669136 | 20669157 | + |
| 568 | miR-133b-5p | MI0000821_1 | 0 | 0.0 | chr1 | 20672878 | 20672899 | + |
| 569 | miR-5103 | MI0018011_1 | 0 | 0.0 | chr1 | 34489975 | 34489994 | - |
| 570 | miR-1928 | MI0009917_1 | 0 | 0.0 | chr1 | 74253136 | 74253152 | - |
| 571 | miR-375-3p | MI0000792_1 | 0 | 0.0 | chr1 | 74947235 | 74947256 | - |
| 572 | miR-375-5p | MI0000792_2 | 0 | 0.0 | chr1 | 74947270 | 74947291 | - |
| 573 | miR-5126 | MI0018038_1 | 0 | 0.0 | chr1 | 84692465 | 84692486 | + |
| 574 | miR-149-3p | MI0000171_2 | 0 | 0.0 | chr1 | 94746998 | 94747019 | + |
| 575 | miR-1231-3p | MI0019183_1 | 0 | 0.0 | chr1 | 137351180 | 137351202 | - |
| 576 | miR-1231-5p | MI0019183_2 | 0 | 0.0 | chr1 | 137351238 | 137351260 | - |
| 577 | miR-488-5p | MI0004633_1 | 0 | 0.0 | chr1 | 160435782 | 160435802 | + |
| 578 | miR-1843b-3p | MI0016971_2 | 0 | 0.0 | chr1 | 161270531 | 161270550 | + |
| 579 | miR-1927 | MI0009914_1 | 0 | 0.0 | chr1 | 162226016 | 162226038 | - |
| 580 | miR-350-3p | MI0000640_1 | 0 | 0.0 | chr1 | 178702473 | 178702494 | - |
| 581 | miR-350-5p | MI0000640_2 | 0 | 0.0 | chr1 | 178702513 | 178702531 | - |
| 582 | miR-1981-3p | MI0009992_1 | 0 | 0.0 | chr1 | 186646288 | 186646308 | - |
| 583 | miR-1981-5p | MI0009992_2 | 0 | 0.0 | chr1 | 186646344 | 186646367 | - |
| 584 | miR-194-1-3p | MI0000236_2 | 0 | 0.0 | chr1 | 187137239 | 187137260 | + |
| 585 | miR-3473c | MI0018015_1 | 0 | 0.0 | chr1 | 193822483 | 193822504 | - |
| 586 | miR-205-3p | MI0000248_1 | 0 | 0.0 | chr1 | 195333661 | 195333682 | - |
| 587 | miR-205-5p | MI0000248_2 | 0 | 0.0 | chr1 | 195333697 | 195333718 | - |
| 588 | miR-3962 | MI0016967_1 | 0 | 0.0 | chr1 | 196648229 | 196648248 | + |
| 589 | miR-29b-2-5p | MI0000712_1 | 0 | 0.0 | chr1 | 196863244 | 196863268 | + |
| 590 | miR-1945 | MI0009934_1 | 0 | 0.0 | chr16 | 11254474 | 11254495 | - |
| 591 | miR-130b-3p | MI0000408_1 | 0 | 0.0 | chr16 | 17124164 | 17124185 | - |
| 592 | miR-130b-5p | MI0000408_2 | 0 | 0.0 | chr16 | 17124202 | 17124223 | - |
| 593 | miR-301b-3p | MI0004122_1 | 0 | 0.0 | chr16 | 17124513 | 17124535 | - |
| 594 | miR-301b-5p | MI0004122_2 | 0 | 0.0 | chr16 | 17124549 | 17124570 | - |
| 595 | miR-1306-3p | MI0009935_1 | 0 | 0.0 | chr16 | 18284341 | 18284361 | - |
| 596 | miR-1306-5p | MI0009935_2 | 0 | 0.0 | chr16 | 18284381 | 18284403 | - |
| 597 | miR-185-3p | MI0000227_1 | 0 | 0.0 | chr16 | 18327498 | 18327518 | - |
| 598 | miR-185-5p | MI0000227_2 | 0 | 0.0 | chr16 | 18327531 | 18327552 | - |
| 599 | miR-1224-5p | MI0004118_1 | 0 | 0.0 | chr16 | 20604525 | 20604545 | + |
| 600 | miR-1224-3p | MI0004118_2 | 0 | 0.0 | chr16 | 20604589 | 20604609 | + |
| 601 | miR-690 | MI0004658_1 | 0 | 0.0 | chr16 | 28600026 | 28600047 | - |
| 602 | miR-1946a | MI0009936_1 | 0 | 0.0 | chr16 | 32267626 | 32267652 | - |
| 603 | miR-1947-3p | MI0009937_2 | 0 | 0.0 | chr16 | 33105497 | 33105518 | + |
| 604 | miR-568 | MI0005517_1 | 0 | 0.0 | chr16 | 43640778 | 43640797 | + |
| 605 | miR-3470b | MI0014697_1 | 0 | 0.0 | chr16 | 44013998 | 44014018 | + |
| 606 | miR-3081-3p | MI0014044_1 | 0 | 0.0 | chr16 | 44558171 | 44558193 | - |
| 607 | miR-3081-5p | MI0014044_2 | 0 | 0.0 | chr16 | 44558205 | 44558227 | - |
| 608 | miR-5118 | MI0018027_1 | 0 | 0.0 | chr16 | 55494883 | 55494901 | + |
| 609 | miR-691 | MI0004659_1 | 0 | 0.0 | chr16 | 74342250 | 74342271 | - |
| 610 | miR-155-3p | MI0000177_2 | 0 | 0.0 | chr16 | 84714427 | 84714447 | + |
| 611 | miR-1898 | MI0008324_1 | 0 | 0.0 | chr15 | 12101257 | 12101278 | + |
| 612 | miR-3964 | MI0016970_1 | 0 | 0.0 | chr15 | 29643092 | 29643111 | - |
| 613 | miR-599 | MI0012529_1 | 0 | 0.0 | chr15 | 35590601 | 35590619 | - |
| 614 | miR-875-3p | MI0005551_1 | 0 | 0.0 | chr15 | 35590737 | 35590757 | - |
| 615 | miR-875-5p | MI0005551_2 | 0 | 0.0 | chr15 | 35590771 | 35590792 | - |
| 616 | miR-1907 | MI0008325_1 | 0 | 0.0 | chr15 | 50720581 | 50720602 | - |
| 617 | miR-28c | MI0016966_1 | 0 | 0.0 | chr15 | 53445771 | 53445790 | - |
| 618 | miR-30b-3p | MI0000145_1 | 0 | 0.0 | chr15 | 68168993 | 68169014 | - |
| 619 | miR-30b-5p | MI0000145_2 | 0 | 0.0 | chr15 | 68169031 | 68169052 | - |
| 620 | miR-30d-3p | MI0000549_1 | 0 | 0.0 | chr15 | 68172779 | 68172800 | - |
| 621 | miR-30d-5p | MI0000549_2 | 0 | 0.0 | chr15 | 68172819 | 68172840 | - |
| 622 | miR-151-3p | MI0000173_1 | 0 | 0.0 | chr15 | 73085250 | 73085270 | - |
| 623 | miR-151-5p | MI0000173_2 | 0 | 0.0 | chr15 | 73085285 | 73085305 | - |
| 624 | miR-1942 | MI0009931_1 | 0 | 0.0 | chr15 | 76046051 | 76046071 | - |
| 625 | miR-3079-3p | MI0014042_1 | 0 | 0.0 | chr15 | 76121321 | 76121341 | - |
| 626 | miR-3079-5p | MI0014042_2 | 0 | 0.0 | chr15 | 76121362 | 76121384 | - |
| 627 | miR-1943-3p | MI0009932_1 | 0 | 0.0 | chr15 | 79205666 | 79205686 | - |
| 628 | miR-1943-5p | MI0009932_2 | 0 | 0.0 | chr15 | 79205701 | 79205723 | - |
| 629 | miR-5113 | MI0018022_1 | 0 | 0.0 | chr15 | 80770479 | 80770501 | + |
| 630 | miR-3080-3p | MI0014043_1 | 0 | 0.0 | chr15 | 82789658 | 82789679 | - |
| 631 | miR-3080-5p | MI0014043_2 | 0 | 0.0 | chr15 | 82789696 | 82789715 | - |
| 632 | miR-1249-3p | MI0004132_1 | 0 | 0.0 | chr15 | 84781975 | 84781996 | - |
| 633 | miR-1249-5p | MI0004132_2 | 0 | 0.0 | chr15 | 84782009 | 84782033 | - |
| 634 | miR-1941-5p | MI0009930_1 | 0 | 0.0 | chr15 | 101199795 | 101199818 | + |
| 635 | miR-688 | MI0004653_1 | 0 | 0.0 | chr15 | 102502233 | 102502252 | - |
| 636 | miR-3075-3p | MI0014038_2 | 0 | 0.0 | chr14 | 26353977 | 26353997 | + |
| 637 | miR-3076-5p | MI0014039_1 | 0 | 0.0 | chr14 | 31385336 | 31385359 | + |
| 638 | miR-346-3p | MI0000634_2 | 0 | 0.0 | chr14 | 35707848 | 35707869 | + |
| 639 | miR-327 | MI0005493_1 | 0 | 0.0 | chr14 | 45567158 | 45567176 | - |
| 640 | miR-5131 | MI0018043_1 | 0 | 0.0 | chr14 | 46277647 | 46277669 | - |
| 641 | miR-686 | MI0004650_1 | 0 | 0.0 | chr14 | 55235583 | 55235604 | - |
| 642 | miR-208a-3p | MI0000555_1 | 0 | 0.0 | chr14 | 55567909 | 55567930 | - |
| 643 | miR-208a-5p | MI0000555_2 | 0 | 0.0 | chr14 | 55567944 | 55567965 | - |
| 644 | miR-208b-3p | MI0005552_1 | 0 | 0.0 | chr14 | 55594547 | 55594568 | - |
| 645 | miR-208b-5p | MI0005552_2 | 0 | 0.0 | chr14 | 55594582 | 55594603 | - |
| 646 | miR-3077-5p | MI0014040_1 | 0 | 0.0 | chr14 | 58417261 | 58417285 | + |
| 647 | miR-3077-3p | MI0014040_2 | 0 | 0.0 | chr14 | 58417303 | 58417324 | + |
| 648 | miR-719 | MI0004651_1 | 0 | 0.0 | chr14 | 60847710 | 60847731 | - |
| 649 | miR-16-1-3p | MI0000565_1 | 0 | 0.0 | chr14 | 62250731 | 62250752 | - |
| 650 | miR-16-5p | MI0000565_2 | 0 | 0.0 | chr14 | 62250773 | 62250794 | - |
| 651 | miR-15a-3p | MI0000564_1 | 0 | 0.0 | chr14 | 62250875 | 62250896 | - |
| 652 | miR-15a-5p | MI0000564_2 | 0 | 0.0 | chr14 | 62250912 | 62250933 | - |
| 653 | miR-1196-3p | MI0006304_1 | 0 | 0.0 | chr14 | 62371060 | 62371075 | - |
| 654 | miR-1196-5p | MI0006304_2 | 0 | 0.0 | chr14 | 62371136 | 62371155 | - |
| 655 | miR-598-5p | MI0005556_1 | 0 | 0.0 | chr14 | 64346036 | 64346058 | + |
| 656 | miR-124-5p | MI0000716_1 | 0 | 0.0 | chr14 | 65209507 | 65209528 | + |
| 657 | miR-3078-5p | MI0014041_1 | 0 | 0.0 | chr14 | 65210037 | 65210059 | + |
| 658 | miR-3078-3p | MI0014041_2 | 0 | 0.0 | chr14 | 65210076 | 65210095 | + |
| 659 | miR-320-5p | MI0000704_1 | 0 | 0.0 | chr14 | 70843332 | 70843353 | + |
| 660 | miR-687 | MI0004652_1 | 0 | 0.0 | chr14 | 73606615 | 73606636 | - |
| 661 | miR-1971 | MI0009970_1 | 0 | 0.0 | chr14 | 78591258 | 78591275 | - |
| 662 | miR-759 | MI0004554_1 | 0 | 0.0 | chr14 | 80138267 | 80138288 | + |
| 663 | miR-5130 | MI0018042_1 | 0 | 0.0 | chr14 | 103381825 | 103381848 | - |
| 664 | miR-19a-5p | MI0000688_1 | 0 | 0.0 | chr14 | 115443234 | 115443255 | + |
| 665 | miR-466i-5p | MI0006282_1 | 0 | 0.0 | chr13 | 17839359 | 17839378 | + |
| 666 | miR-466i-3p | MI0006282_2 | 0 | 0.0 | chr13 | 17839386 | 17839407 | + |
| 667 | miR-1896 | MI0008322_1 | 0 | 0.0 | chr13 | 21537078 | 21537099 | + |
| 668 | miR-1983 | MI0009990_1 | 0 | 0.0 | chr13 | 21988799 | 21988819 | - |
| 669 | miR-5124 | MI0018035_1 | 0 | 0.0 | chr13 | 40961170 | 40961189 | + |
| 670 | let-7d-3p | MI0000405_1 | 0 | 0.0 | chr13 | 48631393 | 48631414 | - |
| 671 | let-7d-5p | MI0000405_2 | 0 | 0.0 | chr13 | 48631447 | 48631468 | - |
| 672 | let-7f-1-3p | MI0000562_1 | 0 | 0.0 | chr13 | 48633202 | 48633223 | - |
| 673 | let-7f-5p | MI0000562_2 | 0 | 0.0 | chr13 | 48633258 | 48633279 | - |
| 674 | let-7a-1-3p | MI0000556_1 | 0 | 0.0 | chr13 | 48633557 | 48633578 | - |
| 675 | let-7a-5p | MI0000556_2 | 0 | 0.0 | chr13 | 48633608 | 48633629 | - |
| 676 | miR-683 | MI0004646_1 | 0 | 0.0 | chr13 | 50640013 | 50640033 | - |
| 677 | miR-683 | MI0010690_1 | 0 | 0.0 | chr13 | 50696359 | 50696379 | - |
| 678 | miR-874-3p | MI0005479_1 | 0 | 0.0 | chr13 | 58124496 | 58124517 | - |
| 679 | miR-874-5p | MI0005479_2 | 0 | 0.0 | chr13 | 58124531 | 58124552 | - |
| 680 | miR-7a-1-3p | MI0000728_1 | 0 | 0.0 | chr13 | 58494161 | 58494182 | - |
| 681 | miR-7a-5p | MI0000728_2 | 0 | 0.0 | chr13 | 58494202 | 58494224 | - |
| 682 | miR-713 | MI0004698_1 | 0 | 0.0 | chr13 | 62862167 | 62862185 | + |
| 683 | miR-3074-1-3p | MI0014037_1 | 0 | 0.0 | chr13 | 63402519 | 63402540 | - |
| 684 | miR-3074-5p | MI0014037_2 | 0 | 0.0 | chr13 | 63402557 | 63402578 | - |
| 685 | miR-466f | MI0006291_1 | 0 | 0.0 | chr13 | 71245986 | 71246007 | + |
| 686 | miR-692 | MI0004661_chr13_1 | 0 | 0.0 | chr13 | 74544569 | 74544589 | + |
| 687 | miR-682 | MI0004644_1 | 0 | 0.0 | chr13 | 75782567 | 75782587 | + |
| 688 | miR-3961 | MI0016965_1 | 0 | 0.0 | chr13 | 82837277 | 82837295 | - |
| 689 | miR-5624-5p | MI0019193_1 | 0 | 0.0 | chr13 | 94560732 | 94560749 | + |
| 690 | miR-5624-3p | MI0019193_2 | 0 | 0.0 | chr13 | 94560771 | 94560792 | + |
| 691 | miR-1940 | MI0009929_1 | 0 | 0.0 | chr13 | 96100554 | 96100580 | + |
| 692 | miR-1904 | MI0008323_1 | 0 | 0.0 | chr13 | 110694027 | 110694048 | + |
| 693 | miR-449c-5p | MI0004645_1 | 0 | 0.0 | chr13 | 113826216 | 113826236 | + |
| 694 | miR-449c-3p | MI0004645_2 | 0 | 0.0 | chr13 | 113826257 | 113826278 | + |
| 695 | miR-449b | MI0005547_1 | 0 | 0.0 | chr13 | 113827637 | 113827656 | + |
| 696 | miR-449a-3p | MI0001649_2 | 0 | 0.0 | chr13 | 113827797 | 113827818 | + |
| 697 | miR-5046 | MI0017931_1 | 0 | 0.0 | chr19 | 6904407 | 6904430 | - |
| 698 | miR-5136 | MI0018048_1 | 0 | 0.0 | chr19 | 8963199 | 8963218 | - |
| 699 | miR-1192 | MI0006297_1 | 0 | 0.0 | chr19 | 23223940 | 23223961 | + |
| 700 | miR-3084-3p | MI0014047_1chr19_1 | 0 | 0.0 | chr19 | 25016730 | 25016750 | - |
| 701 | miR-3084-5p | MI0014047_1chr19_2 | 0 | 0.0 | chr19 | 25016766 | 25016787 | - |
| 702 | miR-3970 | MI0016980_1 | 0 | 0.0 | chr19 | 33231612 | 33231631 | + |
| 703 | miR-107-3p | MI0000684_1 | 0 | 0.0 | chr19 | 34895190 | 34895212 | - |
| 704 | miR-107-5p | MI0000684_2 | 0 | 0.0 | chr19 | 34895227 | 34895249 | - |
| 705 | miR-1950 | MI0009941_1 | 0 | 0.0 | chr19 | 35039892 | 35039913 | + |
| 706 | miR-3085-3p | MI0014048_1 | 0 | 0.0 | chr19 | 42354585 | 42354605 | - |
| 707 | miR-3085-5p | MI0014048_2 | 0 | 0.0 | chr19 | 42354621 | 42354645 | - |
| 708 | miR-5114 | MI0018023_1 | 0 | 0.0 | chr19 | 44377661 | 44377682 | + |
| 709 | miR-5623-5p | MI0019191_1 | 0 | 0.0 | chr19 | 58125657 | 58125680 | + |
| 710 | miR-5623-3p | MI0019191_2 | 0 | 0.0 | chr19 | 58125705 | 58125726 | + |
| 711 | miR-3086-3p | MI0014049_1 | 0 | 0.0 | chr19 | 58986176 | 58986197 | - |
| 712 | miR-3086-5p | MI0014049_2 | 0 | 0.0 | chr19 | 58986215 | 58986234 | - |
| 713 | miR-3084-3p | MI0014047_2chr19_1 | 0 | 0.0 | chr19 | 60850237 | 60850257 | - |
| 714 | miR-3084-5p | MI0014047_2chr19_2 | 0 | 0.0 | chr19 | 60850273 | 60850294 | - |
| 715 | miR-1893 | MI0008327_1 | 0 | 0.0 | chr18 | 6490572 | 6490593 | - |
| 716 | miR-133a-3p | MI0000159_1 | 0 | 0.0 | chr18 | 10782911 | 10782932 | - |
| 717 | miR-133a-5p | MI0000159_2 | 0 | 0.0 | chr18 | 10782948 | 10782968 | - |
| 718 | miR-1b-5p | MI0006283_1 | 0 | 0.0 | chr18 | 10785484 | 10785504 | + |
| 719 | miR-1b-3p | MI0006283_2 | 0 | 0.0 | chr18 | 10785523 | 10785545 | + |
| 720 | miR-1a-3p | MI0000652_1 | 0 | 0.0 | chr18 | 10785483 | 10785504 | - |
| 721 | miR-1a-2-5p | MI0000652_2 | 0 | 0.0 | chr18 | 10785521 | 10785543 | - |
| 722 | miR-1901 | MI0008328_1 | 0 | 0.0 | chr18 | 11998880 | 11998901 | - |
| 723 | miR-187-3p | MI0000229_1 | 0 | 0.0 | chr18 | 24587611 | 24587632 | - |
| 724 | miR-187-5p | MI0000229_2 | 0 | 0.0 | chr18 | 24587648 | 24587669 | - |
| 725 | miR-1949 | MI0009940_1 | 0 | 0.0 | chr18 | 35714266 | 35714289 | + |
| 726 | miR-5097 | MI0018005_1 | 0 | 0.0 | chr18 | 41356162 | 41356185 | + |
| 727 | miR-5107 | MI0018016_1 | 0 | 0.0 | chr18 | 60971740 | 60971760 | + |
| 728 | miR-378-3p | MI0000795_1 | 0 | 0.0 | chr18 | 61557492 | 61557512 | - |
| 729 | miR-378-5p | MI0000795_2 | 0 | 0.0 | chr18 | 61557529 | 61557550 | - |
| 730 | miR-145-3p | MI0000169_1 | 0 | 0.0 | chr18 | 61807481 | 61807502 | - |
| 731 | miR-145-5p | MI0000169_2 | 0 | 0.0 | chr18 | 61807520 | 61807542 | - |
| 732 | miR-143-3p | MI0000257_1 | 0 | 0.0 | chr18 | 61808853 | 61808873 | - |
| 733 | miR-143-5p | MI0000257_2 | 0 | 0.0 | chr18 | 61808887 | 61808907 | - |
| 734 | miR-122-3p | MI0000256_2 | 0 | 0.0 | chr18 | 65408555 | 65408574 | + |
| 735 | miR-694 | MI0004664_1 | 0 | 0.0 | chr18 | 66378930 | 66378948 | - |
| 736 | miR-5127 | MI0018039_1 | 0 | 0.0 | chr18 | 82188759 | 82188777 | - |
| 737 | miR-5112 | MI0018021_1 | 0 | 0.0 | chr18 | 82889683 | 82889701 | + |
| 738 | miR-692 | MI0004660_1 | 0 | 0.0 | chr17 | 7099608 | 7099628 | - |
| 739 | miR-5125 | MI0018036_1 | 0 | 0.0 | chr17 | 23960306 | 23960325 | + |
| 740 | miR-5134 | MI0018046_1 | 0 | 0.0 | chr17 | 24371520 | 24371540 | - |
| 741 | miR-3082-3p | MI0014045_1 | 0 | 0.0 | chr17 | 25968311 | 25968332 | - |
| 742 | miR-3082-5p | MI0014045_2 | 0 | 0.0 | chr17 | 25968351 | 25968372 | - |
| 743 | miR-3083-3p | MI0014046_1 | 0 | 0.0 | chr17 | 27085003 | 27085024 | - |
| 744 | miR-3083-5p | MI0014046_2 | 0 | 0.0 | chr17 | 27085038 | 27085059 | - |
| 745 | miR-219-1-3p | MI0000702_1 | 0 | 0.0 | chr17 | 34161955 | 34161976 | - |
| 746 | miR-219-5p | MI0000702_2 | 0 | 0.0 | chr17 | 34161997 | 34162017 | - |
| 747 | miR-1894-5p | MI0008326_1 | 0 | 0.0 | chr17 | 36054844 | 36054865 | + |
| 748 | miR-1894-3p | MI0008326_2 | 0 | 0.0 | chr17 | 36054883 | 36054904 | + |
| 749 | miR-877-3p | MI0005553_1 | 0 | 0.0 | chr17 | 36097676 | 36097697 | - |
| 750 | miR-877-5p | MI0005553_2 | 0 | 0.0 | chr17 | 36097740 | 36097759 | - |
| 751 | miR-693-5p | MI0004662_1 | 0 | 0.0 | chr17 | 46368500 | 46368520 | + |
| 752 | miR-693-3p | MI0004662_2 | 0 | 0.0 | chr17 | 46368530 | 46368552 | + |
| 753 | miR-7b-5p | MI0000730_1 | 0 | 0.0 | chr17 | 56382440 | 56382462 | + |
| 754 | miR-7b-3p | MI0000730_2 | 0 | 0.0 | chr17 | 56382478 | 56382499 | + |
| 755 | miR-5709 | MI0019318_1 | 0 | 0.0 | chr17 | 67375537 | 67375558 | - |
| 756 | miR-1195 | MI0006303_1 | 0 | 0.0 | chr17 | 71209898 | 71209920 | - |
| 757 | miR-101c | MI0016974_1 | 0 | 0.0 | chr9 | 3038680 | 3038698 | - |
| 758 | miR-5618-5p | MI0019186_1 | 0 | 0.0 | chr9 | 7784390 | 7784409 | + |
| 759 | miR-5618-3p | MI0019186_2 | 0 | 0.0 | chr9 | 7784423 | 7784440 | + |
| 760 | miR-1899 | MI0008318_1 | 0 | 0.0 | chr9 | 8246796 | 8246817 | + |
| 761 | miR-1900 | MI0008319_1 | 0 | 0.0 | chr9 | 20836705 | 20836726 | - |
| 762 | miR-199a-3p | MI0000241_1 | 0 | 0.0 | chr9 | 21300942 | 21300963 | - |
| 763 | miR-199a-5p | MI0000241_2 | 0 | 0.0 | chr9 | 21300981 | 21301003 | - |
| 764 | miR-1946b | MI0009967_1 | 0 | 0.0 | chr9 | 21417896 | 21417921 | - |
| 765 | let-7a-2-3p | MI0000557_2 | 0 | 0.0 | chr9 | 41344860 | 41344880 | + |
| 766 | miR-34c-3p | MI0000403_1 | 0 | 0.0 | chr9 | 50911149 | 50911170 | - |
| 767 | miR-34c-5p | MI0000403_2 | 0 | 0.0 | chr9 | 50911181 | 50911203 | - |
| 768 | miR-34b-3p | MI0000404_1 | 0 | 0.0 | chr9 | 50911679 | 50911700 | - |
| 769 | miR-34b-5p | MI0000404_2 | 0 | 0.0 | chr9 | 50911715 | 50911737 | - |
| 770 | miR-5710 | MI0019319_1 | 0 | 0.0 | chr9 | 54556124 | 54556145 | + |
| 771 | miR-5133 | MI0018045_1 | 0 | 0.0 | chr9 | 61970372 | 61970392 | - |
| 772 | miR-190-3p | MI0000232_1 | 0 | 0.0 | chr9 | 67084471 | 67084492 | - |
| 773 | miR-190-5p | MI0000232_2 | 0 | 0.0 | chr9 | 67084507 | 67084528 | - |
| 774 | miR-3109-5p | MI0014106_1 | 0 | 0.0 | chr9 | 69304765 | 69304788 | + |
| 775 | miR-5626-3p | MI0019195_1 | 0 | 0.0 | chr9 | 70253501 | 70253522 | - |
| 776 | miR-5626-5p | MI0019195_2 | 0 | 0.0 | chr9 | 70253539 | 70253560 | - |
| 777 | miR-184-3p | MI0000226_1 | 0 | 0.0 | chr9 | 89697101 | 89697122 | - |
| 778 | miR-184-5p | MI0000226_2 | 0 | 0.0 | chr9 | 89697140 | 89697161 | - |
| 779 | miR-2136 | MI0010749_1 | 0 | 0.0 | chr9 | 104328493 | 104328513 | + |
| 780 | miR-135a-1-3p | MI0000161_2 | 0 | 0.0 | chr9 | 106056510 | 106056531 | + |
| 781 | miR-711 | MI0004695_1 | 0 | 0.0 | chr9 | 108872015 | 108872036 | + |
| 782 | miR-128-3p | MI0000726_1 | 0 | 0.0 | chr9 | 112021148 | 112021168 | - |
| 783 | miR-128-2-5p | MI0000726_2 | 0 | 0.0 | chr9 | 112021183 | 112021205 | - |
| 784 | miR-467h | MI0006302_1 | 0 | 0.0 | chr9 | 115291016 | 115291037 | + |
| 785 | miR-138-1-3p | MI0000722_2 | 0 | 0.0 | chr9 | 122592054 | 122592075 | + |
| 786 | miR-1968-3p | MI0009965_1 | 0 | 0.0 | chr8 | 13189035 | 13189055 | - |
| 787 | miR-1968-5p | MI0009965_2 | 0 | 0.0 | chr8 | 13189068 | 13189091 | - |
| 788 | miR-3106-3p | MI0004486_1 | 0 | 0.0 | chr8 | 16168768 | 16168789 | - |
| 789 | miR-3106-5p | MI0004486_2 | 0 | 0.0 | chr8 | 16168805 | 16168825 | - |
| 790 | miR-3107-3p | MI0014103_1 | 0 | 0.0 | chr8 | 24253059 | 24253078 | - |
| 791 | miR-3107-5p | MI0014103_2 | 0 | 0.0 | chr8 | 24253099 | 24253120 | - |
| 792 | miR-1186 | MI0006284_1 | 0 | 0.0 | chr8 | 32213473 | 32213494 | - |
| 793 | miR-383-3p | MI0000800_1 | 0 | 0.0 | chr8 | 39315192 | 39315212 | - |
| 794 | miR-383-5p | MI0000800_2 | 0 | 0.0 | chr8 | 39315232 | 39315253 | - |
| 795 | miR-710 | MI0004694_1 | 0 | 0.0 | chr8 | 66993198 | 66993219 | - |
| 796 | miR-1969 | MI0009966_1 | 0 | 0.0 | chr8 | 73449440 | 73449462 | + |
| 797 | miR-28b | MI0016979_1 | 0 | 0.0 | chr8 | 75540422 | 75540442 | + |
| 798 | miR-1199-3p | MI0006307_1 | 0 | 0.0 | chr8 | 86535448 | 86535468 | - |
| 799 | miR-1199-5p | MI0006307_2 | 0 | 0.0 | chr8 | 86535493 | 86535512 | - |
| 800 | miR-709 | MI0004693_1 | 0 | 0.0 | chr8 | 86610066 | 86610084 | + |
| 801 | miR-181d-3p | MI0005450_1 | 0 | 0.0 | chr8 | 86702619 | 86702639 | - |
| 802 | miR-181d-5p | MI0005450_2 | 0 | 0.0 | chr8 | 86702658 | 86702680 | - |
| 803 | miR-181c-3p | MI0000724_1 | 0 | 0.0 | chr8 | 86702784 | 86702805 | - |
| 804 | miR-181c-5p | MI0000724_2 | 0 | 0.0 | chr8 | 86702823 | 86702844 | - |
| 805 | miR-3074-2-3p | MI0014104_1 | 0 | 0.0 | chr8 | 86732738 | 86732757 | - |
| 806 | miR-3074-5p | MI0014104_2 | 0 | 0.0 | chr8 | 86732772 | 86732793 | - |
| 807 | miR-1186b | MI0014705_1 | 0 | 0.0 | chr8 | 98487444 | 98487465 | + |
| 808 | miR-328-3p | MI0000603_1 | 0 | 0.0 | chr8 | 107832279 | 107832300 | - |
| 809 | miR-328-5p | MI0000603_2 | 0 | 0.0 | chr8 | 107832319 | 107832340 | - |
| 810 | miR-1966 | MI0009963_1 | 0 | 0.0 | chr8 | 108139381 | 108139405 | + |
| 811 | miR-3108-5p | MI0014105_1 | 0 | 0.0 | chr8 | 111460770 | 111460792 | + |
| 812 | miR-3108-3p | MI0014105_2 | 0 | 0.0 | chr8 | 111460811 | 111460831 | + |
| 813 | miR-3473d | MI0018033_1 | 0 | 0.0 | chr8 | 113540398 | 113540421 | - |
| 814 | miR-1967 | MI0009964_1 | 0 | 0.0 | chr8 | 126546588 | 126546610 | + |
| 815 | miR-1903 | MI0008317_1 | 0 | 0.0 | chr8 | 130883151 | 130883172 | + |
| 816 | miR-500-3p | MI0004702_1 | 0 | 0.0 | chrX | 6814821 | 6814842 | - |
| 817 | miR-500-5p | MI0004702_2 | 0 | 0.0 | chrX | 6814856 | 6814880 | - |
| 818 | miR-501-3p | MI0004703_1 | 0 | 0.0 | chrX | 6818396 | 6818417 | - |
| 819 | miR-501-5p | MI0004703_2 | 0 | 0.0 | chrX | 6818433 | 6818454 | - |
| 820 | miR-362-3p | MI0000763_1 | 0 | 0.0 | chrX | 6819110 | 6819131 | - |
| 821 | miR-362-5p | MI0000763_2 | 0 | 0.0 | chrX | 6819145 | 6819168 | - |
| 822 | miR-188-3p | MI0000230_1 | 0 | 0.0 | chrX | 6825118 | 6825138 | - |
| 823 | miR-188-5p | MI0000230_2 | 0 | 0.0 | chrX | 6825157 | 6825177 | - |
| 824 | miR-532-3p | MI0003206_1 | 0 | 0.0 | chrX | 6825545 | 6825566 | - |
| 825 | miR-532-5p | MI0003206_2 | 0 | 0.0 | chrX | 6825582 | 6825603 | - |
| 826 | miR-221-3p | MI0000709_1 | 0 | 0.0 | chrX | 18723433 | 18723455 | - |
| 827 | miR-221-5p | MI0000709_2 | 0 | 0.0 | chrX | 18723470 | 18723495 | - |
| 828 | miR-222-3p | MI0000710_1 | 0 | 0.0 | chrX | 18724029 | 18724049 | - |
| 829 | miR-222-5p | MI0000710_2 | 0 | 0.0 | chrX | 18724066 | 18724086 | - |
| 830 | miR-5617-3p | MI0019185_1 | 0 | 0.0 | chrX | 20440252 | 20440272 | - |
| 831 | miR-5617-5p | MI0019185_2 | 0 | 0.0 | chrX | 20440286 | 20440308 | - |
| 832 | miR-3110-3p | MI0014107_2 | 0 | 0.0 | chrX | 35563665 | 35563686 | + |
| 833 | miR-717 | MI0004704_1 | 0 | 0.0 | chrX | 49775638 | 49775659 | - |
| 834 | miR-363-3p | MI0000765_1 | 0 | 0.0 | chrX | 50094874 | 50094895 | - |
| 835 | miR-363-5p | MI0000765_2 | 0 | 0.0 | chrX | 50094917 | 50094938 | - |
| 836 | miR-92a-3p | MI0000580_1 | 0 | 0.0 | chrX | 50095031 | 50095051 | - |
| 837 | miR-92a-2-5p | MI0000580_2 | 0 | 0.0 | chrX | 50095069 | 50095090 | - |
| 838 | miR-19b-3p | MI0000546_1 | 0 | 0.0 | chrX | 50095168 | 50095190 | - |
| 839 | miR-19b-2-5p | MI0000546_2 | 0 | 0.0 | chrX | 50095210 | 50095233 | - |
| 840 | miR-20b-3p | MI0003536_1 | 0 | 0.0 | chrX | 50095299 | 50095320 | - |
| 841 | miR-20b-5p | MI0003536_2 | 0 | 0.0 | chrX | 50095336 | 50095358 | - |
| 842 | miR-18b-3p | MI0005483_1 | 0 | 0.0 | chrX | 50095519 | 50095540 | - |
| 843 | miR-18b-5p | MI0005483_2 | 0 | 0.0 | chrX | 50095558 | 50095580 | - |
| 844 | miR-106a-3p | MI0000406_1 | 0 | 0.0 | chrX | 50095682 | 50095704 | - |
| 845 | miR-106a-5p | MI0000406_2 | 0 | 0.0 | chrX | 50095718 | 50095740 | - |
| 846 | miR-450b-3p | MI0004705_1 | 0 | 0.0 | chrX | 50401185 | 50401206 | - |
| 847 | miR-450b-5p | MI0004705_2 | 0 | 0.0 | chrX | 50401221 | 50401242 | - |
| 848 | miR-450a-1-3p | MI0001653_1 | 0 | 0.0 | chrX | 50401348 | 50401369 | - |
| 849 | miR-450a-5p | MI0001653_2 | 0 | 0.0 | chrX | 50401383 | 50401404 | - |
| 850 | miR-450a-2-3p | MI0003537_1 | 0 | 0.0 | chrX | 50401481 | 50401502 | - |
| 851 | miR-450a-5p | MI0003537_2 | 0 | 0.0 | chrX | 50401516 | 50401537 | - |
| 852 | miR-542-3p | MI0003522_1 | 0 | 0.0 | chrX | 50402592 | 50402613 | - |
| 853 | miR-542-5p | MI0003522_2 | 0 | 0.0 | chrX | 50402630 | 50402651 | - |
| 854 | miR-351-3p | MI0000643_1 | 0 | 0.0 | chrX | 50406453 | 50406473 | - |
| 855 | miR-351-5p | MI0000643_2 | 0 | 0.0 | chrX | 50406492 | 50406515 | - |
| 856 | miR-503-3p | MI0003538_1 | 0 | 0.0 | chrX | 50407164 | 50407185 | - |
| 857 | miR-503-5p | MI0003538_2 | 0 | 0.0 | chrX | 50407204 | 50407226 | - |
| 858 | miR-322-3p | MI0000590_1 | 0 | 0.0 | chrX | 50407446 | 50407466 | - |
| 859 | miR-322-5p | MI0000590_2 | 0 | 0.0 | chrX | 50407483 | 50407504 | - |
| 860 | miR-5116 | MI0018025_1 | 0 | 0.0 | chrX | 53841069 | 53841089 | - |
| 861 | miR-504-3p | MI0005515_1 | 0 | 0.0 | chrX | 56350846 | 56350867 | - |
| 862 | miR-504-5p | MI0005515_2 | 0 | 0.0 | chrX | 56350882 | 56350903 | - |
| 863 | miR-505-3p | MI0004706_1 | 0 | 0.0 | chrX | 57647591 | 57647612 | - |
| 864 | miR-505-5p | MI0004706_2 | 0 | 0.0 | chrX | 57647626 | 57647648 | - |
| 865 | miR-743a-3p | MI0005207_1 | 0 | 0.0 | chrX | 64029935 | 64029956 | - |
| 866 | miR-743a-5p | MI0005207_2 | 0 | 0.0 | chrX | 64029969 | 64029990 | - |
| 867 | miR-743b-3p | MI0005470_1 | 0 | 0.0 | chrX | 64030441 | 64030462 | - |
| 868 | miR-743b-5p | MI0005470_2 | 0 | 0.0 | chrX | 64030477 | 64030497 | - |
| 869 | miR-742-3p | MI0005206_1 | 0 | 0.0 | chrX | 64033551 | 64033572 | - |
| 870 | miR-742-5p | MI0005206_2 | 0 | 0.0 | chrX | 64033587 | 64033607 | - |
| 871 | miR-883a-3p | MI0005476_1 | 0 | 0.0 | chrX | 64033943 | 64033964 | - |
| 872 | miR-883a-5p | MI0005476_2 | 0 | 0.0 | chrX | 64033978 | 64033999 | - |
| 873 | miR-883b-3p | MI0005477_1 | 0 | 0.0 | chrX | 64043075 | 64043096 | - |
| 874 | miR-883b-5p | MI0005477_2 | 0 | 0.0 | chrX | 64043111 | 64043132 | - |
| 875 | miR-471-3p | MI0002406_1 | 0 | 0.0 | chrX | 64045774 | 64045795 | - |
| 876 | miR-471-5p | MI0002406_2 | 0 | 0.0 | chrX | 64045809 | 64045830 | - |
| 877 | miR-741-3p | MI0005205_1 | 0 | 0.0 | chrX | 64049984 | 64050006 | - |
| 878 | miR-741-5p | MI0005205_2 | 0 | 0.0 | chrX | 64050022 | 64050044 | - |
| 879 | miR-463-3p | MI0002398_1 | 0 | 0.0 | chrX | 64052411 | 64052432 | - |
| 880 | miR-463-5p | MI0002398_2 | 0 | 0.0 | chrX | 64052448 | 64052469 | - |
| 881 | miR-880-3p | MI0005473_1 | 0 | 0.0 | chrX | 64053715 | 64053736 | - |
| 882 | miR-880-5p | MI0005473_2 | 0 | 0.0 | chrX | 64053752 | 64053772 | - |
| 883 | miR-878-3p | MI0005548_1 | 0 | 0.0 | chrX | 64054693 | 64054714 | - |
| 884 | miR-878-5p | MI0005548_2 | 0 | 0.0 | chrX | 64054729 | 64054750 | - |
| 885 | miR-881-3p | MI0005474_1 | 0 | 0.0 | chrX | 64055129 | 64055150 | - |
| 886 | miR-881-5p | MI0005474_2 | 0 | 0.0 | chrX | 64055161 | 64055182 | - |
| 887 | miR-871-3p | MI0005471_1 | 0 | 0.0 | chrX | 64063613 | 64063635 | - |
| 888 | miR-871-5p | MI0005471_2 | 0 | 0.0 | chrX | 64063647 | 64063669 | - |
| 889 | miR-470-3p | MI0002405_1 | 0 | 0.0 | chrX | 64067136 | 64067157 | - |
| 890 | miR-470-5p | MI0002405_2 | 0 | 0.0 | chrX | 64067170 | 64067192 | - |
| 891 | miR-465c-3p | MI0005500_1 | 0 | 0.0 | chrX | 64079140 | 64079161 | - |
| 892 | miR-465c-5p | MI0005500_2 | 0 | 0.0 | chrX | 64079179 | 64079200 | - |
| 893 | miR-465b-3p | MI0005498_1 | 0 | 0.0 | chrX | 64082387 | 64082408 | - |
| 894 | miR-465b-5p | MI0005498_2 | 0 | 0.0 | chrX | 64082424 | 64082445 | - |
| 895 | miR-465c-3p | MI0005501_1 | 0 | 0.0 | chrX | 64085702 | 64085723 | - |
| 896 | miR-465c-5p | MI0005501_2 | 0 | 0.0 | chrX | 64085741 | 64085762 | - |
| 897 | miR-465b-3p | MI0005499_1 | 0 | 0.0 | chrX | 64088949 | 64088970 | - |
| 898 | miR-465b-5p | MI0005499_2 | 0 | 0.0 | chrX | 64088986 | 64089007 | - |
| 899 | miR-465a-3p | MI0002400_1 | 0 | 0.0 | chrX | 64092238 | 64092259 | - |
| 900 | miR-465a-5p | MI0002400_2 | 0 | 0.0 | chrX | 64092274 | 64092296 | - |
| 901 | miR-201-3p | MI0000244_1 | 0 | 0.0 | chrX | 65241274 | 65241296 | - |
| 902 | miR-201-5p | MI0000244_2 | 0 | 0.0 | chrX | 65241310 | 65241331 | - |
| 903 | miR-547-3p | MI0003523_1 | 0 | 0.0 | chrX | 65241558 | 65241578 | - |
| 904 | miR-547-5p | MI0003523_2 | 0 | 0.0 | chrX | 65241595 | 65241615 | - |
| 905 | miR-509-3p | MI0005516_1 | 0 | 0.0 | chrX | 65263290 | 65263310 | - |
| 906 | miR-509-5p | MI0005516_2 | 0 | 0.0 | chrX | 65263324 | 65263345 | - |
| 907 | miR-224-3p | MI0000711_1 | 0 | 0.0 | chrX | 69506376 | 69506397 | - |
| 908 | miR-224-5p | MI0000711_2 | 0 | 0.0 | chrX | 69506424 | 69506444 | - |
| 909 | miR-452-3p | MI0001734_1 | 0 | 0.0 | chrX | 69507574 | 69507595 | - |
| 910 | miR-452-5p | MI0001734_2 | 0 | 0.0 | chrX | 69507610 | 69507631 | - |
| 911 | miR-767 | MI0012530_1 | 0 | 0.0 | chrX | 69835389 | 69835409 | - |
| 912 | miR-105 | MI0005481_1 | 0 | 0.0 | chrX | 69836886 | 69836908 | - |
| 913 | miR-2137 | MI0010750_1 | 0 | 0.0 | chrX | 70237452 | 70237472 | + |
| 914 | miR-5132 | MI0018044_1 | 0 | 0.0 | chrX | 71268910 | 71268930 | - |
| 915 | miR-718 | MI0004707_1 | 0 | 0.0 | chrX | 71269196 | 71269216 | - |
| 916 | miR-1906 | MI0015984_1 | 0 | 0.0 | chrX | 86004824 | 86004845 | - |
| 917 | miR-421-3p | MI0005496_1 | 0 | 0.0 | chrX | 100768269 | 100768291 | - |
| 918 | miR-421-5p | MI0005496_2 | 0 | 0.0 | chrX | 100768305 | 100768325 | - |
| 919 | miR-374-3p | MI0004125_1 | 0 | 0.0 | chrX | 100768414 | 100768435 | - |
| 920 | miR-374-5p | MI0004125_2 | 0 | 0.0 | chrX | 100768451 | 100768472 | - |
| 921 | miR-374c-3p | MI0014108_2 | 0 | 0.0 | chrX | 100768452 | 100768470 | + |
| 922 | miR-672-3p | MI0004258_1 | 0 | 0.0 | chrX | 101311531 | 101311551 | - |
| 923 | miR-672-5p | MI0004258_2 | 0 | 0.0 | chrX | 101311567 | 101311589 | - |
| 924 | miR-384-3p | MI0001146_1 | 0 | 0.0 | chrX | 102539631 | 102539652 | - |
| 925 | miR-384-5p | MI0001146_2 | 0 | 0.0 | chrX | 102539671 | 102539693 | - |
| 926 | miR-325-3p | MI0000597_1 | 0 | 0.0 | chrX | 102574444 | 102574465 | - |
| 927 | miR-325-5p | MI0000597_2 | 0 | 0.0 | chrX | 102574481 | 102574503 | - |
| 928 | miR-361-3p | MI0000761_1 | 0 | 0.0 | chrX | 110188436 | 110188460 | - |
| 929 | miR-361-5p | MI0000761_2 | 0 | 0.0 | chrX | 110188476 | 110188497 | - |
| 930 | miR-3112-5p | MI0014109_1 | 0 | 0.0 | chrX | 130609869 | 130609889 | + |
| 931 | miR-3112-3p | MI0014109_2 | 0 | 0.0 | chrX | 130609905 | 130609925 | + |
| 932 | miR-1970 | MI0009968_1 | 0 | 0.0 | chrX | 132320713 | 132320735 | - |
| 933 | miR-3475 | MI0004220_1 | 0 | 0.0 | chrX | 136845531 | 136845551 | + |
| 934 | miR-680 | MI0004641_1 | 0 | 0.0 | chrX | 140732333 | 140732353 | + |
| 935 | miR-3472 | MI0014704_1 | 0 | 0.0 | chrX | 141473933 | 141473957 | + |
| 936 | miR-764-5p | MI0004310_1 | 0 | 0.0 | chrX | 143436828 | 143436846 | + |
| 937 | miR-764-3p | MI0004310_2 | 0 | 0.0 | chrX | 143436867 | 143436888 | + |
| 938 | miR-1912-5p | MI0014110_1 | 0 | 0.0 | chrX | 143443999 | 143444020 | + |
| 939 | miR-1912-3p | MI0014110_2 | 0 | 0.0 | chrX | 143444036 | 143444057 | + |
| 940 | miR-1264-5p | MI0004130_1 | 0 | 0.0 | chrX | 143445157 | 143445178 | + |
| 941 | miR-1264-3p | MI0004130_2 | 0 | 0.0 | chrX | 143445198 | 143445219 | + |
| 942 | miR-1298-3p | MI0004300_2 | 0 | 0.0 | chrX | 143499513 | 143499534 | + |
| 943 | miR-448-5p | MI0001638_1 | 0 | 0.0 | chrX | 143592764 | 143592785 | + |
| 944 | miR-448-3p | MI0001638_2 | 0 | 0.0 | chrX | 143592824 | 143592845 | + |
| 945 | miR-3113-5p | MI0014111_1 | 0 | 0.0 | chrX | 148294118 | 148294139 | + |
| 946 | miR-3113-3p | MI0014111_2 | 0 | 0.0 | chrX | 148294149 | 148294168 | + |
| 947 | miR-3473 | MI0014706_1 | 0 | 0.0 | chrX | 159312852 | 159312869 | - |
| 948 | miR-879-3p | MI0005472_2 | 0 | 0.0 | chr5 | 9375748 | 9375769 | + |
| 949 | miR-5109 | MI0018018_1 | 0 | 0.0 | chr5 | 17282577 | 17282599 | - |
| 950 | miR-3096-3p | MI0014089_1 | 0 | 0.0 | chr5 | 23216809 | 23216834 | - |
| 951 | miR-3096-5p | MI0014089_2 | 0 | 0.0 | chr5 | 23216862 | 23216879 | - |
| 952 | miR-3096b-5p | MI0016969_1 | 0 | 0.0 | chr5 | 23358029 | 23358049 | + |
| 953 | miR-3096b-3p | MI0016969_2 | 0 | 0.0 | chr5 | 23358079 | 23358098 | + |
| 954 | miR-5625-5p | MI0019194_1 | 0 | 0.0 | chr5 | 30950309 | 30950329 | + |
| 955 | miR-5625-3p | MI0019194_2 | 0 | 0.0 | chr5 | 30950364 | 30950388 | + |
| 956 | miR-467g | MI0006301_1 | 0 | 0.0 | chr5 | 35075521 | 35075541 | - |
| 957 | miR-3097-5p | MI0014090_1 | 0 | 0.0 | chr5 | 35363699 | 35363721 | + |
| 958 | miR-3097-3p | MI0014090_2 | 0 | 0.0 | chr5 | 35363743 | 35363764 | + |
| 959 | miR-574-5p | MI0005518_1 | 0 | 0.0 | chr5 | 65361567 | 65361589 | + |
| 960 | miR-5098 | MI0018006_1 | 0 | 0.0 | chr5 | 77701792 | 77701812 | + |
| 961 | miR-1187 | MI0006285_1 | 0 | 0.0 | chr5 | 83227989 | 83228011 | - |
| 962 | miR-3969 | MI0016978_1 | 0 | 0.0 | chr5 | 88024988 | 88025007 | - |
| 963 | miR-1961 | MI0009958_1 | 0 | 0.0 | chr5 | 93217491 | 93217507 | - |
| 964 | miR-703 | MI0004687_1 | 0 | 0.0 | chr5 | 98904635 | 98904655 | - |
| 965 | miR-5619-5p | MI0019187_1 | 0 | 0.0 | chr5 | 104477022 | 104477042 | + |
| 966 | miR-5619-3p | MI0019187_2 | 0 | 0.0 | chr5 | 104477061 | 104477082 | + |
| 967 | miR-3965 | MI0016972_1 | 0 | 0.0 | chr5 | 134802364 | 134802382 | + |
| 968 | miR-721 | MI0004708_1 | 0 | 0.0 | chr5 | 136851647 | 136851667 | - |
| 969 | miR-702 | MI0004686_1 | 0 | 0.0 | chr5 | 137467390 | 137467410 | + |
| 970 | miR-25-3p | MI0000689_1 | 0 | 0.0 | chr5 | 138606560 | 138606581 | - |
| 971 | miR-25-5p | MI0000689_2 | 0 | 0.0 | chr5 | 138606598 | 138606619 | - |
| 972 | miR-93-3p | MI0000581_1 | 0 | 0.0 | chr5 | 138606764 | 138606785 | - |
| 973 | miR-93-5p | MI0000581_2 | 0 | 0.0 | chr5 | 138606802 | 138606824 | - |
| 974 | miR-106b-3p | MI0000407_1 | 0 | 0.0 | chr5 | 138606974 | 138606995 | - |
| 975 | miR-106b-5p | MI0000407_2 | 0 | 0.0 | chr5 | 138607015 | 138607035 | - |
| 976 | miR-339-3p | MI0000621_1 | 0 | 0.0 | chr5 | 139845627 | 139845649 | - |
| 977 | miR-339-5p | MI0000621_2 | 0 | 0.0 | chr5 | 139845662 | 139845684 | - |
| 978 | miR-5105 | MI0018013_1 | 0 | 0.0 | chr5 | 147072589 | 147072608 | + |
| 979 | miR-680 | MI0004642_1 | 0 | 0.0 | chr12 | 35879521 | 35879541 | - |
| 980 | miR-5099 | MI0018007_1 | 0 | 0.0 | chr12 | 37542837 | 37542856 | + |
| 981 | miR-1938 | MI0009927_1 | 0 | 0.0 | chr12 | 40949369 | 40949390 | - |
| 982 | miR-5627-5p | MI0019198_1 | 0 | 0.0 | chr12 | 45311300 | 45311321 | + |
| 983 | miR-5627-3p | MI0019198_2 | 0 | 0.0 | chr12 | 45311337 | 45311360 | + |
| 984 | miR-1892 | MI0008320_1 | 0 | 0.0 | chr12 | 55746930 | 55746951 | - |
| 985 | miR-681 | MI0004643_1 | 0 | 0.0 | chr12 | 70864910 | 70864930 | - |
| 986 | miR-5101 | MI0018009_1 | 0 | 0.0 | chr12 | 77010141 | 77010162 | + |
| 987 | miR-5135 | MI0018047_1 | 0 | 0.0 | chr12 | 77634130 | 77634153 | - |
| 988 | miR-1843-3p | MI0004155_1 | 0 | 0.0 | chr12 | 81492602 | 81492623 | - |
| 989 | miR-1843-5p | MI0004155_2 | 0 | 0.0 | chr12 | 81492641 | 81492661 | - |
| 990 | miR-3067-5p | MI0014029_1 | 0 | 0.0 | chr12 | 82267152 | 82267174 | + |
| 991 | miR-3067-3p | MI0014029_2 | 0 | 0.0 | chr12 | 82267188 | 82267210 | + |
| 992 | miR-3068-3p | MI0014030_1 | 0 | 0.0 | chr12 | 88778635 | 88778656 | - |
| 993 | miR-3068-5p | MI0014030_2 | 0 | 0.0 | chr12 | 88778674 | 88778697 | - |
| 994 | miR-1190 | MI0006294_1 | 0 | 0.0 | chr12 | 102259902 | 102259923 | - |
| 995 | miR-1936 | MI0009925_1 | 0 | 0.0 | chr12 | 103923153 | 103923175 | - |
| 996 | miR-3069-3p | MI0014031_1 | 0 | 0.0 | chr12 | 106269288 | 106269309 | - |
| 997 | miR-3069-5p | MI0014031_2 | 0 | 0.0 | chr12 | 106269327 | 106269350 | - |
| 998 | miR-1906 | MI0008321_1 | 0 | 0.0 | chr12 | 110782798 | 110782819 | + |
| 999 | miR-770-5p | MI0004203_1 | 0 | 0.0 | chr12 | 110801920 | 110801941 | + |
| 1000 | miR-770-3p | MI0004203_2 | 0 | 0.0 | chr12 | 110801954 | 110801975 | + |
| 1001 | miR-493-5p | MI0005514_1 | 0 | 0.0 | chr12 | 110818453 | 110818471 | + |
| 1002 | miR-493-3p | MI0005514_2 | 0 | 0.0 | chr12 | 110818493 | 110818515 | + |
| 1003 | miR-337-5p | MI0000615_1 | 0 | 0.0 | chr12 | 110824025 | 110824045 | + |
| 1004 | miR-3544-3p | MI0019179_1 | 0 | 0.0 | chr12 | 110824023 | 110824044 | - |
| 1005 | miR-3544-5p | MI0019179_2 | 0 | 0.0 | chr12 | 110824061 | 110824083 | - |
| 1006 | miR-540-3p | MI0003518_2 | 0 | 0.0 | chr12 | 110824332 | 110824351 | + |
| 1007 | miR-665-5p | MI0004171_1 | 0 | 0.0 | chr12 | 110824541 | 110824566 | + |
| 1008 | miR-665-3p | MI0004171_2 | 0 | 0.0 | chr12 | 110824578 | 110824597 | + |
| 1009 | miR-3070a-5p | MI0014032_1 | 0 | 0.0 | chr12 | 110826169 | 110826191 | + |
| 1010 | miR-3070a-3p | MI0014032_2 | 0 | 0.0 | chr12 | 110826209 | 110826230 | + |
| 1011 | miR-3070b-5p | MI0014033_1 | 0 | 0.0 | chr12 | 110826818 | 110826840 | + |
| 1012 | miR-3070b-3p | MI0014033_2 | 0 | 0.0 | chr12 | 110826858 | 110826879 | + |
| 1013 | miR-433-5p | MI0001525_1 | 0 | 0.0 | chr12 | 110829939 | 110829960 | + |
| 1014 | miR-432 | MI0012528_1 | 0 | 0.0 | chr12 | 110833216 | 110833238 | + |
| 1015 | miR-3071-3p | MI0014034_1 | 0 | 0.0 | chr12 | 110833539 | 110833560 | - |
| 1016 | miR-3071-5p | MI0014034_2 | 0 | 0.0 | chr12 | 110833573 | 110833594 | - |
| 1017 | miR-341-5p | MI0000625_1 | 0 | 0.0 | chr12 | 110849731 | 110849751 | + |
| 1018 | miR-1188-5p | MI0006290_1 | 0 | 0.0 | chr12 | 110850051 | 110850071 | + |
| 1019 | miR-1188-3p | MI0006290_2 | 0 | 0.0 | chr12 | 110850087 | 110850111 | + |
| 1020 | miR-370-5p | MI0001165_1 | 0 | 0.0 | chr12 | 110856480 | 110856499 | + |
| 1021 | miR-882 | MI0005475_1 | 0 | 0.0 | chr12 | 110920417 | 110920438 | + |
| 1022 | miR-299b-3p | MI0019180_1 | 0 | 0.0 | chr12 | 110948855 | 110948872 | - |
| 1023 | miR-299b-5p | MI0019180_2 | 0 | 0.0 | chr12 | 110948883 | 110948903 | - |
| 1024 | miR-380-3p | MI0000797_2 | 0 | 0.0 | chr12 | 110950052 | 110950073 | + |
| 1025 | miR-1197-5p | MI0006305_1 | 0 | 0.0 | chr12 | 110950571 | 110950591 | + |
| 1026 | miR-758-5p | MI0004129_1 | 0 | 0.0 | chr12 | 110951033 | 110951053 | + |
| 1027 | miR-758-3p | MI0004129_2 | 0 | 0.0 | chr12 | 110951068 | 110951086 | + |
| 1028 | miR-494-5p | MI0003532_1 | 0 | 0.0 | chr12 | 110953544 | 110953565 | + |
| 1029 | miR-679-5p | MI0004638_1 | 0 | 0.0 | chr12 | 110953797 | 110953818 | + |
| 1030 | miR-679-3p | MI0004638_2 | 0 | 0.0 | chr12 | 110953834 | 110953855 | + |
| 1031 | miR-1193-5p | MI0006298_1 | 0 | 0.0 | chr12 | 110953926 | 110953945 | + |
| 1032 | miR-666-3p | MI0004553_2 | 0 | 0.0 | chr12 | 110955350 | 110955371 | + |
| 1033 | miR-543-5p | MI0003519_1 | 0 | 0.0 | chr12 | 110955479 | 110955499 | + |
| 1034 | miR-495-5p | MI0004639_1 | 0 | 0.0 | chr12 | 110956967 | 110956989 | + |
| 1035 | miR-667-5p | MI0004196_1 | 0 | 0.0 | chr12 | 110958230 | 110958254 | + |
| 1036 | miR-376c-5p | MI0003533_1 | 0 | 0.0 | chr12 | 110960943 | 110960964 | + |
| 1037 | miR-654-5p | MI0005520_1 | 0 | 0.0 | chr12 | 110961445 | 110961466 | + |
| 1038 | miR-654-3p | MI0005520_2 | 0 | 0.0 | chr12 | 110961480 | 110961501 | + |
| 1039 | miR-376a-5p | MI0000793_1 | 0 | 0.0 | chr12 | 110961996 | 110962017 | + |
| 1040 | miR-300-5p | MI0000400_1 | 0 | 0.0 | chr12 | 110962528 | 110962549 | + |
| 1041 | miR-487b-5p | MI0003534_1 | 0 | 0.0 | chr12 | 110965557 | 110965577 | + |
| 1042 | miR-539-3p | MI0003520_2 | 0 | 0.0 | chr12 | 110966386 | 110966407 | + |
| 1043 | miR-544-5p | MI0005555_1 | 0 | 0.0 | chr12 | 110967545 | 110967565 | + |
| 1044 | miR-544-3p | MI0005555_2 | 0 | 0.0 | chr12 | 110967581 | 110967602 | + |
| 1045 | miR-382-3p | MI0000799_2 | 0 | 0.0 | chr12 | 110972029 | 110972050 | + |
| 1046 | miR-668-5p | MI0004134_1 | 0 | 0.0 | chr12 | 110972943 | 110972966 | + |
| 1047 | miR-453 | MI0005497_1 | 0 | 0.0 | chr12 | 110973879 | 110973902 | + |
| 1048 | miR-496-5p | MI0004589_1 | 0 | 0.0 | chr12 | 110977342 | 110977362 | + |
| 1049 | miR-541-5p | MI0003521_1 | 0 | 0.0 | chr12 | 110980632 | 110980656 | + |
| 1050 | miR-541-3p | MI0003521_2 | 0 | 0.0 | chr12 | 110980676 | 110980697 | + |
| 1051 | miR-412-5p | MI0001164_1 | 0 | 0.0 | chr12 | 110981513 | 110981535 | + |
| 1052 | miR-412-3p | MI0001164_2 | 0 | 0.0 | chr12 | 110981550 | 110981569 | + |
| 1053 | miR-410-5p | MI0001161_1 | 0 | 0.0 | chr12 | 110981939 | 110981959 | + |
| 1054 | miR-3072-5p | MI0014035_1 | 0 | 0.0 | chr12 | 110986103 | 110986124 | + |
| 1055 | miR-3072-3p | MI0014035_2 | 0 | 0.0 | chr12 | 110986138 | 110986159 | + |
| 1056 | miR-1247-3p | MI0004120_1 | 0 | 0.0 | chr12 | 111516268 | 111516288 | - |
| 1057 | miR-1247-5p | MI0004120_2 | 0 | 0.0 | chr12 | 111516304 | 111516325 | - |
| 1058 | miR-3073-5p | MI0014036_1 | 0 | 0.0 | chr12 | 113347463 | 113347484 | + |
| 1059 | miR-3073-3p | MI0014036_2 | 0 | 0.0 | chr12 | 113347501 | 113347522 | + |
| 1060 | miR-3073b-3p | MI0019192_1 | 0 | 0.0 | chr12 | 113347461 | 113347482 | - |
| 1061 | miR-3073b-5p | MI0019192_2 | 0 | 0.0 | chr12 | 113347499 | 113347520 | - |
| 1062 | miR-153-5p | MI0000175_1 | 0 | 0.0 | chr12 | 118489300 | 118489316 | + |
| 1063 | miR-3471 | MI0014701_1 | 0 | 0.0 | chr4 | 3464250 | 3464271 | + |
| 1064 | miR-3471 | MI0014699_1 | 0 | 0.0 | chr4 | 10772732 | 10772753 | - |
| 1065 | miR-684 | MI0004648_1 | 0 | 0.0 | chr4 | 11061303 | 11061321 | + |
| 1066 | miR-876-3p | MI0005480_1 | 0 | 0.0 | chr4 | 36592416 | 36592437 | - |
| 1067 | miR-876-5p | MI0005480_2 | 0 | 0.0 | chr4 | 36592455 | 36592476 | - |
| 1068 | miR-873-3p | MI0005550_1 | 0 | 0.0 | chr4 | 36615553 | 36615573 | - |
| 1069 | miR-873-5p | MI0005550_2 | 0 | 0.0 | chr4 | 36615589 | 36615609 | - |
| 1070 | miR-207 | MI0000250_1 | 0 | 0.0 | chr4 | 40670001 | 40670023 | + |
| 1071 | miR-5123 | MI0018034_1 | 0 | 0.0 | chr4 | 40797090 | 40797113 | - |
| 1072 | miR-3094-5p | MI0014087_1 | 0 | 0.0 | chr4 | 40940729 | 40940749 | + |
| 1073 | miR-3094-3p | MI0014087_2 | 0 | 0.0 | chr4 | 40940771 | 40940791 | + |
| 1074 | miR-5106 | MI0018014_1 | 0 | 0.0 | chr4 | 44234069 | 44234091 | - |
| 1075 | miR-5120 | MI0018029_1 | 0 | 0.0 | chr4 | 44620408 | 44620430 | - |
| 1076 | miR-1958 | MI0009955_1 | 0 | 0.0 | chr4 | 48226006 | 48226027 | - |
| 1077 | miR-32-3p | MI0000691_1 | 0 | 0.0 | chr4 | 56908104 | 56908124 | - |
| 1078 | miR-32-5p | MI0000691_2 | 0 | 0.0 | chr4 | 56908144 | 56908165 | - |
| 1079 | miR-3095-3p | MI0014088_1 | 0 | 0.0 | chr4 | 58453895 | 58453917 | - |
| 1080 | miR-3095-5p | MI0014088_2 | 0 | 0.0 | chr4 | 58453936 | 58453957 | - |
| 1081 | miR-31-3p | MI0000579_1 | 0 | 0.0 | chr4 | 88556481 | 88556503 | - |
| 1082 | miR-31-5p | MI0000579_2 | 0 | 0.0 | chr4 | 88556518 | 88556539 | - |
| 1083 | miR-101a-3p | MI0000148_1 | 0 | 0.0 | chr4 | 101019562 | 101019582 | - |
| 1084 | miR-101a-5p | MI0000148_2 | 0 | 0.0 | chr4 | 101019598 | 101019619 | - |
| 1085 | miR-761 | MI0004306_1 | 0 | 0.0 | chr4 | 108690274 | 108690295 | + |
| 1086 | miR-1957 | MI0009954_1 | 0 | 0.0 | chr4 | 118802397 | 118802415 | + |
| 1087 | miR-30c-1-3p | MI0000547_1 | 0 | 0.0 | chr4 | 120442151 | 120442172 | - |
| 1088 | miR-30c-5p | MI0000547_2 | 0 | 0.0 | chr4 | 120442189 | 120442211 | - |
| 1089 | miR-30e-3p | MI0000259_1 | 0 | 0.0 | chr4 | 120445223 | 120445244 | - |
| 1090 | miR-30e-5p | MI0000259_2 | 0 | 0.0 | chr4 | 120445265 | 120445286 | - |
| 1091 | miR-697 | MI0004681_1 | 0 | 0.0 | chr4 | 124409012 | 124409032 | + |
| 1092 | miR-698 | MI0004682_1 | 0 | 0.0 | chr4 | 124421096 | 124421114 | + |
| 1093 | miR-692 | MI0004661_chr4_1 | 0 | 0.0 | chr4 | 125182049 | 125182069 | + |
| 1094 | miR-5122 | MI0018031_1 | 0 | 0.0 | chr4 | 132925699 | 132925718 | + |
| 1095 | miR-700-3p | MI0004684_1 | 0 | 0.0 | chr4 | 134972476 | 134972496 | - |
| 1096 | miR-700-5p | MI0004684_2 | 0 | 0.0 | chr4 | 134972516 | 134972537 | - |
| 1097 | miR-2139 | MI0010752_1 | 0 | 0.0 | chr4 | 139523446 | 139523469 | - |
| 1098 | miR-5616-5p | MI0019184_1 | 0 | 0.0 | chr4 | 148911972 | 148911992 | + |
| 1099 | miR-5616-3p | MI0019184_2 | 0 | 0.0 | chr4 | 148912010 | 148912031 | + |
| 1100 | miR-429-3p | MI0001642_1 | 0 | 0.0 | chr4 | 155428025 | 155428046 | - |
| 1101 | miR-429-5p | MI0001642_2 | 0 | 0.0 | chr4 | 155428063 | 155428083 | - |
| 1102 | miR-200a-3p | MI0000554_1 | 0 | 0.0 | chr4 | 155429020 | 155429041 | - |
| 1103 | miR-200a-5p | MI0000554_2 | 0 | 0.0 | chr4 | 155429058 | 155429079 | - |
| 1104 | miR-200b-3p | MI0000243_1 | 0 | 0.0 | chr4 | 155429794 | 155429815 | - |
| 1105 | miR-200b-5p | MI0000243_2 | 0 | 0.0 | chr4 | 155429830 | 155429851 | - |
| 1106 | miR-3060-5p | MI0014022_1 | 0 | 0.0 | chr11 | 4039380 | 4039399 | + |
| 1107 | miR-1933-3p | MI0009922_1 | 0 | 0.0 | chr11 | 21244603 | 21244624 | - |
| 1108 | miR-1933-5p | MI0009922_2 | 0 | 0.0 | chr11 | 21244643 | 21244666 | - |
| 1109 | miR-216b-3p | MI0004126_2 | 0 | 0.0 | chr11 | 28646244 | 28646265 | + |
| 1110 | miR-216a-3p | MI0000699_2 | 0 | 0.0 | chr11 | 28657058 | 28657079 | + |
| 1111 | miR-217-5p | MI0000731_1 | 0 | 0.0 | chr11 | 28663761 | 28663783 | + |
| 1112 | miR-217-3p | MI0000731_2 | 0 | 0.0 | chr11 | 28663798 | 28663820 | + |
| 1113 | miR-146a-3p | MI0000170_1 | 0 | 0.0 | chr11 | 43187901 | 43187922 | - |
| 1114 | miR-146a-5p | MI0000170_2 | 0 | 0.0 | chr11 | 43187937 | 43187958 | - |
| 1115 | miR-804 | MI0005203_1 | 0 | 0.0 | chr11 | 50171301 | 50171322 | - |
| 1116 | miR-744-3p | MI0004124_1 | 0 | 0.0 | chr11 | 65548244 | 65548265 | - |
| 1117 | miR-744-5p | MI0004124_2 | 0 | 0.0 | chr11 | 65548301 | 65548322 | - |
| 1118 | miR-3062-3p | MI0014024_1 | 0 | 0.0 | chr11 | 68804099 | 68804117 | - |
| 1119 | miR-3062-5p | MI0014024_2 | 0 | 0.0 | chr11 | 68804140 | 68804161 | - |
| 1120 | miR-467f | MI0006293_1 | 0 | 0.0 | chr11 | 69448921 | 69448941 | - |
| 1121 | miR-1934-3p | MI0009923_2 | 0 | 0.0 | chr11 | 69476594 | 69476615 | + |
| 1122 | miR-3971 | MI0016981_1 | 0 | 0.0 | chr11 | 75364982 | 75365003 | + |
| 1123 | miR-423-3p | MI0004637_1 | 0 | 0.0 | chr11 | 76891588 | 76891610 | - |
| 1124 | miR-423-5p | MI0004637_2 | 0 | 0.0 | chr11 | 76891624 | 76891646 | - |
| 1125 | miR-1935 | MI0009924_1 | 0 | 0.0 | chr11 | 85336230 | 85336251 | + |
| 1126 | miR-5110 | MI0018019_1 | 0 | 0.0 | chr11 | 85574177 | 85574201 | - |
| 1127 | miR-21-3p | MI0000569_1 | 0 | 0.0 | chr11 | 86397584 | 86397605 | - |
| 1128 | miR-21-5p | MI0000569_2 | 0 | 0.0 | chr11 | 86397622 | 86397643 | - |
| 1129 | miR-3063-3p | MI0014025_1 | 0 | 0.0 | chr11 | 95824618 | 95824639 | - |
| 1130 | miR-3063-5p | MI0014025_2 | 0 | 0.0 | chr11 | 95824661 | 95824681 | - |
| 1131 | miR-5119 | MI0018028_1 | 0 | 0.0 | chr11 | 98123952 | 98123970 | + |
| 1132 | miR-3064-3p | MI0014026_1 | 0 | 0.0 | chr11 | 106644009 | 106644030 | - |
| 1133 | miR-3064-5p | MI0014026_2 | 0 | 0.0 | chr11 | 106644050 | 106644071 | - |
| 1134 | miR-3968 | MI0016977_1 | 0 | 0.0 | chr11 | 115309283 | 115309303 | - |
| 1135 | miR-5621-5p | MI0019189_1 | 0 | 0.0 | chr11 | 115657138 | 115657160 | + |
| 1136 | miR-5621-3p | MI0019189_2 | 0 | 0.0 | chr11 | 115657180 | 115657200 | + |
| 1137 | miR-1932 | MI0009921_1 | 0 | 0.0 | chr11 | 119251838 | 119251859 | + |
| 1138 | miR-338-3p | MI0000619_1 | 0 | 0.0 | chr11 | 119876095 | 119876116 | - |
| 1139 | miR-338-5p | MI0000619_2 | 0 | 0.0 | chr11 | 119876130 | 119876151 | - |
| 1140 | miR-290-5p | MI0000388_1 | 0 | 0.0 | chr7 | 3218640 | 3218661 | + |
| 1141 | miR-290-3p | MI0000388_2 | 0 | 0.0 | chr7 | 3218675 | 3218698 | + |
| 1142 | miR-291a-3p | MI0000389_2 | 0 | 0.0 | chr7 | 3218969 | 3218990 | + |
| 1143 | miR-292-5p | MI0000390_1 | 0 | 0.0 | chr7 | 3219201 | 3219222 | + |
| 1144 | miR-292-3p | MI0000390_2 | 0 | 0.0 | chr7 | 3219239 | 3219262 | + |
| 1145 | miR-291b-5p | MI0003539_1 | 0 | 0.0 | chr7 | 3219495 | 3219516 | + |
| 1146 | miR-291b-3p | MI0003539_2 | 0 | 0.0 | chr7 | 3219530 | 3219551 | + |
| 1147 | miR-293-5p | MI0000391_1 | 0 | 0.0 | chr7 | 3220357 | 3220378 | + |
| 1148 | miR-293-3p | MI0000391_2 | 0 | 0.0 | chr7 | 3220391 | 3220412 | + |
| 1149 | miR-294-5p | MI0000392_1 | 0 | 0.0 | chr7 | 3220656 | 3220677 | + |
| 1150 | miR-294-3p | MI0000392_2 | 0 | 0.0 | chr7 | 3220692 | 3220713 | + |
| 1151 | miR-295-5p | MI0000393_1 | 0 | 0.0 | chr7 | 3220780 | 3220801 | + |
| 1152 | miR-3572 | MI0018037_1 | 0 | 0.0 | chr7 | 3607612 | 3607634 | + |
| 1153 | miR-3099-5p | MI0004485_1 | 0 | 0.0 | chr7 | 6756311 | 6756331 | + |
| 1154 | miR-3099-3p | MI0004485_2 | 0 | 0.0 | chr7 | 6756349 | 6756370 | + |
| 1155 | miR-5620-5p | MI0019188_1 | 0 | 0.0 | chr7 | 7251602 | 7251624 | + |
| 1156 | miR-5620-3p | MI0019188_2 | 0 | 0.0 | chr7 | 7251637 | 7251657 | + |
| 1157 | miR-297a-5p | MI0000395_1 | 0 | 0.0 | chr7 | 11543826 | 11543847 | - |
| 1158 | miR-3100-5p | MI0014092_1 | 0 | 0.0 | chr7 | 19672177 | 19672199 | + |
| 1159 | miR-3100-3p | MI0014092_2 | 0 | 0.0 | chr7 | 19672221 | 19672241 | + |
| 1160 | miR-343 | MI0005494_1 | 0 | 0.0 | chr7 | 19972037 | 19972056 | + |
| 1161 | miR-3101-3p | MI0014093_1 | 0 | 0.0 | chr7 | 27961039 | 27961059 | - |
| 1162 | miR-3101-5p | MI0014093_2 | 0 | 0.0 | chr7 | 27961077 | 27961097 | - |
| 1163 | miR-1963 | MI0009960_1 | 0 | 0.0 | chr7 | 29868690 | 29868712 | - |
| 1164 | miR-707 | MI0004691_1 | 0 | 0.0 | chr7 | 52105075 | 52105095 | + |
| 1165 | miR-5121 | MI0018030_1 | 0 | 0.0 | chr7 | 52382295 | 52382315 | - |
| 1166 | miR-344d-3p | MI0004524_1 | 0 | 0.0 | chr7 | 68828013 | 68828034 | - |
| 1167 | miR-344d-1-5p | MI0004524_2 | 0 | 0.0 | chr7 | 68828052 | 68828075 | - |
| 1168 | miR-344d-3p | MI0004619_1 | 0 | 0.0 | chr7 | 68830165 | 68830186 | - |
| 1169 | miR-344d-2-5p | MI0004619_2 | 0 | 0.0 | chr7 | 68830204 | 68830227 | - |
| 1170 | miR-344d-3p | MI0004227_1 | 0 | 0.0 | chr7 | 68871142 | 68871163 | - |
| 1171 | miR-344d-3-5p | MI0004227_2 | 0 | 0.0 | chr7 | 68871182 | 68871204 | - |
| 1172 | miR-344e-3p | MI0014094_1 | 0 | 0.0 | chr7 | 68880425 | 68880446 | - |
| 1173 | miR-344e-5p | MI0014094_2 | 0 | 0.0 | chr7 | 68880465 | 68880484 | - |
| 1174 | miR-344h-3p | MI0019196_1 | 0 | 0.0 | chr7 | 68884236 | 68884257 | - |
| 1175 | miR-344h-5p | MI0019196_2 | 0 | 0.0 | chr7 | 68884276 | 68884295 | - |
| 1176 | miR-344h-3p | MI0019197_1 | 0 | 0.0 | chr7 | 68887252 | 68887273 | - |
| 1177 | miR-344h-5p | MI0019197_2 | 0 | 0.0 | chr7 | 68887292 | 68887311 | - |
| 1178 | miR-344b-3p | MI0014095_1 | 0 | 0.0 | chr7 | 68935408 | 68935429 | - |
| 1179 | miR-344b-5p | MI0014095_2 | 0 | 0.0 | chr7 | 68935442 | 68935464 | - |
| 1180 | miR-344c-3p | MI0014096_1 | 0 | 0.0 | chr7 | 68982209 | 68982231 | - |
| 1181 | miR-344c-5p | MI0014096_2 | 0 | 0.0 | chr7 | 68982249 | 68982271 | - |
| 1182 | miR-344-3p | MI0000630_1 | 0 | 0.0 | chr7 | 69022668 | 69022690 | - |
| 1183 | miR-344-5p | MI0000630_2 | 0 | 0.0 | chr7 | 69022705 | 69022730 | - |
| 1184 | miR-344-3p | MI0005495_1 | 0 | 0.0 | chr7 | 69084922 | 69084944 | - |
| 1185 | miR-344-5p | MI0005495_2 | 0 | 0.0 | chr7 | 69084959 | 69084984 | - |
| 1186 | miR-344g-3p | MI0014097_1 | 0 | 0.0 | chr7 | 69127181 | 69127202 | - |
| 1187 | miR-344g-5p | MI0014097_2 | 0 | 0.0 | chr7 | 69127221 | 69127241 | - |
| 1188 | miR-344f-3p | MI0014098_1 | 0 | 0.0 | chr7 | 69191069 | 69191089 | - |
| 1189 | miR-344f-5p | MI0014098_2 | 0 | 0.0 | chr7 | 69191111 | 69191132 | - |
| 1190 | miR-344i | MI0019317_1 | 0 | 0.0 | chr7 | 69230167 | 69230186 | - |
| 1191 | miR-211-3p | MI0000708_2 | 0 | 0.0 | chr7 | 71350754 | 71350774 | + |
| 1192 | miR-7a-2-3p | MI0000729_2 | 0 | 0.0 | chr7 | 86033221 | 86033242 | + |
| 1193 | miR-1965 | MI0009962_1 | 0 | 0.0 | chr7 | 87297782 | 87297802 | - |
| 1194 | miR-326-5p | MI0000598_1 | 0 | 0.0 | chr7 | 106700798 | 106700821 | + |
| 1195 | miR-3102-3p | MI0014099_1 | 0 | 0.0 | chr7 | 108030821 | 108030843 | - |
| 1196 | miR-3102-3p.2-3p | MI0014099_2 | 0 | 0.0 | chr7 | 108030844 | 108030864 | - |
| 1197 | miR-3102-5p.2-5p | MI0014099_3 | 0 | 0.0 | chr7 | 108030881 | 108030901 | - |
| 1198 | miR-3102-5p | MI0014099_4 | 0 | 0.0 | chr7 | 108030902 | 108030923 | - |
| 1199 | miR-762 | MI0004215_1 | 0 | 0.0 | chr7 | 134852048 | 134852069 | + |
| 1200 | miR-3103-3p | MI0014100_1 | 0 | 0.0 | chr7 | 135431884 | 135431905 | - |
| 1201 | miR-3103-5p | MI0014100_2 | 0 | 0.0 | chr7 | 135431928 | 135431948 | - |
| 1202 | miR-5102 | MI0018010_1 | 0 | 0.0 | chr7 | 137977967 | 137977990 | + |
| 1203 | miR-1962 | MI0009959_1 | 0 | 0.0 | chr7 | 142757917 | 142757938 | + |
| 1204 | miR-202-3p | MI0000245_1 | 0 | 0.0 | chr7 | 147143594 | 147143615 | - |
| 1205 | miR-202-5p | MI0000245_2 | 0 | 0.0 | chr7 | 147143631 | 147143651 | - |
| 1206 | miR-210-3p | MI0000695_1 | 0 | 0.0 | chr7 | 148407306 | 148407327 | - |
| 1207 | miR-210-5p | MI0000695_2 | 0 | 0.0 | chr7 | 148407344 | 148407365 | - |
| 1208 | miR-3104-5p | MI0014101_1 | 0 | 0.0 | chr7 | 149178085 | 149178109 | + |
| 1209 | miR-3104-3p | MI0014101_2 | 0 | 0.0 | chr7 | 149178123 | 149178145 | + |
| 1210 | miR-675-3p | MI0004123_1 | 0 | 0.0 | chr7 | 149762983 | 149763004 | - |
| 1211 | miR-675-5p | MI0004123_2 | 0 | 0.0 | chr7 | 149763017 | 149763038 | - |
| 1212 | miR-483-3p | MI0003484_1 | 0 | 0.0 | chr7 | 149840837 | 149840857 | - |
| 1213 | miR-483-5p | MI0003484_2 | 0 | 0.0 | chr7 | 149840873 | 149840894 | - |
| 1214 | miR-3105-5p | MI0014102_1 | 0 | 0.0 | chr7 | 151195172 | 151195193 | + |
| 1215 | miR-5104 | MI0018012_1 | 0 | 0.0 | chr10 | 7556239 | 7556262 | + |
| 1216 | miR-3473b | MI0016997_1 | 0 | 0.0 | chr10 | 41390580 | 41390599 | + |
| 1217 | miR-1929-5p | MI0009918_1 | 0 | 0.0 | chr10 | 44079498 | 44079520 | + |
| 1218 | miR-1929-3p | MI0009918_2 | 0 | 0.0 | chr10 | 44079540 | 44079561 | + |
| 1219 | miR-466j | MI0006295_1 | 0 | 0.0 | chr10 | 60423550 | 60423572 | + |
| 1220 | miR-5108 | MI0018017_1 | 0 | 0.0 | chr10 | 61237543 | 61237561 | + |
| 1221 | miR-678 | MI0004635_1 | 0 | 0.0 | chr10 | 75670078 | 75670099 | - |
| 1222 | miR-1930-5p | MI0009919_1 | 0 | 0.0 | chr10 | 77103981 | 77104002 | + |
| 1223 | miR-1930-3p | MI0009919_2 | 0 | 0.0 | chr10 | 77104019 | 77104040 | + |
| 1224 | miR-1982-5p | MI0009993_1 | 0 | 0.0 | chr10 | 80291553 | 80291572 | + |
| 1225 | miR-1982.1-3p | MI0009993_2 | 0 | 0.0 | chr10 | 80291593 | 80291615 | + |
| 1226 | miR-5615-3p | MI0019181_1 | 0 | 0.0 | chr10 | 80567359 | 80567380 | - |
| 1227 | miR-5615-5p | MI0019181_2 | 0 | 0.0 | chr10 | 80567397 | 80567418 | - |
| 1228 | miR-5615-3p | MI0019182_2 | 0 | 0.0 | chr10 | 80567399 | 80567420 | + |
| 1229 | miR-3057-3p | MI0014020_2 | 0 | 0.0 | chr10 | 80734399 | 80734420 | + |
| 1230 | miR-135a-2-3p | MI0000715_1 | 0 | 0.0 | chr10 | 91534849 | 91534869 | - |
| 1231 | miR-135a-5p | MI0000715_2 | 0 | 0.0 | chr10 | 91534886 | 91534908 | - |
| 1232 | miR-1251-3p | MI0014021_1 | 0 | 0.0 | chr10 | 91599897 | 91599917 | - |
| 1233 | miR-1251-5p | MI0014021_2 | 0 | 0.0 | chr10 | 91599935 | 91599955 | - |
| 1234 | miR-1931 | MI0009920_1 | 0 | 0.0 | chr10 | 92625624 | 92625645 | + |
| 1235 | miR-331-3p | MI0000609_1 | 0 | 0.0 | chr10 | 93426528 | 93426548 | - |
| 1236 | miR-331-5p | MI0000609_2 | 0 | 0.0 | chr10 | 93426562 | 93426583 | - |
| 1237 | miR-3058-3p | MI0004418_1 | 0 | 0.0 | chr10 | 95021878 | 95021898 | - |
| 1238 | miR-3058-5p | MI0004418_2 | 0 | 0.0 | chr10 | 95021920 | 95021942 | - |
| 1239 | miR-3966 | MI0016975_1 | 0 | 0.0 | chr10 | 96886127 | 96886147 | + |
| 1240 | miR-3059-3p | MI0004412_2 | 0 | 0.0 | chr10 | 101235374 | 101235395 | + |
| 1241 | miR-763 | MI0004516_1 | 0 | 0.0 | chr10 | 119885108 | 119885129 | - |
| 1242 | let-7i-3p | MI0000138_1 | 0 | 0.0 | chr10 | 122422698 | 122422719 | - |
| 1243 | let-7i-5p | MI0000138_2 | 0 | 0.0 | chr10 | 122422754 | 122422775 | - |
| 1244 | miR-546 | MI0003517_1 | 0 | 0.0 | chr10 | 126435527 | 126435542 | + |
| 1245 | miR-677-3p | MI0004634_2 | 0 | 0.0 | chr10 | 127522391 | 127522416 | + |
| 1246 | miR-653-3p | MI0005557_1 | 0 | 0.0 | chr6 | 3671315 | 3671335 | - |
| 1247 | miR-653-5p | MI0005557_2 | 0 | 0.0 | chr6 | 3671355 | 3671375 | - |
| 1248 | miR-489-3p | MI0003476_1 | 0 | 0.0 | chr6 | 3671922 | 3671944 | - |
| 1249 | miR-489-5p | MI0003476_2 | 0 | 0.0 | chr6 | 3671957 | 3671981 | - |
| 1250 | miR-592-3p | MI0004127_1 | 0 | 0.0 | chr6 | 27886672 | 27886693 | - |
| 1251 | miR-592-5p | MI0004127_2 | 0 | 0.0 | chr6 | 27886711 | 27886733 | - |
| 1252 | miR-182-3p | MI0000224_1 | 0 | 0.0 | chr6 | 30115923 | 30115943 | - |
| 1253 | miR-182-5p | MI0000224_2 | 0 | 0.0 | chr6 | 30115962 | 30115986 | - |
| 1254 | miR-96-3p | MI0000583_1 | 0 | 0.0 | chr6 | 30119465 | 30119486 | - |
| 1255 | miR-96-5p | MI0000583_2 | 0 | 0.0 | chr6 | 30119506 | 30119528 | - |
| 1256 | miR-183-3p | MI0000225_1 | 0 | 0.0 | chr6 | 30119672 | 30119693 | - |
| 1257 | miR-183-5p | MI0000225_2 | 0 | 0.0 | chr6 | 30119711 | 30119732 | - |
| 1258 | miR-29a-3p | MI0000576_1 | 0 | 0.0 | chr6 | 31012673 | 31012694 | - |
| 1259 | miR-29a-5p | MI0000576_2 | 0 | 0.0 | chr6 | 31012711 | 31012732 | - |
| 1260 | miR-29b-3p | MI0000143_1 | 0 | 0.0 | chr6 | 31013025 | 31013047 | - |
| 1261 | miR-29b-1-5p | MI0000143_2 | 0 | 0.0 | chr6 | 31013067 | 31013088 | - |
| 1262 | miR-704 | MI0004688_1 | 0 | 0.0 | chr6 | 47753589 | 47753609 | - |
| 1263 | miR-148a-3p | MI0000550_1 | 0 | 0.0 | chr6 | 51219828 | 51219849 | - |
| 1264 | miR-148a-5p | MI0000550_2 | 0 | 0.0 | chr6 | 51219866 | 51219887 | - |
| 1265 | miR-196b-3p | MI0001151_1 | 0 | 0.0 | chr6 | 52180093 | 52180114 | - |
| 1266 | miR-196b-5p | MI0001151_2 | 0 | 0.0 | chr6 | 52180128 | 52180149 | - |
| 1267 | miR-468-3p | MI0002403_1 | 0 | 0.0 | chr6 | 81846596 | 81846618 | - |
| 1268 | miR-468-5p | MI0002403_2 | 0 | 0.0 | chr6 | 81846623 | 81846649 | - |
| 1269 | miR-3470a | MI0014696_1 | 0 | 0.0 | chr6 | 83040354 | 83040374 | - |
| 1270 | miR-705 | MI0004689_1 | 0 | 0.0 | chr6 | 85286335 | 85286354 | - |
| 1271 | miR-706 | MI0004690_1 | 0 | 0.0 | chr6 | 119984308 | 119984329 | - |
| 1272 | miR-141-3p | MI0000166_1 | 0 | 0.0 | chr6 | 124667935 | 124667956 | - |
| 1273 | miR-141-5p | MI0000166_2 | 0 | 0.0 | chr6 | 124667977 | 124667998 | - |
| 1274 | miR-200c-3p | MI0000694_1 | 0 | 0.0 | chr6 | 124668342 | 124668364 | - |
| 1275 | miR-200c-5p | MI0000694_2 | 0 | 0.0 | chr6 | 124668383 | 124668404 | - |
| 1276 | miR-3098-3p | MI0014091_2 | 0 | 0.0 | chr6 | 125145863 | 125145883 | + |
| 1277 | miR-680 | MI0004640_1 | 0 | 0.0 | chr6 | 129641559 | 129641579 | + |
| Small RNA sequencing. Total RNA was collected from 4 mice and pooled as 1 sample. miRBase was used as the source of miRNA names and IDs. | | | | | | | | |
