# Supplementary material for: Decreased miR-26a Expression Correlates with the Progression of Podocyte Injury in Autoimmune Glomerulonephritis
Source: PLoS One. 2014 Oct 17;9(10):e110383. doi: 10.1371/journal.pone.0110383 (PMC4201534; doi:10.1371/journal.pone.0110383)
Supplement: Table S6 — Correlation between indices for podocyte injuries and urinary albumin/creatinine ratio in GN-model mice. (DOCX) [file pone.0110383.s006.docx]

**Table S6. Correlation between indices for podocyte injuries and urinary albumin/creatinine ratio in GN-model mice.**

| **Indices for podocyte injuries and GN** | |  | **Urinary ACR** |
| --- | --- | --- | --- |
| Glomerular mRNA expression of podocyte proteins | *Actn4* |  | -0.410 |
|  | *Cd2ap* |  | -0.446 |
|  | *Myh9* |  | -0.243 |
|  | *Nphs1* |  | -0.222 |
|  | *Nphs2* |  | -0.287 |
|  | *Podxl* |  | -0.676** |
|  | *Synpo* |  | -0.768** |
|  | *Vim* |  | -0.611** |
|  | *Wt1* |  | -0.619** |
| Glomerular mRNA expression of GN-associated genes | *Il1b* |  | 0.589* |
|  | *Il6* |  | 0.709** |
|  | *Tnfa* |  | 0.592* |
| GN-associated histopathological scores | Glo. nuclei |  | 0.645** |
|  | Glo. area |  | 0.772** |
|  | Glo. SC score |  | 0.461* |
|  | Synaptopodin |  | -0.664** |
|  | WT1 |  | -0.225 |
| Values = Spearman's rank correlation coefficient. *: *P* < 0.05. **: *P* < 0.01. *n* = 17-19. Glo: glomerular. ACR: albumin/creatinine ratio. SC: sclerosis. Synaptopodin: the areas exhibiting synaptopodin signals per glomerular area. WT1: the number of WT1-positive nuclei per glomerular area. | | | |
